# pH-responsive nano immunomodulator for rheumatoid arthritis therapy via macrophages pyroptosis inhibiting and reprograming

**DOI:** 10.1016/j.bioactmat.2026.03.031

**Published:** 2026-05-25

**Authors:** Rui Wen, Haoyu Qiu, Pingli Dong, Lanling Dai, Xiaoqin Hu, Fang Lan, Yao Wu

**Affiliations:** National Engineering Research Center for Biomaterials, College of Biomedical Engineering, Sichuan University, Chengdu, 610064, China

**Keywords:** *Pyroptosis inhibition*, *Nanozyme*, *ATP-Adenosine conversion*, *ROS scavenging*, *Rheumatoid arthritis*

## Abstract

Current immunosuppressive therapies for rheumatoid arthritis (RA) are limited by substantial side effects, prompting the need for strategies that remodel the disease microenvironment via immunomodulation. RA progression is driven by a self‐amplifying cycle fueled by elevated extracellular ATP (eATP), reactive oxygen species (ROS) and local acidosis. To disrupt this pathological loop, we developed an intelligent Ce-MOF@CaCO_3_ (Ce-Ca) nano-immunomodulator for RA microenvironment remodeling through a pH-responsive "trigger-regulation-therapy" mechanism. In acidic lesions, the CaCO_3_ shell degrades to neutralize pH and release Ca^2+^, enabling in situ mineralization and repair of bone surfaces while restoring the multiple enzyme-mimicking activities of the Ce-MOF core. This enables a "dual-clearance and dual-modulation" strategy: hydrolyzing eATP and scavenging ROS to suppress the P2X7R-NLRP3 axis and inhibit M1 macrophage pyroptosis, while converting ATP to adenosine to activate the cAMP-PKA pathway and drive macrophage repolarization to the anti-inflammatory M2 phenotype. By further rebalancing the Keap1-Nrf2 axis and suppressing MAPK/NF-κB signaling, the nano-immunomodulator achieves synergistic microenvironment remodeling, integrating pyroptosis inhibition, immunomodulation, and osteogenic repair, offering a promising nanotherapeutic strategy for RA treatment.

## Highlights

• **Acid-triggered Activation:** CaCO_3_ shell degrades in acidic RA lesions, releasing Ca^2+^ for mineralization, and restoring Ce MOF enzyme-mimetic activity.
• **Dual Clearance** & **Modulation:** Ce-MOF core hydrolyzes eATP, scavenges ROS, inhibits M1 pyroptosis, and activates cAMP-PKA to drive M2 macrophage repolarization.
• **Paradigm Shift:** Moves from single-target suppression to systemic microenvironment remodeling, restoring immune balance and tissue repair.

## Graphical abstract

Ce-Ca nanoscale immunomodulators achieve therapeutic efficacy in rheumatoid arthritis by coordinately promoting in situ mineralized osteogenesis and reprogramming the immune microenvironment.

**Figure:**
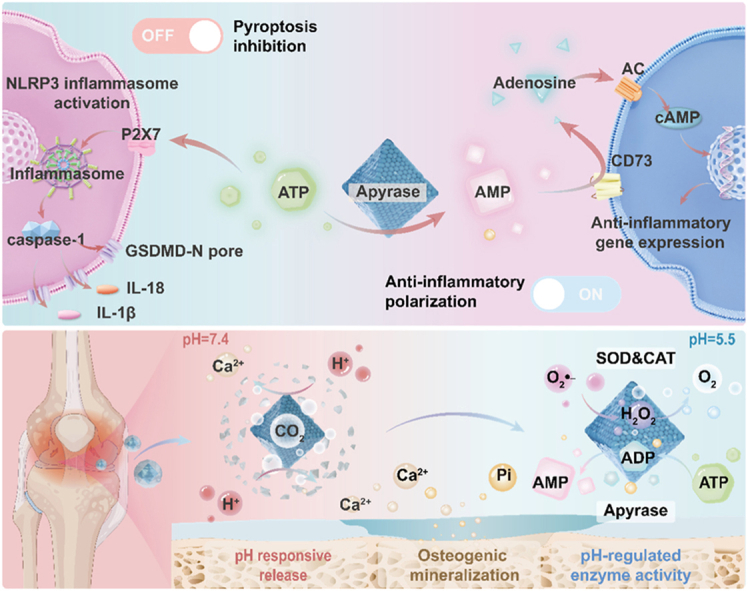

## 1 Introduction

Rheumatoid arthritis (RA) is a chronic, systemic autoimmune disorder characterized primarily by inflammatory synovitis that drives pannus formation and the progressive erosion of cartilage and bone, ultimately resulting in progressive joint destruction, functional impairment, and eventual clinical disability [1]. Contemporary immunotherapy, comprising biologic agents and targeted synthetic disease-modifying antirheumatic drugs (DMARDs) [2], represent a paradigm of precision medicine by inhibiting specific inflammatory cytokines (e.g., TNF-α, IL-6) or key signaling pathways (e.g., JAK-STAT). However, their therapeutic rationale remains rooted in immunosuppression rather than immunomodulation. By blocking single targets, these strategies can induce broad immune impairment, elevate risks of severe infections and malignancies, and crucially fail to remodel the overall dysregulated immune microenvironment due to the high redundancy within RA's pathological network [[3], [4], [5]].

**Scheme 1:**
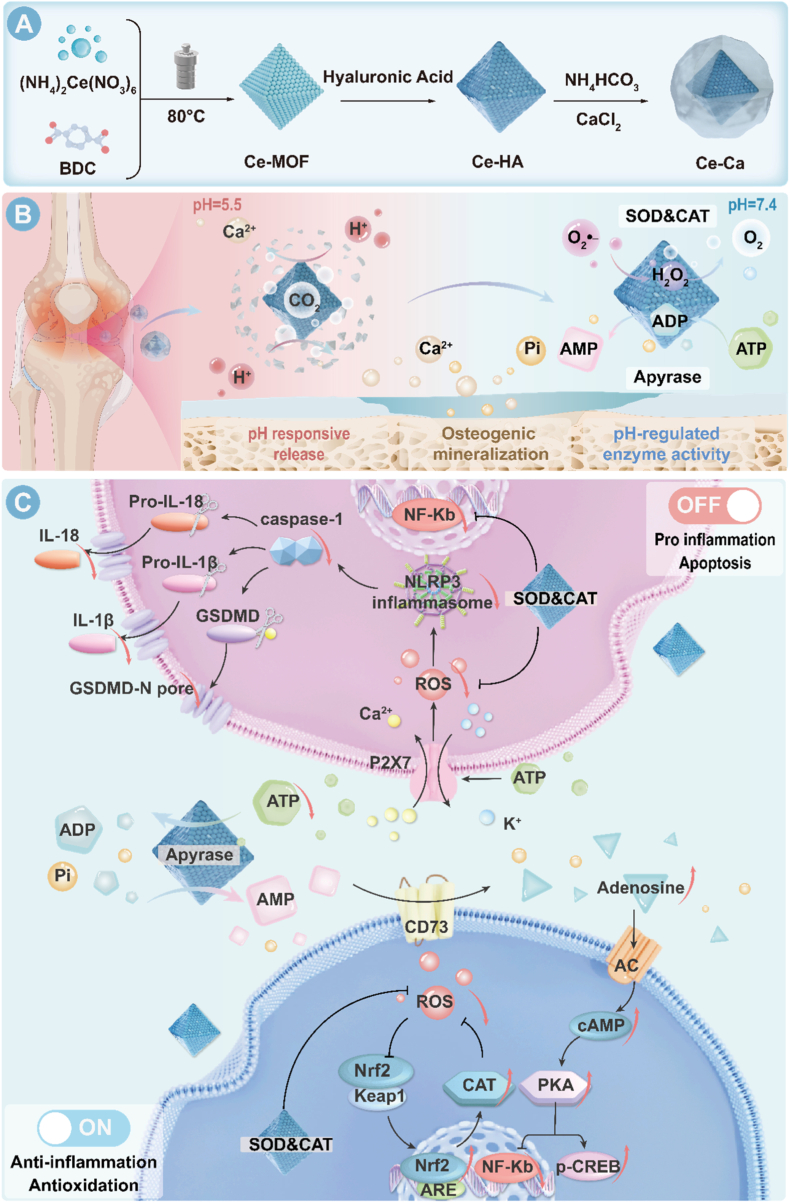
A) Ce-Ca nano-immunomodulators were prepared utilizing a combined method of hydrothermal synthesis and vapor diffusion. B) The nano-immunomodulators were locally administered by intra-articular injection and respond to the acidic microenvironment and enable site-specific release of Ca^2+^ along with in situ mineralization, thereby promoting bone regeneration while restoring the ambient pH to neutral. C) Under neutral pH, the Ce-MOF catalytic core exhibits potent adenosine triphosphate diphosphohydrolase-like and SOD/CAT-like activities, effectively degrading extracellular ATP to facilitate the ATP-adenosine conversion. This process collaborates to reestablish redox homeostasis, suppresses pyroptosis of M1 macrophages in the rheumatoid arthritis microenvironment, and promotes their repolarization toward the M2 phenotype.

At the core of RA progression lies a self-amplifying pathological microenvironment featuring low pH [6], elevated reactive oxygen species (ROS) [7], and high extracellular ATP (eATP) [8]. This triad polarizes macrophages toward a pro-inflammatory M1 phenotype, which releases pro-inflammatory cytokines (e.g., tumor necrosis factor-α (TNF-α), interleukin-6(IL-6) and interleukin-1β (IL-1β)), and exhibits mitochondrial dysfunction that further exacerbate ROS production, disrupting redox homeostasis and activating pro-inflammatory pathways like NF-κB. Furthermore, they exist in a "primed" state with upregulated NLRP3 inflammasome components and interleukin precursors (e.g., pro-IL-1β, pro-IL-18) [9]. Critically, eATP, released from stressed or damaged synovial cells and chondrocytes, acts as a key damage-associated molecular pattern (DAMP) that synergizes with ROS. Upon binding to the P2X7 receptors on M1 macrophages, it triggers NLRP3 inflammasome assembly, caspase-1 activation and pyroptosis [10], leading to massive release of mature IL-1β and IL-18. These cytokines in turn recruit more immune cells and amplify inflammation, establishing a self-perpetuating vicious cycle of "M1 polarization-pyroptosis-inflammation exacerbation" that fuels chronic inflammation and tissue destruction. Therefore, shifting the therapeutic paradigm from single “target inhibition” to comprehensive “microenvironment remodeling” by simultaneously eliminating eATP and ROS to inhibit pyroptosis and restore immune balance is a promising strategy for achieving sustained remission.

Nanozymes, engineered to mimic natural enzyme catalysis, hold great promise for biomedical applications such as cancer therapy, tissue engineering, and autoimmune disease treatment. Among these, cerium-based metal-organic frameworks (Ce-MOFs) stand out with distinct advantages. While inherently the high specific surface area and tunable porosity of MOFs, multi-enzyme activities, such as catalase (CAT) and superoxide dismutase (SOD), are integrated within Ce-MOFs [11,12], enabled by an surface-reversible Ce^3+^/Ce^4+^ redox pair. Moreover, they exhibit ATP hydrolase-like activity, catalyzing the hydrolysis of eATP to Adenosine Diphosphate/Adenosine Monophosphate(ADP/AMP) [13,14]. These combined functionalities enable Ce-MOFs to simultaneously scavenge both ROS and eATP in the RA microenvironment, achieving immunomodulation via directly targeting the self-perpetuating vicious cycle. However, the catalytic activity of Ce-MOFs is strongly pH-dependent. While the Ce^3+^/Ce^4+^ redox cycle functions optimally at neutral pH, the acidic RA microenvironment inhibits electron transfer at the catalytic centers, significantly impairing enzymatic activities [15]. This susceptibility to acidity severely limits their practical therapeutic potential of Ce-MOFs in RA, making the protection of their catalytic sites or even the reversal of local acidosis, is a crucial design challenge.

In the acidic microenvironment of rheumatoid arthritis (RA), the pH-dependent catalytic limitation of conventional inorganic nanozymes [16] (e.g., FeO_X_, MnO_X_, CeO_X_) and the drawbacks of noble-metal nanozymes (e.g., Pt-based, high cost/agglomeration) have become critical bottlenecks for their therapeutic application. Although doping low-valent metal ions (e.g., Y^3+^, La^3+^, Gd^3+^) into CeO_2_ can enhance acidic catalase-like activity [17,18], it compromises biosafety; most existing strategies merely passively adapt to the acidic environment, lacking intelligent designs for active microenvironment regulation or dynamic disease response. In recent years, pH-responsive nanosystems synergized with reactive oxygen species (ROS) scavenging—based on materials such as calcium disilicide [6], metal–organic frameworks (MOFs) [19], carbonates [20], or layered double hydroxides (LDHs) [15,21] loaded with multi-enzyme components/functional elements (e.g., Se)—have been developed to achieve acid neutralization, ROS scavenging, and microenvironment remodeling via pH-triggered activation. While validating the rationality of the "microenvironment response + ROS scavenging" strategy for RA, current studies remain confined to ROS as the sole target, indirectly inducing M2 macrophage polarization without addressing other key pro-inflammatory signaling molecules. Notably, there is a lack of effective blockade against M1 macrophage pyroptosis mediated by highly expressed extracellular adenosine triphosphate (eATP) in the RA microenvironment and its downstream inflammatory amplification loop, which cannot be fundamentally reversed by mere ROS scavenging.

In this study, we constructed an intelligent “nano-immunomodulator” by in-situ coating a calcium carbonate (CaCO_3_) shell on Ce-MOF (denoted as Ce-Ca) to specifically overcome its pH-dependent catalytic limitation (see Scheme 1). The intrinsic multi-enzyme activities of Ce-MOF are readily compromised under acidic conditions, while the CaCO_3_ shell acts as a pH-responsive intelligent protective layer to synergistically reshape the pathological microenvironment of RA. This design strategically employs the acidic RA microenvironment as an endogenous trigger: the CaCO_3_ shell decomposes, neutralizes local acidity to restore the multi-enzyme activities of Ce-MOF, and releases Ca^2+^ to induce in-situ mineralization and promote bone repair. In the neutralized microenvironment, Ce-MOF enables simultaneous efficient hydrolysis of excessive eATP and scavenging of ROS, thereby precisely blocking the P2X7R–NLRP3–GSDMD pyroptosis axis and breaking the vicious cycle of “inflammation-pyroptosis-inflammatory cascade amplification”. Meanwhile, it accelerates the conversion of ATP to adenosine and further activates the adenosine -cAMP anti-inflammatory pathway, forming a dual-pathway synergistic regulatory effect comparable to the combined intervention paradigm of small-molecule drugs. This strategy fundamentally breaks away from the dependence of traditional therapies on single targets and exogenous drugs, and constructs a drug-free nanoscale immunomodulator based on intrinsic enzyme-like catalytic functions. Furthermore, benefiting from its multi-enzyme activities, the system further activates the Keap1/Nrf2 antioxidant axis and inhibits the activation of MAPK/NF-κB signaling pathways, synergistically maintaining mitochondrial homeostasis, and ultimately achieving multi-target, full-chain synergistic intervention in the pathological network of rheumatoid arthritis.Through this integrated “dual-clearance and dual-modulation” strategy, the Ce-Ca nano-immunomodulator successfully realizes the systematic remodeling of the immune microenvironment in RA, providing a synergistic nanotherapeutic strategy that integrates pyroptosis inhibition, immune regulation, and osteogenic repair for RA treatment.

## 2 Results and discussion

### 2.1 Preparation and characterization of Ce-Ca nano-immunomodulator

The Ce-Ca nano-immunomodulator, which exhibits pH responsiveness, neutralizing, and outstanding reactive oxygen species (ROS) scavenging performance, were synthesized. Firstly, Ce-MOFs were fabricated via a hydrothermal method with cerium ammonium nitrate ((NH_4_)_2_Ce(NO_3_)_6_) and BDC serving as the cerium precursor and linker, respectively [14]. Then the Ce-MOFs were functionalized with hyaluronic acid (HA) via electrostatic adsorption in aqueous solution, improving their aqueous dispersibility and endowing them with targeting capability towards M1 macrophages [22]. Finally, a pH-responsive calcium carbonate shell was grown on the Ce-MOFs via a modified gas diffusion method [23], thereby equipping the nano-immunomodulator with microenvironment-responsive functionality.

**Fig. 1:**
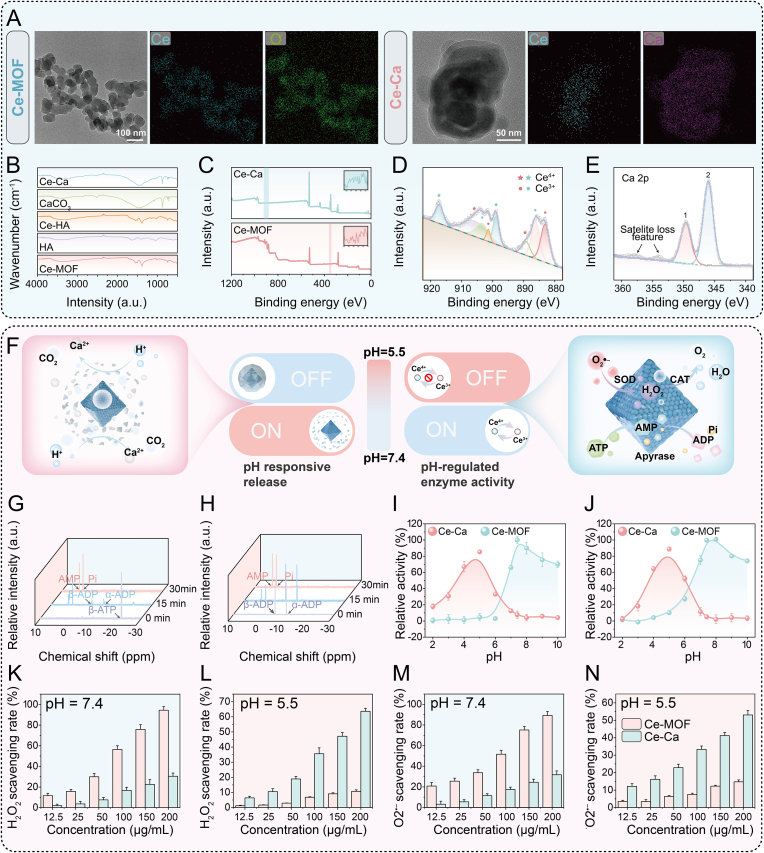
Structural and physicochemical characterization of Ce-Ca nano-immunomodulator and evaluation of its multiple enzyme-mimetic activities under various pH conditions. A) TEM images and element mappings of Ce-MOF and Ce-Ca. B) FTIR spectra, and C) XPS spectra of Ce-MOF, Ce-Ca. D, E) XPS analysis of the Ce 3d spectra of Ce-MOF, Ca 2p spectra of and Ce-Ca. F) Mechanism illustration of calcium carbonate-assisted enhancement of the enzyme-like activity of Ce-MOF with pH-responsive and regulatory functions. ^31^P NMR spectra of G) ATP and H) ADP during catalytic hydrolysis by Ce-MOF under physiological conditions (pH 7.4, 37 °C). I) ATP (125 μM) and J) ADP (125 μM) hydrolysis activities of Ce-MOF (25 mg L^−1^) and Ce-Ca (100 mg L^−1^) under various pH conditions. K) H_2_O_2_ Scavenging activity (CAT-mimic activity) of Ce-MOF and Ce-Ca at pH 5.5 and L) pH 7.4. M) •O_2_^−^ Scavenging activity (SOD-mimic activity) of Ce-MOF and Ce-Ca at pH 5.5 and N) pH 7.4. Data are expressed as means ± standard deviation (SD), n = 3.

The successful construction of the Ce-Ca nano-immunomodulator was verified by Transmission electron microscopy (TEM) and scanning electron microscopy (SEM) (Fig. 1A, Fig. S1), with elemental mapping confirming the co-localization of cerium and calcium signals. Dynamic light scattering (DLS) results indicated that the average hydrodynamic diameters increased from 41.73 nm (Ce-MOF) and 50.16 nm (Ce-HA) to 231.47 nm for Ce-Ca nano-immunomodulator (Fig. S2). Furthermore, zeta potential measurements revealed values of +3.63 mV for Ce-MOF, −13.76 mV for Ce-HA, and +5.07 mV for Ce-Ca (Fig. S3). The significant shift from positive (Ce-MOF) to negative (Ce-HA) potential indicates the successful modification of HA onto the Ce-MOF surface via electrostatic adsorption, while the subsequent positive value of Ce-Ca confirms the effective coating of calcium carbonate. The XRD pattern (Fig. S4) exhibits the characteristic reflections of Ce-MOFs at 2θ = 7.4°, 8.5°, and 25.7°, which can be indexed to its crystal structure, verifying the phase-pure synthesis [14]. Furthermore, distinct diffraction peaks corresponding to calcium carbonate appear in the Ce-Ca sample at 2θ = 23.0°, 29.4°, 36.0°, 39.4°, and 43.2°, with the crystalline peak area accounting for 88.5%, demonstrating the successful preparation of Ce-Ca nano-immunomodulator. As illustrated in the Fourier-Transform Infrared Spectroscopy (FTIR) spectra (Fig. 1B), the composite Ce-HA exhibits characteristic absorption bands corresponding to both Ce-MOF and HA. Meanwhile, all distinctive peaks associated with Ce-MOF, HA, and CaCO_3_ are clearly observed in the spectrum of Ce-Ca. As shown in the X-ray Photoelectron Spectroscopy (XPS) spectra (Fig. 1C–E, S5), the characteristic peaks corresponding to the Ce 3d orbitals in Ce-MOF are consistent with those reported in the literature [12]. Specifically, n detail, the C 1s and O 1s XPS spectra of Ce-MOF and Ce-Ca are provided in Fig. S5. For the C 1s spectrum of Ce-MOF, the peak at 288.6 eV corresponds to carboxylate carbon (O=C–O) — a characteristic of the MOF ligand — while peaks at 285.8 eV and 285.0 eV are assigned to C−O/C=O and sp^2^ C═C, respectively. In contrast, the Ce-Ca C 1s spectrum exhibits a new intense peak at 290.0 eV, which is ascribed to carbonate carbon (CO_3_^2−^) from CaCO_3_, alongside peaks at 285.8 eV (C−O/C=O) and 284.5 eV (adventitious C–C/C–H). Regarding the O 1s spectra, Ce-MOF shows peaks at 533.4 eV (adsorbed oxygen), 531.7 eV (surface chemisorbed oxygen), and 529.8 eV (lattice oxygen). For Ce-Ca, a notably enhanced peak at 532.4 eV (C–O in CO_3_^2−^ from CaCO_3_) is observed, together with surface chemisorbed oxygen at 531.5 eV. Given that XPS is a surface-sensitive technique probing only the outermost few nanometers (approximately 1–10 nm) of a material, the survey spectrum of Ce-Ca displays intense Ca 2p peaks, whereas Ce 3d signals are significantly attenuated. Collectively, these observations provide further evidence for the successful preparation of the Ce-Ca nano-immunomodulator.

### 2.2 Enhanced apyrase-like & antioxidant activities of Ce-Ca nano-immunomodulators under acidic conditions

Ce-MOF, with the abundant Ce^3+^/Ce^4+^ coupling sites and reversible redox cycle, facilitates the selective cleavage of high-energy phosphate bonds (HEPBs) in ATP and ADP under physiological conditions, mimicking apyrase activity. Furthermore, by mimicking the functions of both catalase and superoxide dismutase, it effectively eliminates reactive oxygen species, thereby endowing it with considerable potential for rheumatoid arthritis therapy. However, the multi-enzyme-like activities of Ce-MOF are severely impaired under acidic conditions, limiting its therapeutic efficacy. To address this, we developed an intelligent integrated nano-immunomodulator based on calcium carbonate-coated Ce-MOF, which operates on a "response-regulation-enhancement-therapy" mechanism. By utilizing the acidic microenvironment as an activation trigger, this system enables precise effective component release and pH modulation. As a result, the catalytic activities of Ce-MOF are synergistically enhanced, potently inhibiting pyroptosis for efficient RA treatment (Fig. 1F).

The pH-responsive and regulating properties of Ce-Ca are crucial for its therapeutic function in pathological microenvironments. To systematically characterize its responsive behavior, techniques including TEM, DLS, Zeta potential, and ICP-OES were employed to investigate the structural evolution of Ce-Ca at different pH values. TEM results intuitively showed that the CaCO_3_ shell of Ce-Ca exhibited distinct pH-dependent degradation. The shell structure remained intact under the simulated physiological condition (pH = 7.4). In contrast, in the acidic microenvironment simulating RA lesions (pH = 5.5), the shell degraded gradually over time. Obvious detachment and thinning were observed at 30 min, and the main structure completely disintegrated after 60 min, with only a few CaCO_3_ fragments left on the surface of the Ce-MOF core (Fig. S6). DLS particle size analysis quantitatively verified the above process. At pH 5.5, the particle size of Ce-Ca decreased gradually and stabilized at 50–60 nm after 60 min (Fig. S7), indicating efficient removal of the shell under acidic conditions. At pH 7.4, the particle size remained around 250–280 nm, consistent with the TEM observations. ICP-OES results further confirmed that the Ca^2+^ release rate increased continuously at pH 5.5 and reached 80% at 100 min (Fig. S8), suggesting dissociation of most CaCO_3_ shell. At pH 7.4, the Ca^2+^ release rate was below 10% and remained stable, revealing excellent structural stability under neutral conditions. Zeta potential measurements showed that the surface potential of the material changed from positive to negative with shell degradation and exposure of the inner Ce-MOF (Fig. S9). Multiple characterizations collectively confirmed the typical pH-responsive degradation behavior of Ce-Ca.

Stability studies demonstrated that Ce-Ca maintained a hydrodynamic diameter of approximately 260 nm with PDI below 0.1 for 30 days in both PBS and BSA solutions (Fig. S10), showing good colloidal stability and monodispersity.

Acid-neutralization titration experiments revealed that, compared with Ce-MOF, Ce-Ca significantly increased the solution pH (Fig. S11), exhibiting stronger acid-neutralization capacity. Moreover, Ce-Ca could maintain the system at a neutral pH even after NaOH-induced pH rebound, indicating its precise pH responsiveness and buffering capacity in acidic microenvironments.

We further investigated the catalytic hydrolysis of ATP and ADP by Ce-MOF using ^31^P NMR spectroscopy (Fig. 1G). Under physiological conditions, the characteristic signal of β-ATP diminished within 0.5 h, with the concurrent emergence of signals corresponding to α-ADP, β-ADP, AMP, and phosphate, indicating efficient catalytic conversion. A similar trend was observed when ADP was directly used as the substrate (Fig. 1H). The kinetic curves revealed that Ce-MOF hydrolyzed ATP and ADP at markedly different rates, reaching 81.3% and 29.7% after 30 min, respectively (Fig. S12). Hydrolysis of both nucleotides followed Michaelis–Menten kinetics within suitable concentration ranges (Fig. S13 A-D) [14], confirming efficient and differentiated catalytic behavior. The pH sensitivity of Ce-MOF's catalytic activity toward the hydrolysis of ATP and ADP were evaluated using the malachite green assay (Fig. 1I–J). While optimal hydrolysis of ATP and ADP occurred at pH 7.4, activity dropped sharply under acidic conditions (pH 4.5), retaining only 4.64% and 10.21% of the neutral-pH performance, respectively. In contrast, Ce-Ca maintained high residual activities of 85.27% for ATP and 88.78% for ADP under the same acidic conditions. Although the mass fraction of the catalytic Ce-MOF core in the equal mass of Ce-Ca is lower than that in pure Ce-MOF, the calcium carbonate shell degrades in acid, locally elevating the pH and restoring catalytic activity. However, at pH > 7, the catalytic activity of inner Ce-MOF significantly suppressed due to the blockage of active Ce_6_ clusters by the carbonate layer [14]. Moreover, the relatively large molecular volume of ATP hinders its diffusion through the pores of the carbonate layer. Regarding the antioxidant performance of Ce-Ca, Ce-MOF demonstrates effective scavenging capability towards both H_2_O_2_ and •O_2_^−^ under neutral conditions. However, its ROS scavenging performance was strongly suppressed (Fig. 1K–N) under acidic conditions (pH 5.5). Excessive H^+^ significantly impedes the conversion of Ce^4+^ to Ce^3+^, thereby severely disrupting the redox cycle of Ce-MOF. In contrast, Ce-Ca effectively neutralizes excess H^+^ and initiates a redox cycle reaction under initially low pH conditions, leading to significantly enhanced ROS scavenging performance. Through a synergistic mechanism that combines ATP/ADP hydrolysis with ROS scavenging, this integrated intelligent nanotherapeutic platform effectively operates a “response-regulation-enhancement-therapy” principle, showing great promise as a strategy for treating acid-related pathologies.

### 2.3 Synergistic enzyme-mimetic activities of Ce-Ca nano-immunomodulator target M1 macrophages to alleviate pyroptosis and inflammation

**Fig. 2:**
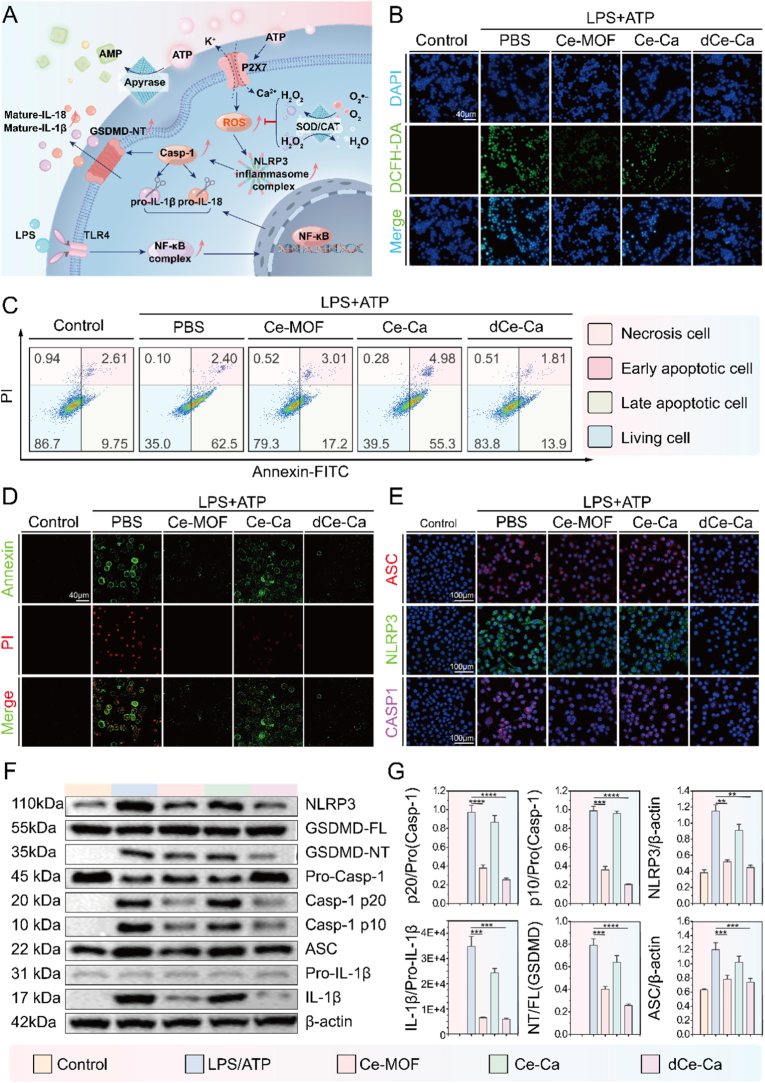
Inhibition of NLRP3 inflammasome activation and pyroptosis by Ce-Ca nano-immunomodulator in LPS/ATP-induced J774A.1 cells. A) Schematic illustration of the synergistic multi-enzyme-like activities of Ce-Ca nano-immunomodulator in suppressing NLRP3 inflammasome activation and pyroptosis in J774A.1 cells. B) DCFH-DA staining images of J774A.1 cells under different treatments. C) Flow cytometric analysis of Annexin V/PI staining in J774A.1 cells after various treatments. D) Confocal microscopy images of Annexin V/PI staining in J774A.1 cells after various treatments. E) Immunofluorescence images showing the NLRP3, ASC, and cleaved caspase-1 expression in J774A.1 cells across treatment groups. F) Western blot analysis of NLRP3, ASC, pro-caspase-1, cleaved caspase-1, GSDMD-FL, GSDMD-NT, pro-IL-1β, IL-1β, pro-IL-18, and IL-18 in J774A.1 cell lysates and G) Semi-quantitative results. Data are expressed as means ± standard deviation (SD), n = 3. Differences were assessed by one-way analysis of variance (ANOVA) followed by Tukey's multiple comparison test. ∗∗∗∗P < 0.0001, ∗∗∗P < 0.001, ∗∗P < 0.01 and ∗P < 0.05.

In the pathological progression of rheumatoid arthritis (RA), aberrant activation and inflammatory death of macrophages represent central processes driving disease advancement. Within the RA microenvironment, mediators such as TNF-α and IFN-γ, polarize macrophages toward a pro-inflammatory (M1) phenotype. These M1 macrophages not only secrete high levels of pro-inflammatory cytokines, such as IL-1β, IL-6, and TNF-α, but also significantly upregulate key intracellular components including the NLRP3 inflammasome and the interleukin precursors pro-IL-1β and pro-IL-18., priming them into a “pre-activated” state poised for exacerbated inflammatory responses. Simultaneously, elevated extracellular ATP (eATP) [24,25], a key damage-associated molecular pattern (DAMP) primarily released from stressed, necrotic, or damaged cells, binds to the purinergic receptor P2X7R, demonstrates upregulated surface expression on M1 macrophages. [26,27]. This activation induces rapid potassium efflux and calcium influx, leading to mitochondrial membrane potential depolarization and the production of mitochondrial reactive oxygen species (mtROS) [28,29]. This process drives the assembly of the NLRP3 inflammasome, leading to Caspase-1 activation and subsequent proteolytic cleavage of Gasdermin D (GSDMD) [30,31], ultimately inducing pyroptosis [32]. The resulting release of mature IL-1β, IL-18 and other inflammatory mediators amplifies the inflammatory cascade and recruits more immune cells, thereby forming a vicious cycle of “M1 polarization-pyroptosis-inflammation exacerbation” that persistently aggravates synovial inflammation. Here, we propose that Ca-Ca nano-immunomodulator suppress pyroptosis in M1 macrophages under high extracellular ATP conditions through the following mechanisms: Degradation of extracellular ATP by a multi-enzyme system suppresses P2X7 overactivation. This action concurrently inhibits receptor-driven ROS generation and potentiates ROS clearance. Consequently, oxidative stress is dually mitigated, attenuating apoptosis and providing synergistic cytoprotection and anti-inflammation. (Fig. 2A).

To elucidate the anti-pyroptotic mechanism of Ce-Ca nano-immunomodulator mediated by their multiple enzyme activities, we established a cellular pyroptosis model in J774A.1 macrophages using LPS and ATP stimulation [25]. As shown in Fig. 2B, intracellular ROS levels were quantified using the ROS-sensitive fluorescent probe 2′,7′-dichlorodihydrofluorescein diacetate (DCFH-DA). Microscopic imaging analysis revealed a significant enhancement of green fluorescence in J774A.1 cells following LPS/ATP treatment, indicating that ATP stimulation effectively induces mitochondrial ROS generation. In contrast, both the Ce-MOF-treated group and the dCe-Ca group (where the calcium carbonate shell was removed via acid degradation) exhibited a marked reduction in fluorescence intensity. Semi-quantitative analysis further revealed a reduction in fluorescence intensity in the dCe-Ca group compared to the Ce-MOF-treated group (Fig. S14). This phenomenon may be attributed to the degradation of the calcium carbonate shell, which facilitates hyaluronic acid (HA)-mediated endocytosis via CD44 receptors overexpressed on M1 macrophages [22]. Confocal laser scanning microscopy (CLSM) visualization and flow cytometric quantification collectively confirmed the proposed targeting mechanism using Cy5-labeled Ce-MOF. In LPS-stimulated M1 macrophages, The Ce-HA group demonstrated markedly enhanced cellular uptake relative to both the Ce-MOF group and the HA + Ce@HA competition group, indicating that HA coating substantially enhances the targeted recognition of Ce-MOF towards M1 macrophages (Fig. S15A–C). In unstimulated cells with low CD44 expression, uptake remained limited, supporting the role of active targeting.

As illustrated by quantitative real-time PCR (RT-PCR), stimulation with LPS significantly upregulated the gene expression of key proteins involved in pyroptosis (Fig. S16), suggesting a "priming" effect of LPS on the pyroptotic pathway. Based on the LDH detection results in the cell culture supernatant, we determined the optimal concentrations of Ce-MOF and dCe-Ca. Both compounds significantly inhibited cell pyroptosis in a dose-dependent manner, exhibiting favorable inhibitory effects at 25 μg/mL and 100 μg/mL, respectively, whereas Ce-Ca showed no such effect (Fig. S17). Scanning electron microscopy (SEM) observations revealed typical morphological features of pyroptosis-cell swelling and bubble-like membrane protrusions in LPS/ATP treated cells, which were alleviated by Ce-MOF or dCe-Ca groups (Fig. S18). To further evaluate the inhibitory effects of the materials on pyroptosis, flow cytometric analysis with Annexin V/PI double staining revealed that both the Ce-MOF and dCe-Ca treatments significantly reduced the populations of both early and late apoptotic cells compared to the LPS/ATP-stimulated group. (Fig. 2C). Corresponding fluorescence staining images revealed a substantial enhancement of Annexin V-FITC (green) and PI (red) fluorescence signals in the positive control group, whereas the fluorescence intensity was markedly attenuated in each material-treated group (Fig. 2D). These findings indicated that Ce-MOF and dCe-Ca effectively suppress pyroptosis and exert a protective effect on cells.

Immunofluorescence analysis was performed to evaluate the expression of key pyroptosis signature proteins within the signaling cascade. The results indicated that LPS/ATP stimulation markedly upregulated expression of NLRP3 and ASC, leading to subsequent activation of Caspase-1, which resulted in an elevated abundance of cleaved Caspase-1. In contrast, the fluorescence signals of these proteins were significantly attenuated in both the Ce-MOF and dCe-Ca treatment groups (Fig. 2E, Fig. S19A). Semi-quantitative analysis further revealed that the fluorescence intensity in the dCe-Ca group was lower than that in the Ce-MOF group (Fig. S19B), suggesting a more potent anti-pyroptotic capacity of dCe-Ca. Flow cytometric analysis similarly confirmed that both Ce-MOF and dCe-Ca effectively suppressed the activation of Cleaved Caspase-1 (Fig. S20). To further validate these findings, the protein expression levels of core pyroptosis mediators were evaluated by Western blot analysis. Consistent with the immunofluorescence observations, treatment with Ce-MOF and dCe-Ca significantly reduced the protein expression of NLRP3, ASC, and activated Caspase-1 (including its cleaved fragments Caspase-1 p20 and p10). Collectively, these alterations thereby suppressed the proteolytic cleavage of GSDMD, resulting in diminished generation of its active N-terminal fragment (GSDMD-N) and a concurrent marked reduction in the levels of mature IL-1β (Fig. 2F–G).

To assess the levels of key effector cytokines released during pyroptosis, we measured the concentrations of mature IL-1β and IL-18 secreted into the cell culture supernatant. The results demonstrated that both Ce-MOF and dCe-Ca treatments significantly inhibited the secretion of these two cytokines (Fig. S21). Collectively, these results demonstrate that Ce-Ca nano-immunomodulator exerts robust anti-pyroptotic effects via a dual enzyme-mimetic mechanism: apyrase-like hydrolysis of burst eATP to abrogate P2X7R-mediated pro-pyroptotic signaling, combined with SOD/CAT-mimetic activities to scavenge excessive intracellular ROS and restore redox homeostasis. This synergistic action not only effectively suppresses NLRP3 inflammasome activation and pyroptotic cell death in M1 macrophages within the local high-eATP microdomains of RA synovium, but also crucially preserves M1 macrophage viability and maintains their phenotypic plasticity for subsequent anti-inflammatory repolarization. This cytoprotective effect provides the essential cellular basis for the nano-immunomodulator to exert further immunoregulatory functions in the moderately reduced eATP microenvironment of the overall RA joint synovium, fully reflecting the precise adaptability of Ce-Ca nano-immunomodulator to the spatial heterogeneity of the RA pathological microenvironment.

### 2.4 Ce-Ca nano-immunomodulator promote M2 macrophage repolarization via a “Convert and Clear” strategy in chronic inflammation

**Fig. 3:**
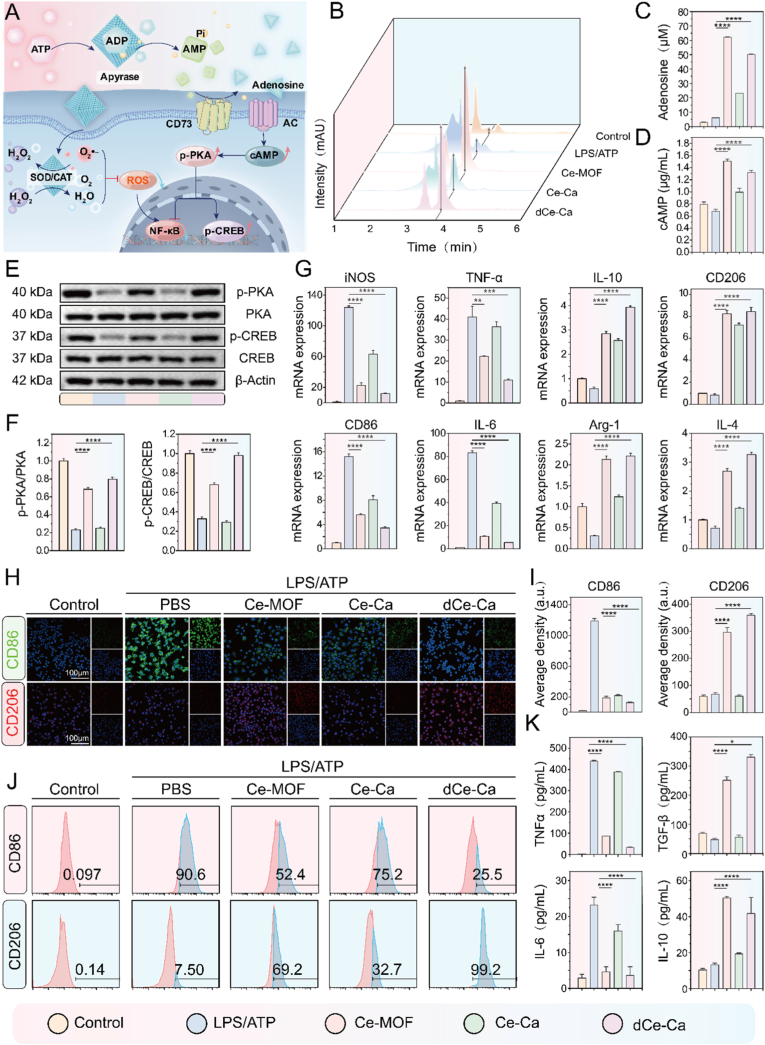
*In Vitro* macrophage repolarization function of Ce-Ca nano-immunomodulator. A) Schematic illustration of the mechanism underlying the ability of the Ce-Ca nano-immunomodulator to restore intracellular redox homeostasis and reverse extracellular pro/anti-inflammatory signaling via multi-enzyme activities to induce M2 macrophage polarization. B) Liquid chromatography profiles showing extracellular adenosine levels across different treatment groups and C) corresponding quantitative analysis. D) Intracellular cAMP concentrations measured by ELISA. E) Western blot analysis of p-PKA, PKA, p-CREB, and CREB expression in cell lysates and F) corresponding Semi-quantitative analysis. G) Quantitative real-time PCR analysis of anti-inflammatory (TGF-β, Arg1, IL-10, CD206) and pro-inflammatory (iNOS, TNF-α, IL-6, CD86) gene expression. H) Immunofluorescence staining of CD86 and CD206 in different groups and I) quantitative analysis of fluorescence intensity. J) Flow cytometric analysis of CD86 and CD206 expression. K) ELISA measurement of IL-6, TNF-α, IL-10 and TGF-β levels in the supernatant. Data are expressed as means ± standard deviation (SD), n = 3. Differences were assessed by one-way analysis of variance (ANOVA) followed by Tukey's multiple comparison test. ∗∗∗∗P < 0.0001, ∗∗∗P < 0.001, ∗∗P < 0.01 and ∗P < 0.05.

In the RA synovial pathological microenvironment, local high-eATP microdomains (formed by burst ATP release from necrotic cells due to aberrant immune attack) and the moderately reduced eATP microenvironment of the overall joint synovium are spatially coexistent heterogeneous features. The moderately reduced eATP in the global synovial microenvironment acts as a persistent purinergic pro-inflammatory signal (around 100 nM) [10,33], which continuously drives macrophage M1 polarization and potently inhibits their transition to the anti-inflammatory M2 phenotype, serving as a core factor for the chronic persistence of synovial inflammation. Based on the confirmed anti-pyroptotic and cytoprotective effects of Ce-Ca nano-immunomodulator in the local high-eATP microdomains (Section 2.3), we further investigated its regulatory function in promoting M2 repolarization in the moderately reduced eATP microenvironment of the overall RA synovium. We hypothesize that Ce-Ca nano-immunomodulators exert a "Convert and Clear" anti-inflammatory effect to drive M2 polarization of M1 macrophages (either survived and phenotype-preserved in high-eATP microdomains or originally present in the moderately reduced eATP microenvironment) through two synergistic mechanisms: (1) ecto-ATP diphosphohydrolase-like activity catalyzes the hydrolysis of eATP to adenosine monophosphate (AMP), which is further converted to adenosine by ecto-5′-nucleotidase (CD73) expressed on the cell surface—this sequential catalytic process accelerates the eATP-to-adenosine conversion, and the generated adenosine, as an important endogenous anti-inflammatory signaling molecule, binds to adenosine receptors (e.g., A2A) on macrophages to initiate anti-inflammatory signaling cascades—activating the intracellular cAMP-PKA signaling pathway and promoting the expression of M2-associated genes; (2) antioxidant activity functions to scavenge residual intracellular ROS, thereby inhibiting the activation of pro-inflammatory signaling pathways like NF-κB and providing a stable anti-inflammatory microenvironment for efficient M2 polarization. This dual mechanism enables the integrated regulation from pyroptosis inhibition and cell protection in local high-eATP microdomains to active promotion of M2 repolarization in the moderately reduced eATP microenvironment of the overall synovium, forming a closed-loop anti-inflammatory effect adapted to the spatial heterogeneity of RA synovium (Fig. 3A).

To test this, we established an inflammatory model using J774A.1 macrophages stimulated with LPS (100 ng/mL) and ATP (100 μM). Liquid chromatography was employed to quantify extracellular adenosine levels. The results demonstrated a marked increase in the characteristic adenosine peak area in both the Ce-MOF and dCe-Ca treatment groups (Fig. 3B–C), supporting eATP-to-adenosine conversion. As a key second messenger, cAMP triggers a signaling cascade by activating protein kinase A (PKA), which directly phosphorylates the transcription factor CREB. This signaling cascade initiates the transcription of anti-inflammatory and reparative genes, including IL-10 and Arg1. ELISA analysis revealed a marked increase in intracellular cAMP levels in these groups (Fig. 3D). Further Western blot analysis (Fig. 3E–F) revealed a significant enhancement in the phosphorylation levels of both PKA and CREB in the Ce-MOF and dCe-Ca groups compared to the LPS/ATP stimulated groups. These results confirmed activation of the adenosine-driven cAMP-PKA-CREB signaling axis. We further treated the cells with an adenosine receptor antagonist (SCH 58261, 100 nM), and the phosphorylation levels of PKA and CREB were significantly decreased in the dCe-Ca + SCH58261 group (Fig. S22). The expression levels of downstream anti-inflammatory and pro-inflammatory genes were assessed using quantitative real-time PCR. Compared to the LPS/ATP control, both treatments promoted a distinct anti-inflammatory gene signature, characterized by elevated expression of M2 markers (e.g., CD206, Arg1) along with key anti-inflammatory cytokines like Il-10. (Fig. 3G). In contrast, the expression of M1 polarization-related pro-inflammatory genes showed a decreasing trend. Notably, the dCe-Ca group exhibited a more pronounced induction of anti-inflammatory factors and M2 polarization-associated genes than the Ce-MOF treatment group alone.

Furthermore, immunofluorescence staining was performed using anti-CD86 (an M1 phenotype marker) and anti-CD206 (an M2 phenotype marker) antibodies to analyze macrophage polarization. The results demonstrated a marked reduction in green fluorescence (indicative of M1 markers) and a significant increase in red fluorescence (representing M2 markers) in both the Ce-MOF and dCe-Ca treated groups compared to the LPS/ATP-stimulated group (Fig. 3H–I). Flow cytometry was subsequently utilized to quantitatively evaluate the ability of dCe-Ca to induce macrophage repolarization. In the LPS/ATP-stimulated M1-positive control group, the percentages of CD86^+^ and CD206^+^ cells were 90.6% and 7.50%, respectively. Treatment with Ce-MOF altered these proportions to 75.2% (CD86^+^) and 69.2% (CD206^+^). Notably, the dCe-Ca group exhibited a substantial reduction in CD86^+^ cells to 25.5%, while CD206^+^ cells were markedly increased to 99.2% (Fig. 3J). ELISA analysis revealed that both Ce-MOF and dCe-Ca treatments elevated anti-inflammatory cytokines (IL-10, TGF-β) while suppressing pro-inflammatory factors (IL-6, TNF-α) (Fig. 3K). Furthermore, immunofluorescence staining and Semi-quantitative analysis of IL-10 and TNF-α corroborated that the anti-inflammatory effect of dCe-Ca was superior to that of Ce-MOF alone (Fig. S23A–B). Collectively, these results demonstrate that dCe-Ca effectively promotes macrophage repolarization toward the M2 phenotype through a dual "Convert and Clear" strategy, thereby significantly mitigating inflammatory responses.

### 2.5 Ce-Ca nano-immunomodulator promote osteogenic differentiation through in situ mineralization and restoration of redox homeostasis

**Fig. 4:**
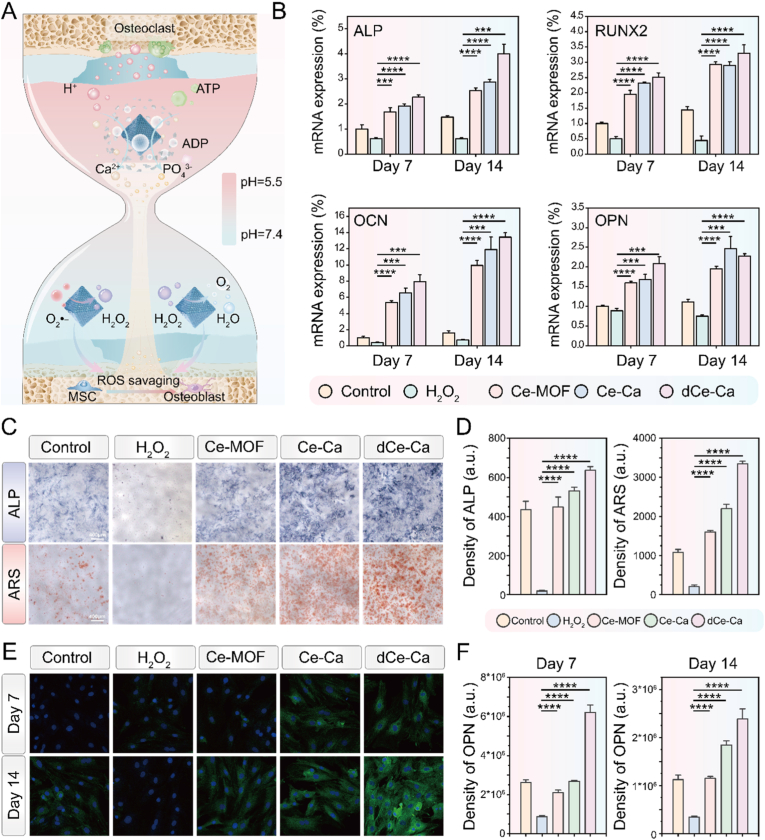
Ce-Ca nano-immunomodulators promote osteogenic activation through in situ mineralization and restoration of redox homeostasis. A) Schematic diagram elucidating the process by which Ce-Ca nano-immunomodulators induce osteogenic activation through microenvironment-responsive release of calcium ions for in situ mineralization, synergized with restoration of redox homeostasis. B) Relative mRNA levels of osteogenic markers (OPN, OCN, RUNX-2) in MC3T3 cells, quantified by qPCR at day 7 and 14 post-treatment. C) Early-stage Alkaline Phosphatase (ALP) staining (day 7) and late-stage mineralization by Alizarin Red S (ARS) staining (day 14) across treatment groups & D) corresponding semi-quantitative results. E) Immunofluorescence staining images of OPN in different groups on 7 days and 14 days & F) corresponding Semi-quantitative results. Data are expressed as means ± standard deviation (SD), n = 3. Differences were assessed by one-way analysis of variance (ANOVA) followed by Tukey's multiple comparison test. ∗∗∗∗P < 0.0001, ∗∗∗P < 0.001, ∗∗P < 0.01 and ∗P < 0.05.

In the articular cavity of RA, synovial tissues exhibit extensive infiltration and activation of immune cells. This process is accompanied by a respiratory burst, leading to a marked increase in ROS, such as H_2_O_2_ and superoxide anions, which disrupts inflammatory cell metabolism and results in the excessive production of acidic metabolites like lactate [6]. The combined effects of high ROS levels and an acidic microenvironment induce apoptosis and senescence of mesenchymal stem cells (MSCs), enhance osteoclast differentiation, and strongly inhibit the osteogenic differentiation capacity of MSCs [34]. Consequently, these alterations disrupt bone homeostasis and accelerate bone loss. Therefore, restoring the osteogenic-osteoclastic balance through the regulation of microenvironmental homeostasis is critical for bone-protective therapy in RA. To address this, we leveraged Ce-Ca nano-immunomodulator which exhibit pH-responsive release of Ca^2+^. These ions cooperate with endogenous phosphate or phosphate generated via ATP hydrolysis to induce in situ bone-like mineralization [23,35]. Concurrently, the Ce-MOF component confers potent antioxidant activity, effectively scavenging ROS and thereby strongly promoting the osteogenic differentiation of MSCs (Fig. 4A).

To evaluate the osteogenic potential of Ce-Ca nano-immunomodulator under inflammatory conditions, we established an RA-mimetic microenvironment by supplementing the osteogenic medium with 400 μM H_2_O_2_. rBMSCs cells were cultured in this medium and treated with various materials for 7 and 14 days. According to RT-qPCR results, H_2_O_2_ stimulation markedly suppressed the expression of osteogenic genes (ALP, OCN, OPN, and RUNX-2). In contrast, all material-treated groups-Ce-MOF, Ce-Ca, and dCe-Ca-signally enhanced the expression of these genes compared to the control group, showing a gradational increase in efficacy in the order of Ce-MOF < Ce-Ca < dCe-Ca. While Ce-MOF primarily scavenges ROS, Ce-Ca nano-immunomodulator are gradually internalized by cells during prolonged incubation, with slow degradation of the calcium carbonate shell enabling sustained Ce-MOF release and intracellular ROS elimination. In the dCe-Ca group, rapid release of calcium ions and immediate exposure of Ce-MOF, exhibited the strongest pro-osteogenic effect (Fig. 4B).

Osteogenic differentiation at early and late stages was further evaluated by ALP staining (7 days) and ARS staining (14 days), respectively. ALP staining revealed weaker intensity in the H_2_O_2_-treated group, while all material groups exhibited markedly intensified staining (Fig. 4C). Quantitative detection of intracellular alkaline phosphatase (ALP) content also showed a similar trend (Fig. S24). In the ARS staining results, apparent mineralized nodule formation was observed in the material-treated groups. Semi-quantitative analysis indicated that the Ce-MOF group restored osteogenic activity to a level comparable to the control group through ROS scavenging. In contrast, both the Ce-Ca and dCe-Ca groups demonstrated enhanced pro-osteogenic effects, due to Ca^2+^-facilitated mineralization (Fig. 4D). Immunofluorescence staining and semi-quantitative analysis revealed a time-dependent increase in osteopontin (OPN) expression across all groups, with the Ce-Ca and dCe-Ca groups exhibiting the most pronounced effects (Fig. 4E–F, S25). Comprehensive osteogenic data demonstrated that dCe-Ca exhibited superior pro-osteogenic capacity compared with Ce-Ca. Acid etching resulted in the rapid release of Ca^2+^, which contributed to the efficient activation of downstream osteogenesis-related pathways. In addition, the rapid exposure of the Ce-MOF core enabled timely scavenging of intracellular and extracellular ROS, thus relieving the inhibition of osteogenic differentiation caused by oxidative stress. The synergistic effect of these two factors was critical for the improved osteogenic performance of dCe-Ca.

These results indicate that Ce-Ca nano-immunomodulator effectively enhance osteogenic differentiation through a synergy between calcium-facilitated mineralization, resulting from the degradation of their carbonate calcium shell, and the outstanding ROS scavenging capacity of Ce-MOF, collectively restoring homeostasis of the cellular microenvironment.

### 2.6 Mechanism of Ce-Ca nano-immunomodulator in anti-pyroptosis through alleviating intra- and extracellular ROS

**Fig. 5:**
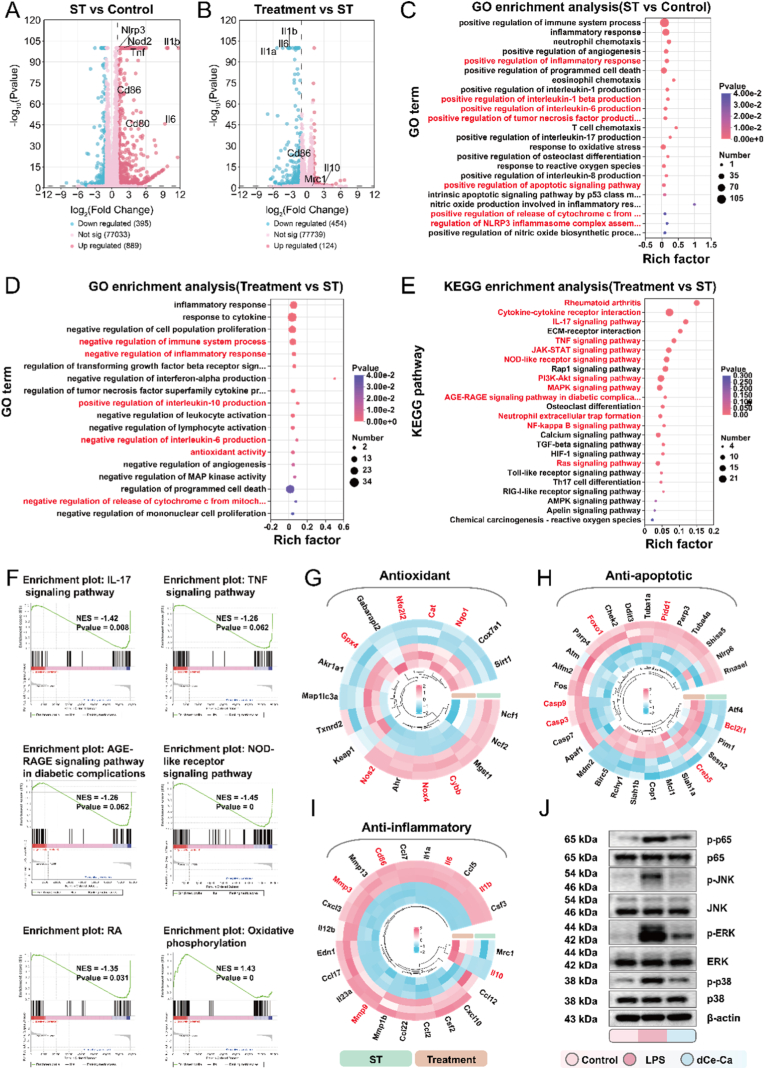
Transcriptomics reveals the anti-pyroptotic effect of Ce-Ca nano-immunomodulator and the underlying molecular mechanism *in Vitro*. A) Volcano plot showing differentially expressed genes (DEGs) between the ST and Control groups. B) Volcano plot showing DEGs between the Treatment and ST groups. Significantly upregulated and downregulated genes are denoted in red and blue, respectively. Control: unstimulated J774A.1 cells; ST: J774A.1 cells stimulated with LPS and ATP to induce a pyroptosis model; Treatment: ST-induced J774A.1 cells treated with dCe-Ca). C) Gene Ontology (GO) enrichment analysis of DEGs from the ST vs. Control comparison and D) the Treatment vs. ST comparison. E) the bubble plot enriched in the KEGG pathway analysis for the Treatment vs. ST group comparison. F) Gene Set Enrichment Analysis (GSEA) of the Treatment vs. ST comparison. G) Heatmaps of I) pyroptosis-related genes, H) antioxidant-related genes, and I) inflammation-related genes in the Treatment vs. ST groups. J) Western blot analysis of p-P65, P65, p-P38, P38, p-JNK, JNK, p-ERK, and ERK in lysates from J774A.1 cells across different treatment groups.

To investigate the regulatory mechanisms of Ce-Ca nano-immunomodulator on LPS/ATP-induced macrophage pyroptosis and repolarization, we performed transcriptome sequencing of J774A.1 cells under different experimental conditions. The experimental design included a Control group, an ST group (stimulated with LPS/ATP), and a Treatment group (LPS/ATP + dCe-Ca nano-immunomodulator). Statistical validation via Pearson correlation and PCA confirmed robust intra-group consistency and distinct inter-group divergence, lending confidence to the reliability of subsequent analyses. (Figs. S26–27). Venn diagrams further highlighted key differences in transcriptional responses across groups (Fig. S28). As shown in the volcano plots (Fig. 5A–B), compared to the Control group, the ST group exhibited 889 significantly up-regulated genes (marked in red), including IL-6, CD86, IL-1β, and TNF-α, which are closely associated with M1 macrophage polarization, pro-inflammatory responses, and pyroptosis. These results confirm the successful establishment of an *in vitro* macrophage pyroptosis model induced by LPS/ATP. Relative to the ST group, the Treatment group exhibited 124 significantly up-regulated genes. including the M2 macrophage polarization marker Mrc1 and the anti-inflammatory cytokine IL-10, while 454 genes were significantly down-regulated, including pro-inflammatory genes such as IL-6, IL-1β, and IL-1α.

To further investigate the functional differences among the groups, we performed Gene Ontology (GO) enrichment analysis. Compared with the Control group, the ST group showed significant enrichment (p < 0.05) in inflammatory and pyroptosis related processes, including upregulation of inflammatory cytokine, NLRP3 inflammasome assembly, programmed cell death, oxidative stress, and cytochrome C release (Fig. 5C). In contrast, the Treatment group demonstrated significant enrichment in a series of negative regulatory processes (Fig. 5D). These results suggest that Ce-Ca nano-immunomodulator effectively scavenge ROS, thereby inhibiting the pyroptosis pathway, reducing the production and release of key inflammatory cytokines (e.g., IL-1β, IL-18), and ultimately disrupting the pro-inflammatory feedback loop.

KEGG pathway enrichment analysis identified significant involvement of the NOD-like receptor signaling pathway, which directly regulates pyroptosis. Additionally, multiple other pathways were enriched, including RA, NF-κB, MAPK, IL-17, AGE-RAGE, and JAK-STAT signaling pathways, as well as neutrophil extracellular trap formation and the PI3K-AKT pathway [27], all of which are closely associated with inflammatory responses, neutrophil chemotaxis, and cell death processes(Fig. 5E–S29). GSEA (Fig. 5F–S30) further revealed significant downregulation of the NOD-like receptor, RA, IL-17, AGE-RAGE, and TNF signaling pathways (NES = −1.45, −1.35, −1.42, −1.26, and −1.26, respectively). Metabolically, the HIF-1α signaling pathway was notably suppressed (NES = −1.08), whereas oxidative phosphorylation was markedly upregulated (NES = 1.43). These results suggest that Ce-Ca nano-immunomodulator may enhance the antioxidative capacity via ROS scavenging, thereby restoring mitochondrial function, suppressing glycolysis, and promoting macrophage polarization toward the M2 phenotype.

Heatmap analysis revealed upregulation of antioxidant genes (e.g., Nfe2l2, Nqo1, Cat and Gpx4) and downregulation of pro-oxidant genes (e.g., Cybb, Ncf1, Ncf2, Nox4 and Nos2) in the Treatment group (Fig. 5G). This phenomenon can be attributed to the excellent SOD/CAT-mimicking activities of Ce-MOF. Consequently, Ce-MOF significantly alleviated intracellular and extracellular ROS stress and disrupted ROS-amplifying pathways such as AGE-RAGE. Furthermore, Ce-Ca nano-immunomodulator restored dynamic balance in the Keap1-Nrf2 axis, alleviating the "antioxidant fatigue" of the Nrf2 pathway and promoting the expression of downstream antioxidant genes such as Cat. This process established a positive feedback loop that enhanced cellular antioxidant capacity [[36], [37], [38]]. The Treatment group also exhibited significant downregulation of genes in the NOD-like receptor signaling pathway (e.g., Rnasel, Gbp2b, Gbp7, Nlrp6, Gbp2, Gbp3, Gbp5, Nod1, and Nod2) which is closely linked to pyroptosis(Fig. S31). In addition, genes associated with mitochondrial damage and cytochrome *c* release (e.g., Apaf1, Casp9, Casp3, Casp7) were also suppressed (Fig. 5H), confirming suppression of both pyroptosis and apoptosis. Inflammatory factors (IL-6, IL-1β), chemokines (CCL2/5/7/12), and matrix metalloproteinases (MMP-3/9/13) were significantly reduced (Fig. 5I). Notably, Ce-Ca nano-immunomodulator promoted eATP-to-adenosine conversion, upregulating the expression of downstream effectors PKA (encoded by Prkacb) and CREB (encoded by Creb5), potentially facilitating NF-κB cytoplasmic retention and transcriptional inhibition. Furthermore, Western blot analysis confirmed that Ce-Ca nano-immunomodulator suppressed phosphorylation of NF-κB, p65, p38, JNK, and ERK (Fig. 5J–S32), indicating blocking ROS-activated MAPK and NF-κB pathways. Consequently, transcriptomic and bioinformatic analyses demonstrate that Ce-Ca nano-immunomodulator scavenge ROS through multi-enzyme activity and multi-pathway regulation, inhibiting downstream pyroptosis and inflammatory signaling cascade, and promoting M2 repolarization.

**Fig. 6:**
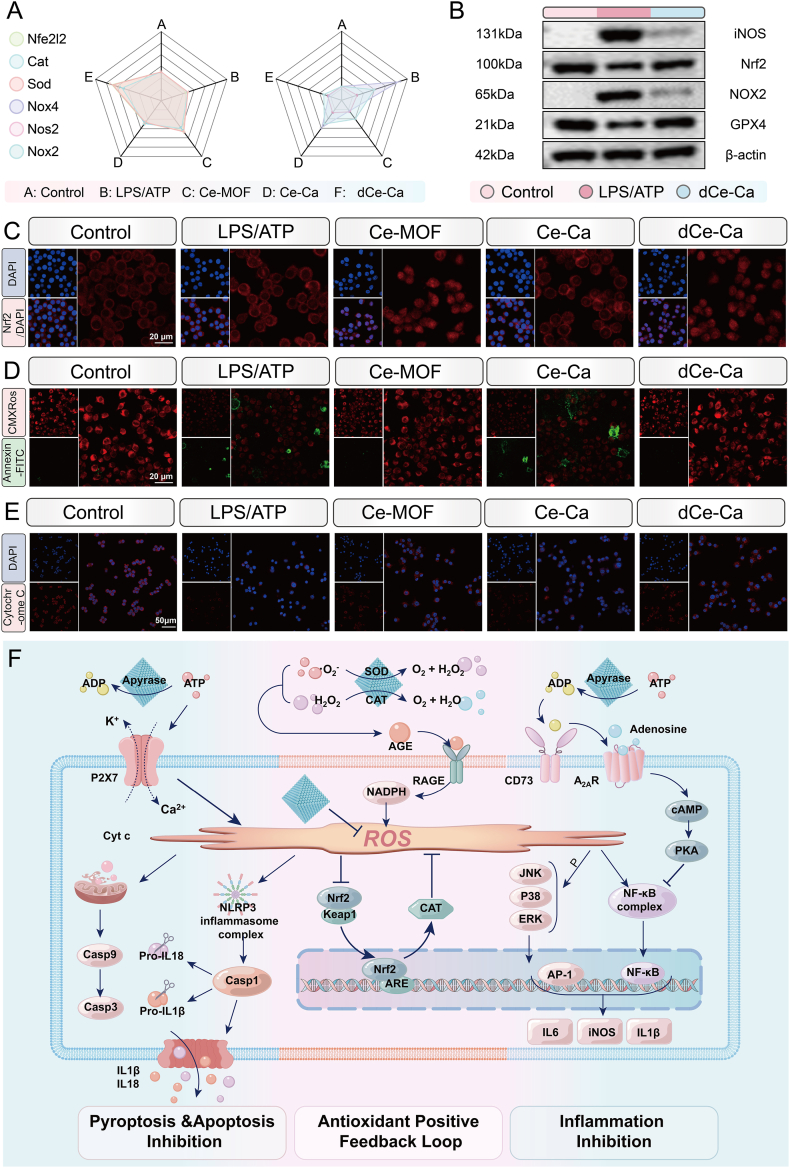
Ce-Ca nano-immunomodulators promote an antioxidant positive feedback loop, restore ROS homeostasis, and confer mitochondrial protection *in vitro*. RT-qPCR analysis assessing the expression levels of relevant antioxidant genes (Nfe2l2, Cat, Sod) and pro-oxidant genes (Nox2, Nox4, Nos2) across different treatment groups. B) Western blotting analysis of iNOS, NOX2, and GPX4 expression in whole-cell lysates, and Nrf2 expression in nuclear extracts from J774A.1 cells under different treatments. C) Immunofluorescence images showing Nrf2 protein localization in J774A.1 cells after various treatments. D) Representative confocal microscopy images of J774A.1 cells stained with Annexin V-FITC and Mito-Tracker Red CMXRos following the indicated treatments. E) Immunofluorescence staining of Cytochrome *c* in J774A.1 cells across different treatments. F) Schematic illustration of the cytoprotective mechanism of Ce-Ca nano-immunomodulator, which involves synergistic multi-enzyme activities to orchestrate intracellular antioxidant positive feedback, anti-pyroptosis, and anti-inflammation. Data are expressed as means ± standard deviation (SD), n = 3. Differences were assessed by one-way analysis of variance (ANOVA) followed by Tukey's multiple comparison test. ∗∗∗∗P < 0.0001, ∗∗∗P < 0.001, ∗∗P < 0.01 and ∗P < 0.05.

Building on these transcriptomic insights, we further validated that Ce-Ca nano-immunomodulator confer mitochondrial protection by multi-enzyme-driven ROS elimination and Nrf2-mediated antioxidant feedback loop. RT-qPCR confirmed downregulation of pro-oxidative genes (Nox2, Nox4, Nos2) and upregulation of antioxidant genes (Nrf2, Cat, Sod) in Ce-MOF and dCe-Ca groups (Fig. 6A). Western blot analysis results further demonstrated a significant upregulation of nuclear Nrf2 and GPX4, alongside a pronounced downregulation of pro-oxidative proteins in the dCe-Ca group (Fig. 6B–S33). To validate the regulatory role of the materials in Nrf2 activation, we further examined the nuclear translocation of Nrf2 via immunofluorescence staining. Confocal Laser Scanning Microscope CLSM results indicated that in the LPS/ATP group, Nrf2 was predominantly localized in the cytoplasm of J774A.1 cells (Fig. 6C). In contrast, the Ce-MOF and dCe-Ca groups exhibited a marked increase in nuclear Nrf2 levels within J774A.1 cells.

Within the *in vitro* pyroptosis model, excessive mitochondrial ROS (mtROS) production triggered by eATP stimulation can induce mitochondrial dysfunction, disrupting ROS homeostasis and establishing a vicious cycle of ROS generation. This process ultimately leads to mitochondrial damage and the release of cytochrome *c*, activating the apoptotic cascade. We evaluated the protective effects of the materials on mitochondria and their anti-apoptotic efficacy by monitoring changes in mitochondrial membrane potential (ΔΨm). Staining with Mito-Tracker Red CMXRos and Annexin V-FITC (Fig. 6D) revealed that the LPS/ATP stimulated group displayed weak red fluorescence (Mito-Tracker Red CMXRos) and intense green fluorescence (Annexin V-FITC), indicating a significant loss of ΔΨm and the induction of apoptosis due to excessive ROS. Conversely, both the Ce-MOF and dCe-Ca groups showed minimal green fluorescence and red fluorescence intensity comparable to the control group, suggesting that the materials effectively facilitated the recovery of ΔΨm. To further evaluate cytochrome *c* release-a key event in mitochondrial damage induced by excessive ROS-we selectively permeabilized the plasma membrane using digitonin, which leaves mitochondrial membranes intact. Under this condition, intracellular fluorescence intensity is inversely correlated with the extent of cytochrome *c* leakage [39]. The results indicated significantly lower cytochrome *c* retention in LPS/ATP-stimulated cells, whereas both Ce-MOF and dCe-Ca treatments markedly enhanced cytochrome *c* retention (Fig. 6E). Furthermore, immunofluorescence staining of Cleaved-Caspase-3 was conducted to confirm the anti-apoptotic effect mediated by mitochondrial protection. CLSM imaging revealed intense red fluorescence, indicating high Cleaved-Caspase-3 expression in the LPS/ATP group. In contrast, both the Ce-MOF and dCe-Ca groups exhibited only minimal fluorescence, demonstrating effective suppression of apoptosis (Fig. S34).

In summary, Ce-MOF exhibits multi-enzyme-mimetic functions: ATP diphosphatase-like activity degrades eATP to attenuate mtROS accumulation and SOD/CAT mimetic functions directly eliminate ROS. Through eATP-to-adenosine conversion, it further blocks pyroptosis via upstream NLRP3 inflammasome inhibition and downstream adenosine-mediated anti-inflammatory signaling. Owing to its outstanding antioxidant capacity, Ce-Ca nano-immunomodulator restore the dynamic balance of the Keap1-Nrf2 signaling axis, promoting the expression of downstream antioxidant genes and establishing a positive antioxidant feedback loop. Meanwhile, phosphorylation of NF-κB p65 and MAPK family proteins is effectively suppressed, leading to the inhibition of associated inflammatory signaling pathways. Ultimately, Ce-Ca nano-immunomodulator exerts multiple therapeutic effects, including antioxidation, inflammation suppression, and pyroptosis blockade (Fig. 6F).

### 2.7 Therapeutic efficacy of Ce-Ca nano-immunomodulator in AIA model

**Fig. 7:**
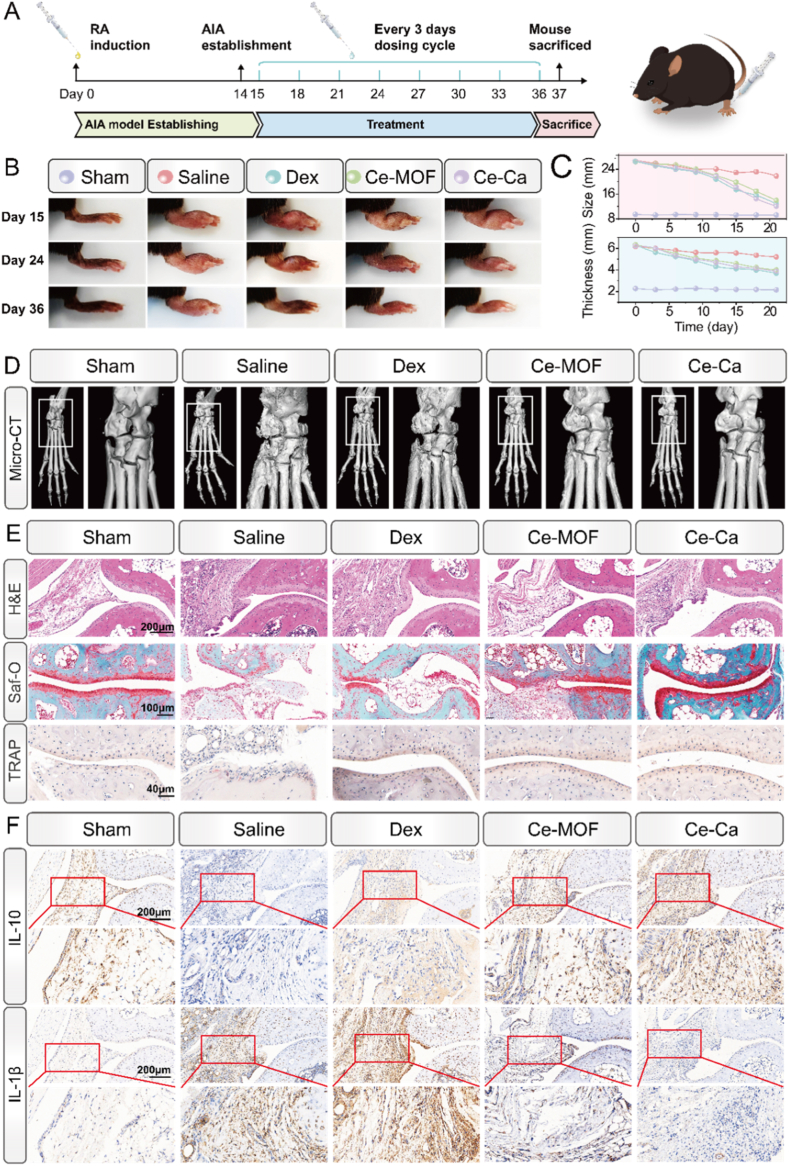
*In vivo* therapeutic efficacy of Ce-Ca nano-immunomodulator in AIA mouse model. A) Schematic timeline of the intra-articular injection treatment regimen. B) Representative photographs of hind paws from the different treatment groups during the therapeutic period. C) Quantitative measurements of hind paw circumference and thickness across treatment groups. D) Three-dimensional reconstructed micro-CT images of hind paws from each treatment group. E) Representative images of H&E, Safranin O/Fast Green, and TRAP staining in joint tissues from different treatment groups. F) Immunohistochemical staining of IL-10 and IL-1β in joint sections across groups. Magnified views of the synovial regions outlined in red are displayed directly below their respective panels.

To evaluate the therapeutic efficacy of Ce-Ca nano-immunomodulator in RA, an adjuvant-induced arthritis (AIA) mouse model was established using complete Freund's adjuvant (CFA) [40,41](Fig. 7A). AIA mice were randomly divided into five groups receiving intra-articular injections every three days: sham, saline, Dexamethasone (Dex, positive control), Ce-MOF, and Ce-Ca. By day 14 post-modeling, the hind paw ankles and toe regions of AIA rats exhibited redness and swelling, confirming successful model induction (Fig. 7B–S35). After day 21 of treatment, joint swelling was markedly reduced in the Ce-Ca group, with paw circumference and thickness recovering to near-normal levels (Fig. 7C). Infrared thermography showed a significantly elevated temperature in the hind paws of the saline group, indicating active inflammation, while the Ce-Ca group exhibited normalized thermal profiles (Figs. S36–37).

To verify the pH regulation ability of Ce-Ca in the pathological joint microenvironment, pH-sensitive probes were used for semi-quantitative detection of synovial fluid pH. Briefly, fluorescence emission curves of probes at the same concentration were measured under different pH gradients to establish a standard calibration curve. With the fluorescence intensity of normal joints from untreated mice as reference, the pH value of synovial fluid in each group was calculated by fluorescence ratio method. The results showed that the fluorescence signal in inflamed joints of AIA mice was significantly decreased with a pH of approximately 5.5, while the fluorescence intensity was remarkably restored to normal levels after Ce-Ca intervention. These results provided direct and reliable evidence for the “response-regulation-therapy” functional strategy of Ce-Ca (Fig. S38).

Beyond macroscopic observations, micro-CT was employed to assess bone erosion-a core pathological feature of RA. Severe bone loss and surface roughness, extending from the calcaneus to the phalanges, were observed in the ankle joints of the saline group. While the Dex and Ce-MOF groups exhibited moderate therapeutic effects, Ce-Ca group best preserved bone architecture (Fig. 7D), with quantitative analysis confirming superior bone-protective efficacy (Fig. S39).

Based on the histological evaluation of a RA model, this study further analyzed the pathological improvements across different treatment groups. H&E staining showed synovial hyperplasia, lymphocyte infiltration, pannus formation, and bone erosion in the saline group (Fig. 7E). Both the Dex and Ce-MOF groups exhibited attenuated joint pathology, though synovial hyperplasia persisted. In contrast, the Ce-Ca group maintained near-normal joint architecture without obvious bone erosion or pannus. Safranin O-Fast Green staining indicated severe glycosaminoglycan loss in the cartilage of the saline group, suggesting proteoglycan degradation and cartilage damage, whereas the Ce-Ca group preserved cartilage integrity. TRAP staining demonstrated a marked increase in osteoclast numbers in the saline group, which was significantly suppressed in the Ce-Ca group. Collectively, semi-quantitative histopathological scoring of synovial hyperplasia (Fig. S40), inflammatory cell infiltration, joint structure destruction, cartilage matrix loss, and TRAP-positive area indicated that the Ce-Ca nano-immunomodulator exerted a significant ameliorative effect on joint inflammation and bone destruction in adjuvant-induced arthritis (AIA) mice.

To further investigate the expression changes of proteins associated with pyroptosis and inflammation, we performed immunohistochemical analysis. The results showed a significant upregulation of RUNX2 (Figs. S41–42), a key osteogenic marker, in the Ce-Ca group compared with the saline group, indicating enhanced osteogenic potential. Regarding inflammatory cytokines (Fig. 7F–S43), the saline group exhibited markedly increased expression of pro-inflammatory factors IL-1β and IL-18, along with significantly decreased expression of anti-inflammatory factors IL-10 and TGF-β. In contrast, both the Ce-MOF and Ce-Ca groups showed substantial reduction in pro-inflammatory cytokine levels and a notable increase in anti-inflammatory cytokine expression. Importantly, the expression profile in the Ce-Ca group was nearly restored to levels comparable to the normal control group (Fig. S44).

**Fig. 8:**
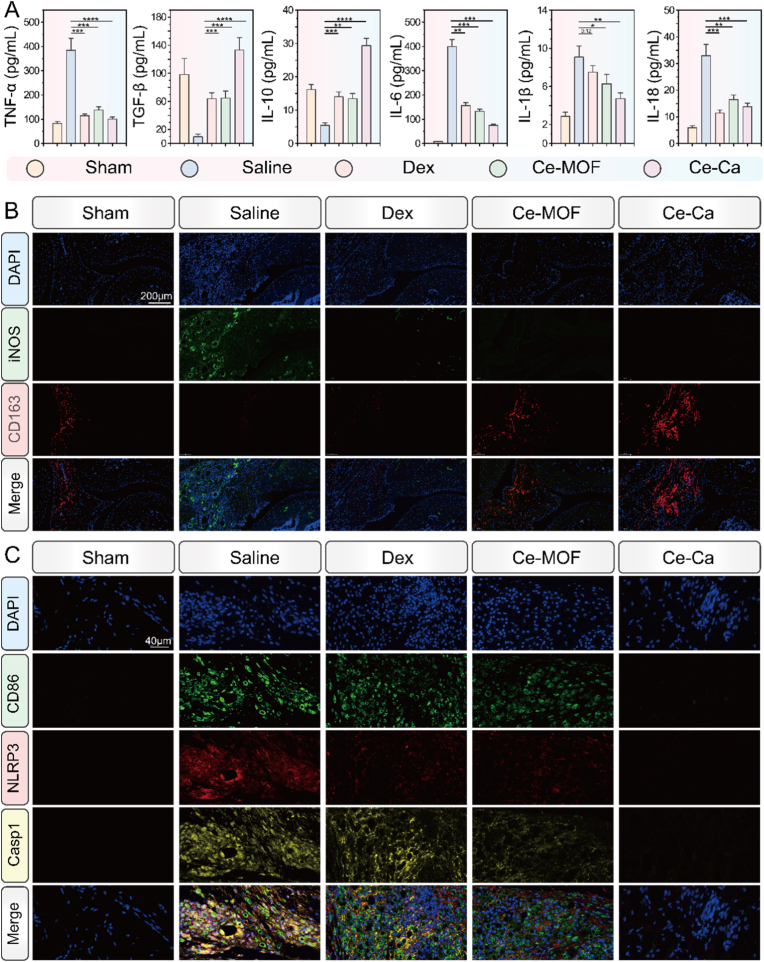
Therapeutic effects of Ce-Ca nano-immunomodulator on rheumatoid arthritis microenvironment (RAM). A) ELISA results of serum levels of TNF-α, TGF-β, IL-10, IL-1β, IL-6, and IL-18 in different treatment groups. B) Proportions of M1 (iNOS^+^, green) and M2 (CD163^+^, red) macrophages in joint tissues of mice from different treatment groups. C) Immunofluorescence staining of NLRP3 and cleaved-Caspase-1 in M1 macrophages within the joint sections across treatment groups.

Furthermore, ELISA confirmed systemic anti-inflammatory effects, with serum concentrations of TNF-α, IL-6, IL-1β and IL-18 reduced, and IL-10, and TGF-β elevated in treatment groups (Fig. 8A). To further investigate the impact of the material on macrophage phenotype polarization and pyroptosis in the AIA model, immunofluorescence analysis was conducted for M1/M2 macrophage markers and key pyroptosis-related proteins. As shown in Fig. 8B, the saline group exhibited strong expression of the M1 marker iNOS, whereas Dex, Ce-MOF and Ce-Ca NP treatments significantly suppressed iNOS expression. In contrast, expression of the M2 marker CD163 was generally upregulated in the treatment groups (Fig. S45), with the Ce-Ca group showing the highest CD163 level. Further co-localization staining of NLRP3, Cleaved-Caspase-1, and the M1 macrophage marker CD86 revealed markedly elevated expression of NLRP3 and Cleaved-Caspase-1 in the saline group (Fig. 8C–S46), with extensive co-localization with CD86, suggesting a critical role of M1 macrophage pyroptosis in RA inflammation progression. Among all treatment groups, the Ce-Ca group displayed the lowest expression of NLRP3 and Cleaved-Caspase-1, indicating the most potent inhibitory effect on pyroptosis. Therefore, Ce-Ca nano-immunomodulators effectively scavenge reactive oxygen species (ROS) through their multi-enzyme-like activities, thereby restoring homeostasis in the rheumatoid arthritis microenvironment. Concurrently, they suppress M1 macrophage pyroptosis and promote polarization toward the M2 anti-inflammatory phenotype, which collectively mitigates inflammatory amplification and facilitates tissue repair and osteogenesis.

### 2.8 Biosafety assessment

To comprehensively evaluate the biosafety of Ce-Ca nano-immunomodulator, we conducted a series of *in vitro* and in vivo experiments. For *in vitro* safety assessment, the cytotoxicity of Ce-MOF, Ce-Ca and dCe-Ca on cells (J774A.1, ATDC5 and rBMSC) was assessed using the CCK-8 assay. After incubating the cells with varying concentrations of Ce-Ca for 24 h and 48 h, the results (Fig. S47) demonstrated that neither Ce-MOF, Ce-Ca, nor dCe-Ca (which degrades the calcium carbonate shell) exhibited significant cytotoxicity at concentrations up to 100 μg/mL. Cell viability remained above 85% in all cases, indicating good biocompatibility of the materials (Fig. S47). In the *in vitro* chronic inflammation model established by LPS combined with low-concentration ATP (100 μM), we further detected the dynamic changes in the levels of related components including ATP, ADP, AMP and adenosine. In the dCe-Ca group, the intermediate products ADP and AMP only showed transient accumulation (Fig. S48A–B). CCK-8 results also demonstrated that the materials themselves and the intermediates produced during the functional process exhibited satisfactory biosafety (Fig. S48C). In addition, hemolysis assays confirmed that neither Ce-MOFs nor Ce-Ca nano-immunomodulator induced obvious hemolysis at various concentrations (Fig. S49). To determine the guidance dose for animal experiments, we further performed acute toxicity testing of the material. The results showed that Ce-Ca at a dose of 20 mg/kg had no significant effects on the serum biochemical indexes (ALT, AST, ALP, CREA, BUN, GGT), body weight, liver tissue weight, or routine blood parameters in mice (Fig. S50A–F).

The local retention and clearance behavior of Ce-Ca after intra-articular injection into the knee joint was evaluated by in vivo fluorescence imaging. As shown in Fig. S51A, obvious fluorescence signals could still be observed at the injection site 168 h (7 days) post-injection, indicating the prolonged retention property of the material in the joint cavity. Quantitative analysis of fluorescence intensity (Fig. S5B) revealed a gradual decay of the signal over time, confirming the gradual clearance of the material from the joint cavity. These results demonstrated that Ce-Ca exhibited favorable long-term local retention, while the gradual attenuation of the signal indicated no permanent accumulation in the joint.

To track the in vivo distribution of the nanozyme, major organs (heart, liver, spleen, lung, kidney, intestine, and paw) were collected at different time points post-injection (12, 48, 72, and 168 h) for ex vivo fluorescence imaging (Fig. S52A). The results showed that fluorescence signals were mainly distributed in the liver, kidney, and spleen, suggesting that the nanoparticles might enter the systemic circulation from the joint cavity through microvessels and lymphatic pathways and accumulate in these organs. Over time, the fluorescence intensity in these organs gradually decreased after 48 h, whereas the fluorescence signal in the intestine was significantly enhanced after 48 h (Fig. S52B). Considering that the size of the Ce-MOF core is larger than 10 nm (difficult to be filtered by the kidney), we speculate that the nanoparticles may be metabolized by the liver, excreted into the intestine through bile, and subsequently eliminated from the body. Furthermore, ICP-MS was used to quantitatively determine the Ce content in major organs at 12, 48, and 168 h (Fig. S52C) to verify the clearance behavior of Ce-Ca. The results showed that the Ce concentration in all organs decreased significantly within the 168h observation period, and the distribution trend was generally consistent with the fluorescence imaging results. At 168 h, the Ce content in each organ was close to that of the control group, indicating that Ce-Ca nanoparticles could be gradually metabolized and cleared in vivo.

For the long-term biocompatibility of the Ce-Ca nano-immunomodulator, histopathological examination of major organs was performed via H&E staining after animal experiments. The results showed normal tissue morphology and architecture, with no evident pathological alterations or organic injury (Figs. S53–54). Hematological analysis further indicated that the key hematological parameters of experimental animals in all groups were within normal physiological ranges, with no significant abnormal fluctuations. These results from systemic safety evaluations demonstrate that the Ce-Ca nano-immunomodulator possess favorable biocompatibility and provide reliable evidence for their safety in therapeutic applications.

## 3 Conclusion

In this study, an integrated intelligent nanotherapeutic platform, designated Ce-Ca nano-immunomodulator, was successfully constructed based on a “Response-Regulation-Enhancement-Therapy” strategy for synergistic RA treatment. This nanoplatform specifically responds to the acidic RA microenvironment via its CaCO_3_ shell, which degrades to neutralize local pH and enhances the ATP diphosphohydrolase-like and SOD/CAT-mimetic activities of the Ce-MOF core. This synergy achieves multiple therapeutic effects: On one hand, Ce-Ca nano-immunomodulator restore ROS homeostasis in M1 macrophages and efficiently degrade eATP, thereby interrupting the “inflammation-pyroptosis-further inflammation” positive feedback loop. On the other hand, they promote the ATP-adenosine conversion, activating the cAMP-PKA signaling pathway to drive anti-inflammatory M2 macrophage repolarization. Simultaneously, Ca^2+^ ions released from the decomposition of CaCO_3_ shell combine with endogenous or ATP-derived phosphate to induce in situ mineralization on the eroded bone surface, activating bone repair. Transcriptomic and analyses further demonstrated that Ce-Ca nano-immunomodulator rebalance the Keap1-Nrf2 axis through their antioxidative properties, establishing a positive feedback loop of antioxidant responses. This process helps protect mitochondrial function and suppresses both pyroptosis and apoptosis. Moreover, the platform effectively inhibits the activation of NF-κB and MAPK signaling pathways, thereby achieving integrated and synergistic regulation in suppressing M1 macrophage pyroptosis, enhancing cytoprotective effects, and promoting anti-inflammatory phenotypic transition. In summary, Ce-Ca nano-immunomodulators demonstrate the potential for multi-pathway and multi-target synergistic intervention in RA treatment, offering a new perspective for nanotechnology-based therapeutic strategies against RA.

## CRediT authorship contribution statement

**Rui Wen:** Writing – original draft, Validation, Software, Methodology, Investigation, Formal analysis, Data curation, Conceptualization. **Haoyu Qiu:** Validation, Software, Resources, Formal analysis. **Pingli Dong:** Software, Resources, Formal analysis. **Lanling Dai:** Software, Resources, Formal analysis. **Xiaoqin Hu:** Software, Resources, Investigation. **Fang Lan:** Writing – review & editing, Supervision, Funding acquisition. **Yao Wu:** Supervision, Project administration, Funding acquisition, Conceptualization.

## Ethics approval and consent to participate

All animal experimental protocols involved in this study were approved by the Experimental Animal Management and Ethics Committee of Sichuan University (Chengdu, China) (Approval No.: [K2024021]). The procedures strictly adhered to animal welfare guidelines and complied with relevant ethical standards throughout the experiment.

## Declaration of competing interest

The authors declare that they have no known competing financial interests or personal relationships that could have appeared to influence the work reported in this paper.

## Data Availability

Data will be made available on request.

## Appendix A Supplementary data

Synthesis and Characterization of Ce-MOF and Ce-Ca nano-immunomodulator; Evaluation of their pH-Regulating Capacity In Vitro; Assessment of ATP/ADP Degradation by Ce-MOF and Ce-Ca nano-immunomodulator In Vitro; Cell Culture and In Vitro Evaluations including Cell Viability, Antioxidant Properties, Pyroptosis Inhibition, and Anti-inflammatory Macrophage Polarization; Investigation of the Osteogenic Differentiation Effects Induced by Ce-MOF and Ce-Ca nano-immunomodulator, encompassing specific methodologies such as Immunofluorescence, Western Blotting, ELISA, and q-PCR along with corresponding data analysis; Transcriptomic Analysis of relevant experimental groups; and Immunohistochemical Staining of target indicators in animal models post-treatment.
