# Supplementary material for: pH-responsive nano immunomodulator for rheumatoid arthritis therapy via macrophages pyroptosis inhibiting and reprograming
Source: Bioact Mater. 2026 May 25;64:799–816. doi: 10.1016/j.bioactmat.2026.03.031 (PMC13226831; doi:10.1016/j.bioactmat.2026.03.031)
Supplement: Multimedia component 1 [file mmc1.docx]

**Supporting Information**

**pH-Responsive Nano Immunomodulator for Rheumatoid Arthritis Therapy via Macrophages Pyroptosis Inhibiting and Reprograming**

**1. Chemicals and Materials**

Cerium (IV) ammonium nitrate ((NH_4_)_2_Ce(NO_3_)_6_) was purchased from Shanghai Macklin Biochemical Co., Ltd. 1,4-dicarboxybenzene (BDC), 4-(2-hydroxyethyl) piperazine-1-ethanesulfonic acid (HEPES)and hyaluronic acid（HA）were purchased from Shanghai Aladdin Biochemical Technology Co., Ltd. CaCl_2_·H_2_O was purchased from the Sigma-Aldrich. Ethanol, NH_4_HCO_3_ and concentrated hydrochloric acid were purchased from the Shanghai Reagent Company Co., Ltd. Adenosine triphosphate (ATP), adenosine diphosphate (ADP), and adenosine monophosphate (AMP) were purchased from Shanghai Bidepharm Co., Ltd. Superoxide dismutase (SOD) Assay Kit (NBT Method) and Catalase (CAT) Assay Kit (Ammonium Molybdate Colorimetric Method) were purchased from Shanghai mlbio Co., Ltd. Malachite Green Phosphate Detection Kit, reactive oxygen species assay kit and cell counting kit-8 (CCK-8) reagent were purchased from Jiangsu Beyotime Biotechnology Co., Ltd. Complete Freund’s adjuvant (CFA) and Lipopolysaccharides (LPS) were purchased from Sigma-Aldrich.

**2. Material Fabrication and Characterization**

**2.1 Synthesis of Ce-MOF**

Ce-MOF was synthesized via a solvothermal reaction. Briefly, ((NH_4_)_2_Ce(NO_3_)_6_) (2.34 g, 4.27 mmol) was dissolved in 8 mL of deionized water, followed by the addition of 244 μL of acetic acid. The mixture was stirred and heated at 60 °C for 30 min. After cooling, 1,4-benzenedicarboxylic acid (H_2_BDC, 0.71 g, 4.27 mmol) was dissolved in 37.52 mL of N, N-dimethylformamide (DMF) and combined with the cooled cerium solution. The resulting mixture was stirred and heated at 80 °C for another 30 min. After cooling to room temperature, the yellow precipitate was collected by centrifugation, washed three times with DMF, and then soaked in DMF for 12 h. The product was subsequently immersed in acetone to remove residual DMF. Finally, Ce-MOF powder was activated under vacuum for further characterization.

**2.2 Synthesis of Ce-Ca**

First, 20 mg of the as-synthesized Ce-MOF was dispersed in 10 mL of reverse osmosis (RO) water under ultrasonication. Then, 5 mL of 1 wt% hyaluronic acid (HA, 300 kDa) was slowly added dropwise into the dispersion under continuous stirring, allowing HA to coat the Ce-MOF surface via electrostatic adsorption. After 1 h, the resulting Ce-HA composite was collected by centrifugation, washed three times with RO water, and redispersed in 1 mL of RO water for storage. Ce-Ca was subsequently synthesized via a vapor diffusion method. In brief, 100 mg of CaCl_2_·H_2_O was dissolved in 50 mL of anhydrous ethanol, followed by the addition of 500 μL of the Ce-HA dispersion under stirring. The mixture was placed in a desiccator containing a glass vial filled with ammonium bicarbonate. The desiccator was sealed and maintained for 24 h, leading to the formation of white Ce-Ca nanospheres. The final product was collected by centrifugation, washed three times with ethanol, and stored in ethanol.

**2.3 Material Characterization**

The Ce-MOF and Ce-Ca nanomaterials were systematically characterized by scanning electron microscopy (SEM, Hitachi S-4800) and transmission electron microscopy (TEM, FEI Tecnai G2 F20) to analyze their microstructure and morphology, dynamic light scattering (DLS, Malvern Zetasizer Nano ZS) to determine the hydrodynamic size and zeta potential for assessing colloidal stability, Fourier-transform infrared spectroscopy (FT-IR, Bruker INVENIO R) to identify surface functional groups and chemical bonds, X-ray diffraction (XRD, Malvern Panalytical EMPYREAN) to determine the crystal structure, X-ray photoelectron spectroscopy (XPS, Thermo ESCALAB 250Xi) to analyze the elemental composition and chemical states.

**2.4 Monitoring ATP and ADP Hydrolysis Catalyzed by Ce-MOF via ^31^P-NMR Spectroscopy**

The hydrolysis of ATP and ADP catalyzed by Ce-MOF was monitored using ³¹P-NMR spectroscopy. Specifically, HEPES (14.3 mg, 0.06 mmol) was dissolved in 10 mL of D₂O, followed by the addition of ATP (27.56 mg, 0.05 mmol) or ADP (21.4 mg, 0.05 mmol). The pH of the solution was adjusted to 7.4 using NaOH. Subsequently, 1 mg of Ce-MOF powder was added to the buffer solution, and the mixture was stirred at 37 °C. Aliquots (1 mL) of the suspension were withdrawn at various time points (0, 15, and 30 min), filtered through a 0.22-μm membrane, and analyzed by ^31^P-NMR to track the reaction progress.

**2.5 Evaluation of ATP and ADP Hydrolysis by Ce-MOF Using the Malachite Green Assay**

The hydrolytic activity of Ce-MOF toward ATP and ADP was assessed by detecting inorganic phosphate release via the malachite green method. Reactions were performed in HEPES buffer (20 mM, pH 7.4) with Ce-MOF concentrations of 12.5, 25, and 50 mg·L⁻¹, and ATP or ADP concentrations ranging from 15.6 to 1000 μM. After incubation in a shaking incubator (37 °C, 150 rpm) for 5 min (ATP) or 30 min (ADP), the reaction mixtures were filtered through a 0.22μm membrane. A 200μL aliquot of the filtrate was mixed with 70 μL of malachite green reagent and incubated for 30 min. The absorbance at 630 nm was measured using a microplate reader. Phosphate concentrations were determined from a standard curve generated with NaH_2_PO_4_. The initial reaction rate (V) for each substrate concentration was calculated as:

Reaction rate = Amount of phosphate product / Reaction time

The kinetic parameters were derived by fitting the experimental data to the Michaelis–Menten model using the linearized form:

1/V = (Kₘ/Vₘₐₓ) × (1/S) + 1/Vₘₐₓ

where V (μM·s^-1^) represents the initial reaction rate of phosphate generation, S (μM) is the substrate concentration (ATP or ADP), V_max_ (μM·s^-1^) denotes the maximum reaction rate, and K_m_ (μM) is the Michaelis-Menten constant.

**2.6 Acid Neutralization Capacity of Ce-Ca**

The acid neutralization capacity was evaluated by titrating a PBS buffer (initial pH 4.5) with 1 mg/mL suspensions of Ce-MOF and Ce-Ca, alongside PBS and 0.01 M NaOH as controls. Aliquots of 10 μL were sequentially added under continuous stirring, and the pH change was monitored in real-time after each addition using a pH meter (PHB-1, shsan-xin, China).

**2.7 pH-Dependent Catalytic Hydrolysis of ATP and ADP by Ce-MOF and Ce-Ca**

To assess the influence of pH on the catalytic hydrolysis of ATP and ADP, the activities of Ce-MOF and Ce-Ca were measured across a range of buffer solutions (pH 2–10) using the malachite green assay. Reactions were conducted with Ce-MOF (25 mg·L^-1^) or Ce-Ca (100 mg·L^-1^) and 125 μM substrate (ATP or ADP). The relative catalytic activity at each pH was normalized to that under physiological conditions (pH 7.4), which was set as 100%.

**2.8 Evaluation of H_2_O_2_ Scavenging Capacity of Ce-MOF and Ce-Ca at Different pH Levels**

The H_2_O_2_ degradation activities of Ce-MOF, Ce-Ca, and Ce-Ca +H^+^ were assessed using a commercial hydrogen peroxide detection kit (Shanghai mlbio Co., Ltd.). Following the manufacturer’s instructions, H_2_O_2_ was incubated with each sample, and the reaction pH was adjusted to 5.5 or 7.4. After the reaction, the residual H_2_O_2_ was determined by measuring the absorbance at 405 nm, which reflects the formation of a chromogenic complex between H_2_O_2_ and molybdate. The amount of H_2_O_2_ consumed and remaining was calculated based on a standard curve.

**2.9 Analysis of SOD-like Activity of Ce-MOF and Ce-Ca under Different pH Conditions**

The superoxide dismutase (SOD) mimetic activity of the materials was evaluated using an SOD assay kit (Shanghai mlbio Co., Ltd.). According to the protocol, the reagents and samples were mixed and the pH was adjusted to 5.5 or 7.4. After the reaction, the absorbance at 540 nm was measured with a microplate reader.

**2.10 Preparation of dCe-Ca (Acid Etching of Ce‑Ca)**

To fully expose the inner Ce‑MOF in Ce‑Ca nanoparticles, 10 mg of Ce‑Ca was added into 1 mL of PBS at pH 3.5, and the reaction was maintained for 30 min to ensure efficient etching. The resulting dispersion was then diluted with PBS according to the actual dosage, based on the principle that the inner Ce‑MOF content was consistent with that in the Ce‑MOF group.

**3. *In Vitro* Cell Experiments**

**3.1 Cell Culture and Cytotoxicity Assessment**

J774A.1 cells (Shanghai fudancell Co., Ltd., China) were cultured in DMEM supplemented with 10% fetal bovine serum and 100 IU/mL penicillin‑streptomycin at 37 °C under 5% CO₂. Cells were passaged at 80% confluence using a cell scraper at a split ratio of 1:2 or 1:3. Cytotoxicity was evaluated using the CCK‑8 assay. Briefly, J774A.1 cells were seeded in 96‑well plates at a density of 5,000 cells per well. After adherence, cells were treated with varying concentrations of Ce‑MOF, Ce‑Ca, and Ce‑Ca +H^+^ for 24 or 48 h, followed by incubation with CCK‑8 reagent to determine cell viability.

**3.2 Cellular Uptake Study**

To evaluate the targeting effect of the materials toward M1 macrophages, cellular uptake of unmodified and HA‑modified materials by J774A.1 cells before and after polarization was examined. Leveraging the porous nature of Ce‑MOF, the small‑molecule dye Cy5 was loaded into the framework by stirring Ce‑MOF with 1 mg of Cy5 in 5 mL of water for 12 h. After centrifugation and washing, Cy5‑loaded Ce‑MOF was coated with HA following the previously described method to obtain Cy5‑labeled Ce‑HA. J774A.1 cells were seeded into glass‑bottom dishes and allowed to adhere. Where indicated, cells were polarized with 1 μg/mL LPS for 12 h. The medium was then replaced with DMEM containing 100 μg/mL of Cy5‑labeled Ce‑MOF or Ce@HA. After 2 h of incubation, cells were washed three times with PBS to remove non‑internalized nanoparticles, fixed, stained with DAPI (Beyotime Biotechnology Co., Ltd., China), and observed under a Confocal Laser Scanning Microscope (CLSM) to evaluate nanoparticle uptake.

**3.3 Suppression of M1 Macrophage Pyroptosis *In Vitro***

**3.3.1 Establishment of the M1 Macrophage Pyroptosis Model *In Vitro***

J774A.1 cells were seeded in 6-well plates at a density of 1 × 10^5^ cells per well. After overnight adherence, cells except for those in the control group, were polarized into the pro-inflammatory M1 phenotype by stimulation with 1 μg/mL LPS for 12 hours. Following a 2-hour incubation with the test materials, pyroptosis was induced by adding 5 mM ATP for 30 minutes. In this study, the dosages of each material group were strictly controlled to ensure the Ce content in the pure Ce‑MOF group was identical to that in the Ce‑Ca group. For the Ce‑Ca composite, the mass ratio between the inner Ce‑MOF core and the outer CaCO_3_ shell was approximately 1:3. The experimental groups were designated as follows: (1) Control; (2) LPS/ATP; (3) Ce-MOF (final concentration: 25 μg/mL during cell incubation); (4) Ce-Ca (final concentration: 100 μg/mL during cell incubation); (5) dCe-Ca (an equivalent amount of Ce-Ca as in group (4), treated with hydrochloric acid to etch the external calcium carbonate shell).

**3.3.2 Measurement of Extracellular and Intracellular ROS Scavenging Capacity**

J774A.1 cells were cultured and treated following the aforementioned pyroptosis induction protocol. After treatment, cells were incubated with 10 μM DCFH-DA (Beyotime Biotechnology) for 30 minutes, washed three times with PBS, and counterstained with DAPI to visualize nuclei. Cellular fluorescence was observed using confocal laser scanning microscopy.

**3.3.3 Evaluation of Cell Pyroptosis by Annexin V-FITC/PI Staining and Flow Cytometry *In Vitro***

J774A.1 cells (10^5^ cells/dish) were seeded in confocal laser scanning microscopy (CLSM) dishes. After adherence, cells were cultured and treated as described in the pyroptosis model. Staining was performed using an Annexin V-FITC Apoptosis Detection Kit (C1062L, Beyotime Biotechnology) and results were directly visualized by confocal fluorescence microscopy. Annexin V-FITC (green fluorescence) identifies apoptotic cells, while propidium iodide (PI, red fluorescence) stains necrotic or late-stage apoptotic cells.

For flow cytometric analysis of apoptosis, identically treated adherent cells were washed with PBS, detached using a trypsin-free EDTA solution, and centrifuged at 1500 × g for 5 minutes. After washing, cells were stained according to the kit protocol. Following a 30-minute incubation, stained cells were washed, resuspended in 300 μL of PBS, and analyzed using a BD flow cytometer (BD Biosciences, USA). Fluorescence signals were quantified using FlowJo software (v10.8.1), with gating strategies established based on unstained control groups.

**3.3.4 Assessment of Mitochondrial Function by Mito-Tracker Red CMXRos Staining and Flow Cytometry**

J774A.1 cells were seeded in confocal dishes at a density of 10⁵ cells/dish and subjected to the standardized pyroptosis induction protocol following adherence. Mitochondrial membrane potential was evaluated using a commercial mitochondrial membrane potential and apoptosis detection kit (C1049B, Beyotime Biotechnology), followed by direct visualization under confocal fluorescence microscopy.

For quantitative analysis, identically treated cells were detached with trypsin-free EDTA solution after PBS washing, centrifuged at 1,500 × g for 5 min, and processed according to the manufacturer's staining protocol. After 30 min incubation and subsequent washing, stained cells were resuspended in 300 μL PBS and analyzed using a BD flow cytometer (BD Biosciences, USA). Fluorescence quantification and gating were performed with FlowJo software (v10.8.1) using unstained controls as reference.

**3.3.5 Analysis of Pyroptosis-Related Gene Expression in Macrophages by Reverse Transcription Quantitative Polymerase Chain Reaction (RT-qPCR)**

The mRNA expression levels of pyroptosis-related genes (Gsdmd, Nlrp3, Pycard/ASC, Caspase-1, Il-1β, and Il-18) in M1 macrophages were determined by RT-qPCR. J774A.1 cells were seeded in 6-well plates at a density of 1 × 10⁵ cells per well. After adherence, the cells were stimulated with 1 μg/mL LPS for 12 hours. Total RNA was then extracted using Trizol reagent (Invitrogen), and its concentration was quantified using a NanoDrop 2000 spectrophotometer (Thermo Fisher Scientific Inc.). Subsequently, 500 ng of RNA was reverse-transcribed into complementary DNA (cDNA) using a PrimeScript RT reagent kit (Takara, Japan), which includes a gDNA eraser to effectively eliminate genomic DNA contamination. Quantitative PCR was performed using a SYBR Green qPCR premix kit (Bimake, USA) according to the manufacturer's instructions. The thermal cycling protocol consisted of initial denaturation at 95 °C for 30 s, followed by 40 cycles of denaturation at 95 °C for 5 s, annealing at 60 °C for 30 s, and a final extension at 72 °C for 10 minutes. Relative gene expression was calculated using the ∆∆CT method, normalizing the Ct values of the target genes to that of the housekeeping gene GAPDH.

The primer sequences employed for quantitative reverse transcription-PCR (qRT-PCR) amplification were as follows:

Gapdh:5′- TGTGTCCGTCGTGGATCTGA -3′, 5′- TTGCTGTTGAAGTCGCAGGAG -3′;

Gsdmd:5′-CCCGTTATTCATGTGTCAACCTGT-3′, 5′- GCCCTGAATGTTCCCATCGAC -3′;

Nlrp3:5′- ATTGCTGTGTGTGGGACTGAA-3′ ,5′- ATCCTGACAACACGCGGAT -3′;

Pycard:5′- CTTACAGGAGCTGGCTGAGCA -3′, 5′- GACCCTGGCAATGAGTGCTTG -3′;

Caspase-1:5′-ATTGCTTTCTGCTCTTCAACACC-3′,5′-ACCAGGCATATTCTTTCATGTGT -3′;

Il-1β:5′- TGGTGTGTGACGTTCCCATT -3′, 5′- TGTCGTTGCTTGGTTCTCCT -3′;

Il-18:5′-TGACCAAGTTCTCTTCGTTGACAA -3′, 5′- CACAGCCAGTCCTCTTACTTCAC -3′;

Nfe2l2:5′- GGACATGGAGCAAGTTTGGC -3′, 5′- TGGGAACAGCGGTAGTATCAG -3′;

Cat:5′- CCAGTGCGCTGTAGATGTGAAAC -3′, 5′- GGTGGACGTCAGTGAAATTCTTG -3′;

Sod1:5′- GAAAGCGGTGTGCGTGC -3′, 5′- ATACTGATGGACGTGGAACCC -3′;

Nos2:5′- ACTACTGCTGGTGGTGACAA -3′, 5′- GAAGGTGTGGTTGAGTTCTCTAAG -3′;

Gpx4:5′- TTACGAATCCTGGCCTTCCC -3′, 5′- TGGGCTGGACTTTCATCCATT -3′;

Nox2:5′- GGGATGAATCTCAGGCCAATCA -3′, ′- TTCAGGGCCACACAGGAAAA -3′;

Nox4:5′- CCAAATGTTGGGCGATTGTGT -3′, 5′- CAGGACTGTCCGGCACATAG -3′;

**3.3.6 Scanning Electron Microscopy (SEM) Analysis of Pyroptotic Morphology**

J774A.1 cells were seeded on glass coverslips and subjected to the standard pyroptosis induction protocol after adherence. Following treatment, the cells were washed twice with PBS and fixed with 4% paraformaldehyde for 30 minutes. Subsequently, the samples were dehydrated through a graded ethanol series, critical-point dried, and sputter-coated with a thin layer of gold at 30 mV for 75 seconds. Cellular morphology was examined using a Hitachi S-4800 scanning electron microscope (Hitachi, Tokyo, Japan).

**3.3.7 Immunofluorescence Staining of Pyroptosis Marker Proteins**

J774A.1 cells were cultured in confocal dishes and treated as per the established pyroptosis model. After treatment, cells were fixed with 4% paraformaldehyde for 30 minutes at 4 °C, washed twice with PBS, and permeabilized with 0.5% Triton X-100 for 15 minutes. Non-specific binding sites were blocked with 5% bovine serum albumin (BSA) for 60 minutes. The cells were then incubated overnight at 4 °C with primary antibodies against NLRP3(ab270449, abcam), ASC (ab309497, abcam), and Cleaved-Caspase-1(HY-P80622, medchemexpress). After washing, the samples were incubated with Cy3 conjugated goat anti-rabbit secondary antibodies (1:1000 dilution) for 60 minutes. Nuclei were counterstained with DAPI. Images were acquired using a confocal fluorescence microscope, and fluorescence intensity was quantified with ImageJ software (version 1.53e).

**3.3.8 Western Blot Analysis of Pyroptosis Marker Proteins**

J774A.1 cells were seeded in 6-well plates at a density of 1 × 10^5^ cells per well and allowed to adhere overnight. Subsequently, cells were cultured and treated according to the aforementioned pyroptosis induction protocol. After treatment, the culture medium was aspirated, and cells were gently washed twice with ice-cold PBS. Cells were then lysed directly in the culture dish using an appropriate volume of pre-chilled RIPA lysis buffer containing protease and phosphatase inhibitors, followed by incubation on ice for 30 min. The lysates were centrifuged, and the protein concentration of the supernatants was determined and normalized. Equal amounts of protein (20 µg per sample) were mixed with 5× loading buffer, denatured by heating at 95°C for 10 min in a metal bath, and subjected to SDS-PAGE. Electrophoresis was initially performed at a constant voltage of 80 V to allow sample compression within the stacking gel. Once the samples entered the separating gel, the voltage was increased to 120 V and maintained until the bromophenol blue dye reached the bottom of the gel. Proteins were then transferred onto a PVDF membrane under ice-cold conditions using a constant current of 200-350 mA for 60-120 min, with the transfer duration adjusted according to the molecular weight of the target proteins. The membrane was blocked with 5% bovine serum albumin (BSA) at room temperature to minimize non-specific binding. Subsequently, it was incubated overnight at 4°C with gentle shaking in primary antibodies-specifically targeting Caspase-1 p10(bs-20617R, BIOSS), Caspase-1 p20( bs-10743R, BIOSS), total Caspase-1(ET1608-69, HUABIO), GSDMD-FL(ab219800, abcam), GSDMD-NT(ab215203, abcam), NLRP3(ab215203, HUABIO), ASC(340097, ZENBIO), pro-IL-1β(ab216995, abcam), and IL-1β(ab283818, abcam)-diluted in blocking buffer according to the manufacturer’s recommendations. After incubation, the membrane was washed three times with 1× TBST to remove unbound antibodies and then incubated with corresponding horseradish peroxidase (HRP)-conjugated secondary antibodies for 2 h at room temperature. Prior to detection, the membrane was treated with ECL Plus reagent, and protein bands were visualized using a chemiluminescence imaging system. Semi-quantitative analysis was performed using ImageJ software to determine relative protein expression levels.

**3.3.9 Detection of Pro-inflammatory Cytokines**

J774A.1 cells were seeded in 12-well plates at 1 × 10^5^ cells per well and incubated overnight. Cells were then treated as described in the pyroptosis assay protocol. Following treatment, cell culture supernatants were collected. Standards of known concentrations and the collected supernatants were added sequentially to antibody-coated wells of a microplate and incubated at room temperature to allow capture of the target cytokines. After incubation, the plate was washed to remove unbound substances. A biotin-labeled detection antibody was then added and incubated, followed by another washing step. Streptavidin-conjugated horseradish peroxidase (HRP) was introduced, forming an immobilized “capture antibody-antigen-detection antibody-enzyme” complex. After a final wash, tetramethylbenzidine (TMB) substrate was added for color development. The reaction was terminated using a stop solution, and the absorbance of each well was immediately measured at 450 nm using a microplate reader. The concentrations of IL-18 and IL-1β in the supernatants were calculated based on a standard curve.

**3.4 *In Vitro* Repolarization Analysis of M1 Macrophages**

**3.4.1 Induction of M1 Phenotype in Macrophages *In Vitro***

To investigate whether the materials could synergistically promote the repolarization of M1 macrophages toward the M2 phenotype by accelerating ATP-adenosine conversion and restoring redox homeostasis-thereby mimicking the high ATP environment characteristic of the rheumatoid microenvironment-an *in vitro* M1 polarization model was established using J774A.1 macrophages under LPS/ATP co-stimulation. The experimental procedure was as follows:

J774A.1 cells were seeded in 6-well plates at a density of 1 × 10^5^ cells per well and allowed to adhere overnight. The control group remained unstimulated, while other groups were treated with 1 μg/mL LPS for 12 hours to induce a pro-inflammatory M1 phenotype. Subsequently, the medium was replaced with fresh medium containing 1 μg/mL LPS, 100 μM ATP, and corresponding test materials, followed by another 12 hours of incubation. The experimental groups were designed as follows:（1）Control;（2）LPS/ATP;（3）Ce-MOF (final concentration 25 μg/mL during cell incubation);（4）Ce-Ca (final concentration 100 μg/mL during cell incubation);（5）dCe-Ca (equivalent amount of Ce-Ca as in group (4), with hydrochloric acid added to etch the external calcium carbonate shell).

**3.4.2 Measurement of Adenosine Concentration by High-Performance Liquid Chromatography (HPLC)**

Following the establishment of the polarization model as described above, cell culture media were collected. An equal volume of methanol was added to the media to precipitate proteins at -20 °C for 1 h. The mixtures were then centrifuged at 12,000 × g for 15 min at 4 °C. The resulting supernatants were carefully collected and filtered through a 0.22 μm syringe filter prior to HPLC analysis.

Chromatographic separation was performed using a Beckman System Gold 125 solvent module equipped with a Beckman C18 analytical column and a Beckman 166 detector. The analytes were eluted over a 20-minute period with a linear gradient of NaH_2_PO_4_/tetrabutylammonium bromide (73.5/6 mM, pH 5.8) and methanol (25%), at a constant flow rate of 0.5 mL/min. Quantification of adenosine and its metabolites was achieved by comparing the peak areas of the samples against a calibration curve constructed from standard solutions of known concentrations.

**3.4.3 Measurement of Intracellular cAMP Levels by ELISA**

J774A.1 cells were seeded in 6-well plates at a density of 1 × 10^5^ cells per well and allowed to adhere overnight. Cells were then cultured and treated following the polarization model described previously. After treatment, the culture medium was aspirated, and cells were gently washed twice with ice-cold PBS. Cells were lysed directly in the culture wells using an appropriate volume of pre-chilled RIPA lysis buffer containing protease and phosphatase inhibitors, followed by incubation on ice for 30 min. The lysates were centrifuged, and the protein concentration of the supernatants was determined, normalized, and subsequently diluted 20-fold for analysis.

Intracellular cAMP levels were measured using a commercial ELISA kit (ELK11215, Wuhan Kelu, China) according to the manufacturer’s instructions. Briefly, after equilibrating all reagents and samples to room temperature, 100 µL of standard working buffer (gradient-diluted as instructed) or sample was added to each well and incubated at 37 °C for 80 min. The liquid was then discarded, and each well was washed three times with 200 µL of wash buffer. After blotting dry, 100 µL of biotinylated antibody working solution was added to each well and incubated at 37 °C for 50 min. Following another three washes, 100 µL of HRP enzyme working solution was added and incubated at 37 °C for 50 min. The plate was then washed five times with wash buffer, and 90 µL of TMB substrate was added to each well. After incubating at 37 °C for 20 min in the dark, the reaction was stopped by adding 50 µL of stop solution. Absorbance was immediately measured at 450 nm, and cAMP concentrations were calculated based on the standard curve.

**3.4.4 Western Blot Analysis**

J774A.1 cells were seeded in 6-well plates at a density of 1 × 10^5^ cells per well and allowed to adhere overnight. Subsequently, cells were cultured and treated following the aforementioned polarization model. Procedures for cell lysis, protein extraction, concentration measurement, protein denaturation, SDS-PAGE, membrane transfer, and blocking were performed as described in the previous Western blotting protocol (Section 4.3.8). The membranes were then incubated overnight at 4°C with gentle shaking in primary antibodies-specifically targeting p-PKA (HA721864, HUABIO), PKA (ab75991, abcam), p-CREB (ET7107-93, HUABIO), and CREB (R381013, ZENBIO)—diluted in blocking buffer according to the manufacturer's recommendations. Following primary antibody incubation, the membranes were washed three times with 1× TBST to remove unbound antibodies and subsequently incubated with appropriate HRP-conjugated secondary antibodies for 2 h at room temperature. Protein bands were visualized using ECL Plus reagent and a chemiluminescence imaging system. Semi-quantitative analysis was performed using ImageJ software to determine relative protein expression levels.

**3.4.5 Quantitative Real-Time PCR (RT-qPCR) for Macrophage Polarization Markers**

J774A.1 cells were seeded in 6-well plates at a density of 1 × 10^5^ cells per well. After overnight adherence, cells were cultured and treated according to the previously described polarization model. Total RNA extraction, mRNA concentration measurement, reverse transcription, and amplification procedures were performed as referenced in earlier sections. The primer sequences used for quantitative RT-PCR are listed below:

INOS:5′- ACTACTGCTGGTGGTGACAA - 3′、5′- GAAGGTGTGGTTGAGTTCTCTAAG -3′;

ARG1:5′- GTGCCCTCTGTCTTTTAGGGCT -3′、5′- TGCTGCGGGACCTTTCTCTA -3′;

IL-6:5′- TGATGGATGCTACCAAACTGGA -3′、5′- TCTCTCTGAAGGACTCTGGCT -3′;

TNF-α:5′- GAGCACAGAAAGCATGATCCG -3′、5′- TAGACAGAAGAGCGTGGTGG -3′;

TGF-β:5′- ATACGCCTGAGTGGCTGTCT -3′、5′- CGTGGAGTTTGTTATCTTTGCTGT -3′;

CD163:5′- TGCTCAGGAAACCAATCCCAG -3′、5′- ACCTCCACCTACCAAGCGAA -3′;

CD206:5′- AGGATCATACTTCCCTGCTTGCTA -3′、5′- CACCCTCCATCTATCCGTCCAA -3′;

CD86:5′- TCAATGGGACTGCATATCTGCC -3′、5′- GCCAAAATACTACCAGCTCACT -3′;

IL-10:5′- GCCAGAGCCACATGCTCCTA -3′、5′- GATAAGGCTTGGCAACCCAAGTAA -3′;

**3.4.6 Immunofluorescence Staining for Macrophage Polarization Markers**

J774A.1 cells were seeded in confocal dishes and allowed to adhere. Cells were then cultured and treated according to the previously described polarization model. After treatment, cells were fixed with 4% paraformaldehyde for 30 min at 4 °C, washed twice with PBS, and permeabilized with 0.5% Triton X-100 for 15 min. Non-specific binding sites were blocked by incubation with 5% bovine serum albumin (BSA) for 60 min. Cells were subsequently incubated overnight at 4 °C with primary antibodies against CD86(ab119857, abcam), CD206(ab300621, abcam), IL-10(ab9969, abcam), and TNF-α(ab183218, abcam). After washing, cells were incubated with corresponding fluorescently labeled secondary antibodies for 60 min at room temperature. Nuclei were counterstained with DAPI. Images were acquired using a confocal fluorescence microscope, and fluorescence intensity was quantified using ImageJ software (version 1.53e).

**3.4.7 Flow Cytometric Analysis of Macrophage Repolarization**

J774A.1 cells were seeded in culture dishes and treated following the established polarization protocol. Cells were then harvested, washed with PBS, and incubated for 1 hour at 4 °C in the dark with the following fluorescently conjugated antibodies: FITC-labeled anti-mouse CD86 (105005, Biolegend) and APC-labeled anti-mouse CD206 (141707, Biolegend). After staining, cells were washed and resuspended in 300 μL of PBS for analysis. Flow cytometry was performed to detect FITC fluorescence (indicative of M1 polarization) and APC fluorescence (indicative of M2 polarization). Data were analyzed using FlowJo software, with gating strategies based on unstained control cells.

**3.4.8 Detection of Pro- and Anti-inflammatory Cytokines**

J774A.1 cells were seeded and subjected to the polarization protocol as described above. Following incubation, cell culture supernatants were collected. The concentrations of IL-10, TGF-β, TNF-α, and IL-6 in the supernatants were measured using respective commercial ELISA kits, according to the manufacturers' instructions.

**3.5 RNA Sequencing and Bioinformatic Analysis**

J774A.1 cells were cultured and treated following the pyroptosis induction protocol described previously. Cells were then harvested by scraping, washed with PBS, and pelleted by centrifugation. Cell pellets were lysed in TRIzol reagent (1 mL per 5 × 10^6^ cells) for total RNA isolation. The resulting RNA extracts were subsequently sent to Majorbio Biopharm Technology Co., Ltd. (Shanghai, China) for RNA-seq library preparation.

Fragmented mRNA was used as the template to synthesize first-strand complementary DNA (cDNA) using reverse transcriptase. Differential gene expression analysis between comparison groups was performed using the DESeq2 software package. Genes/transcripts with an adjusted p-value of less than 0.05 and an absolute fold change ≥ 2.0 were defined as differentially expressed. For functional enrichment analysis, Gene Set Enrichment Analysis (GSEA), as well as the Kyoto Encyclopedia of Genes and Genomes (KEGG) and Gene Ontology (GO) databases, were utilized.

**3.6 *In Vitro* Osteogenic Differentiation Assessment**

**3.6.1 Induction of Osteogenic Differentiation *In Vitro***

rBMSCs were seeded in 12-well plates at a density of 2 × 10^5^ cells per well and cultured in basal medium for 24 h. The medium was then replaced with osteogenic induction medium, which contained the respective test materials and 400 μM H_2_O_2_. The osteogenic medium was composed of 50 ng/mL ascorbic acid, 10 mM β-glycerophosphate, and 10 nM dexamethasone. The experimental groups were designed as follows: (1) Control; (2) H_2_O_2_; (3) Ce-MOF (final concentration 25 μg/mL); (4) Ce-Ca (final concentration 100 μg/mL); (5) dCe-Ca (an equivalent amount of Ce-Ca as in group (4), with hydrochloric acid added to etch the external calcium carbonate shell). The induction medium was refreshed every 2–3 days.

**3.6.2 Quantitative Assay, Staining of Alkaline Phosphatase (ALP), and Alizarin Red S (ARS) Staining**

Rat bone marrow‑derived mesenchymal stem cells (rBMSCs) were cultured according to the aforementioned protocol. After 7 days of osteogenic induction, the cells were lysed for quantitative measurement of ALP activity following the manufacturer’s instructions, and the results were normalized to the total protein content in each group. For ALP staining, cells were fixed and stained with ALP staining solution according to the kit instructions. To evaluate mineralized matrix deposition, cells were fixed and stained with Alizarin Red S (ARS) solution after 14 days of induction. Stained samples were imaged using a stereomicroscope, and the positively stained areas were quantified using ImageJ software.

**3.6.3 Analysis of Osteogenic Gene Expression by Quantitative Real-Time PCR (RT-qPCR)**

rBMSCs were seeded in culture dishes and induced under the previously described conditions. Cells were harvested at days 7 and 14 for RNA extraction. Procedures for total RNA isolation, concentration measurement, reverse transcription, and amplification were performed as referenced in earlier sections. The mRNA expression levels of key osteogenic markers, including OPN, OCN, ALP, and RUNX-2, were determined by RT-qPCR. The primer sequences used for quantitative RT-PCR are listed below:

Runx2:5′- CCATCCATCCACTCCACCAC -3′, 5′- GCCAGAGGCAGAAGTCAGAG -3′;

Alp:5′- CCTGCCTTACCAACTCTTTTGTG -3′, 5′- CATTGGTGTTGAGCTTTTGGAGT -3′;

OCN:5′- CGCTACCTTGGAGCCTCAGT -3′, 5′- AGGCGGTCTTCAAGCCATACT -3′;

OPN:5′- TACGACCATGAGATTGGCAGTGA-3′,5′-TATAGGATCTGGGTGCAGGCTGTAA -3′;

**3.6.4 Immunofluorescence Staining for Osteogenic Marker OPN**

rBMSCs were seeded in confocal dishes and cultured under the aforementioned osteogenic induction conditions. Following the induction period, cells were fixed with 4% paraformaldehyde for 30 min at 4 °C, washed twice with PBS, and permeabilized with 0.5% Triton X-100 for 15 min. Non-specific binding sites were blocked by incubation with 5% bovine serum albumin (BSA) for 60 min. Cells were then incubated overnight at 4 °C with a primary antibody against OPN (A1361, abclonal). After washing, the cells were incubated with an appropriate fluorescently conjugated secondary antibody for 60 min at room temperature. Nuclei were counterstained with DAPI. Images were acquired using a confocal fluorescence microscope, and the fluorescence intensity of OPN was quantified using ImageJ software (version 1.53e).

**4. *In Vivo* Therapeutic Evaluation in an Animal Model**

**4.1 Establishment of the AIA Mouse Model**

Male C57BL/6 mice, obtained from Chengdu Dashuo Experimental Animal Co., Ltd. (Chengdu, China), were housed under specific pathogen-free (SPF) conditions in the Animal Laboratory of West China School of Basic Medical Sciences & Forensic Medicine, Sichuan University. All animal experiments were conducted in strict accordance with the National Institutes of Health (NIH) Guide for the Care and Use of Laboratory Animals.

Arthritis was induced using complete Freund's adjuvant (CFA). Approximately 50 μL of CFA was administered via intradermal injection into the left hind paw. Mice were then randomly assigned into five experimental groups (n = 5 per group): (1) Blank control (naïve); (2) Saline-treated AIA; (3) AIA + Dexamethasone (Dex, 1mg/kg); (4) AIA + Ce-MOF (5 mg/kg); (5) AIA + Ce-Ca (20 mg/kg). By day 14 post-induction, mice developed characteristic arthritic signs in the injected paw, including erythema, swelling, and impaired mobility. The paw thickness and ankle circumference were measured every two days. The same anatomical point was marked on each mouse's ankle for consistent measurements.

Clinical arthritis scores were assessed on a scale of 0 to 5 as follows:
0: Normal; 1: Mild, diffuse erythema and/or swelling of the ankle or wrist; 2: Mild to moderate erythema and swelling of the ankle or wrist; 3: Moderate erythema and swelling of the ankle or wrist; 4: Severe erythema and swelling encompassing the entire paw; 5: Maximal inflammation of the entire limb, accompanied by significant joint stiffness and loss of function.

**4.2 Micro-CT Analysis**

Following the treatment period, mice from the different groups were subjected to whole-body scanning using a live-animal micro-CT system under isoflurane anesthesia. The acquired scans of the hind paws were reconstructed into three-dimensional (3D) structures using a filtered back-projection algorithm. The following 3D morphometric parameters were quantified for the hind paw analysis: Bone Volume (BV), Tissue Volume (TV), Trabecular Thickness ([Tb.Th](https://tb.th/)), and Trabecular Separation (Tb.Sp).

**4.3 Histological Staining (Safranin O, TRAP, and H&E)**

After euthanasia, the right hind paws were collected and skinned. All specimens were fixed in 4% formaldehyde for 48 h and subsequently decalcified in an ethylenediaminetetraacetic acid (EDTA) solution for 4 weeks. The joint tissues were then embedded in paraffin and sectioned into 5 μm-thick slices for histological staining, including Safranin O, Tartrate-Resistant Acid Phosphatase (TRAP), and Hematoxylin and Eosin (H&E).

**4.4 Detection of Intra-articular pH**

Semi-quantitative detection of local intra-articular pH was performed using a FITC-labeled dextran pH probe. First, the fluorescence intensity of the probe was measured in PBS solutions with a series of pH gradients, and a standard calibration curve was established between fluorescence signal and pH value. The fluorescence intensity detected in the joints of normal mice was used as the reference value corresponding to pH 7.4. Mice were divided into the sham group and the adjuvant-induced arthritis (AIA) group. A self-controlled design was applied for each mouse: the right hind paw was injected with a mixture of Ce-Ca nano-immunomodulator and FITC-labeled dextran probe, while the left hind paw received an equal volume of PBS mixed with the same probe. Fluorescence images of bilateral joints were captured using an in vivo fluorescence imaging system. The average fluorescence intensity of the joint region was quantified and converted to the corresponding semi-quantitative pH value based on the pre-established standard curve and the reference intensity at pH 7.4 from normal mice.

**4.5 In Vivo Metabolism and Clearance of Ce-Ca Nanoparticles**

**4.5.1 Local Retention and Clearance at the Injection Site**

To investigate the local retention and clearance of Ce-Ca nanoparticles (labeled with ICG via adsorption into the porous structure of Ce-MOF before CaCO_3_ coating) after intra-articular injection, in vivo fluorescence imaging was performed at predetermined time points (3, 6, 24, 48, 72, and 168 h post-injection). Mice were anesthetized, and fluorescence images of the knee joints were acquired using an in vivo fluorescence imaging system. The fluorescence intensity at the injection site was quantified using dedicated imaging analysis software to monitor the temporal decay of the signal, reflecting the gradual clearance of nanoparticles from the joint cavity.

**4.5.2 Biodistribution in Major Organs**

For ex vivo biodistribution analysis, mice were euthanized at 12, 48, 72, and 168 h post-injection (n = 3 per time point). Major organs including the heart, liver, spleen, lungs, kidneys, intestine, and the injected knee joint were carefully harvested and rinsed with PBS. Fluorescence images of these isolated tissues were immediately acquired to visualize the distribution of Ce-Ca nanoparticles. The fluorescence intensity of each organ was quantified to assess the temporal changes in nanoparticle accumulation and clearance.

**4.5.3 Quantitative Analysis of Ce Element by ICP-MS**

To provide definitive quantitative evidence for the long-term clearance of Ce-Ca nanoparticles, the concentration of Ce element in major organs was measured using inductively coupled plasma mass spectrometry (ICP-MS). Briefly, at 12, 48, and 168 h post-injection, organs were harvested, weighed, and digested in a mixture of concentrated nitric acid and hydrogen peroxide using a microwave digestion system. After complete digestion, the samples were diluted, and the Ce content was quantified by ICP-MS. The Ce concentration in each organ was normalized to the tissue weight, and the results were compared with those from control mice to confirm the gradual clearance of the nanoparticles.

**4.6 Immunohistochemical Staining**

Ankle joint sections were subjected to immunohistochemical analysis using specific primary antibodies against RUNX2, IL-10, TGF-β, IL-18, and IL-1β. Stained sections were visualized and imaged using a digital microscope (Olympus CX31, Japan).

**4.7 Immunofluorescence Staining**

Joint tissue sections were subjected to immunofluorescence staining to evaluate the inhibitory effect of the materials on macrophage pyroptosis using antibodies against CD68, NLRP3, and Cleaved-Caspase-1. Additionally, macrophage phenotypic polarization was assessed by co-staining with antibodies for CD68, CD163 (M2 marker), and iNOS (M1 marker).

Antigen retrieval was performed by treating the sections with citrate buffer at 95°C for 10 min. Endogenous peroxidases were then quenched by incubation with 3% hydrogen peroxide for 10 min. After blocking with 5% BSA for 60 min, the sections were incubated overnight at 4°C with the respective primary antibodies diluted in blocking buffer. Following primary antibody incubation, the sections were treated with fluorophore-conjugated secondary antibodies (diluted 1:500) for 2 h at room temperature. Images were acquired using a fluorescence microscope.

**4.8 Biosafety Evaluation**

**4.8.1 Acute Toxicity Assessment**

For acute toxicity evaluation, mice were intravenously administered Ce-Ca NPs at doses of 10, 20, and 40 mg/kg (n = 3 per group). On day 7 post-administration, body weights were recorded, and animals were euthanized for sample collection. Blood samples were collected from the abdominal aorta, and hematological parameters were analyzed using an automated hematology analyzer, including white blood cell (WBC) counts and differential counts (lymphocytes, monocytes, and segmented neutrophils), as well as red blood cell (RBC) counts, hemoglobin (Hb), mean corpuscular volume (MCV), hematocrit (HCT), and mean corpuscular hemoglobin (MCH). Serum biochemical parameters were also assessed, including alanine aminotransferase (ALT), aspartate aminotransferase (AST), alkaline phosphatase (ALP), creatinine (CREA), blood urea nitrogen (BUN), and gamma-glutamyl transferase (GGT). Major organs (heart, liver, spleen, lung, kidney, and intestine) were harvested and weighed to calculate organ-to-body weight ratios. Organ tissues were fixed, sectioned, and stained with hematoxylin and eosin (H&E) for histopathological examination.

**4.8.2 Long-term Biocompatibility Assessment**

On day 21 of the treatment period, animals were euthanized, and blood samples were collected from the abdominal aorta (n = 3 per group). A 2 mL aliquot of whole blood from each mouse was analyzed using an automated hematology analyzer. Another 3 mL of blood was centrifuged at 1000 × g for 10 min at 4°C to obtain plasma. The levels of TNF-α, IL-6, IL-1β, IL-18, IL-10, and TGF-β were measured using commercial ELISA kits. Hematological and biochemical parameters were also assessed, including alkaline phosphatase (ALP), alanine aminotransferase (ALT), aspartate aminotransferase (AST), blood urea nitrogen (BUN), and creatinine (Cr). Major organs (heart, liver, spleen, lung, and kidney) were harvested, fixed, sectioned, and stained with H&E for histopathological examination.

For the hemolysis assay, a 4% (v/v) suspension of red blood cells (RBCs) in PBS was incubated with Ce-Ca NPs at concentrations of 0, 25, 50, 100, or 200 μg/mL for 4 h at 37°C. After centrifugation at 1000 × g for 5 min at 4°C, the absorbance of the supernatant was measured at 562 nm using a microplate reader. The hemolysis percentage was calculated as follows:
Hemolysis (%) = (A_sample_ - A_negative_) / (A_positive_ - A_negative_) × 100%
where A_sample_ represents the absorbance of the supernatant from RBCs treated with Ce-Ca NPs, A_negative_ represents the absorbance from RBCs in PBS, and A_positive_ represents the absorbance from RBCs in pure water.

**5. Statistical Analysis**

All statistical analyses were performed using SPSS software. Data are presented as the mean ± standard deviation (s.d.) from at least three independent experiments (n ≥ 3). For comparisons between multiple groups, one-way analysis of variance (ANOVA) was applied, followed by Tukey’s post hoc test. A p-value of less than 0.05 was considered statistically significant.

**Supplementary Figures**

**
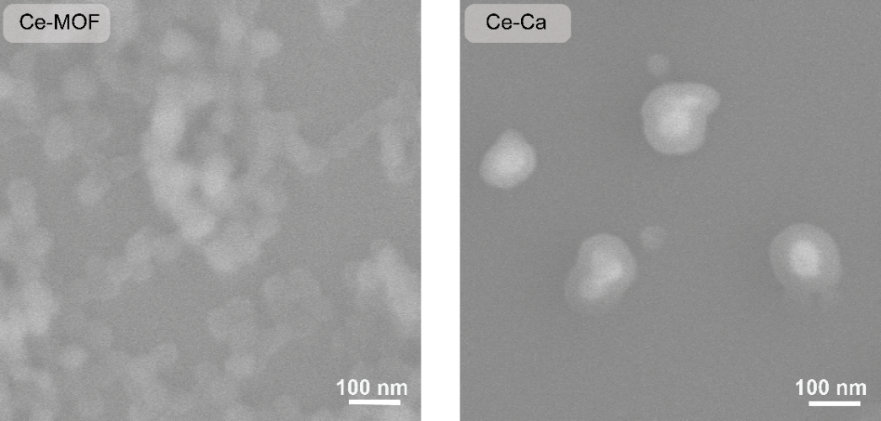
**

Figure S1 SEM image of Ce-MOF, Ce-Ca. Scale bar: 100 nm


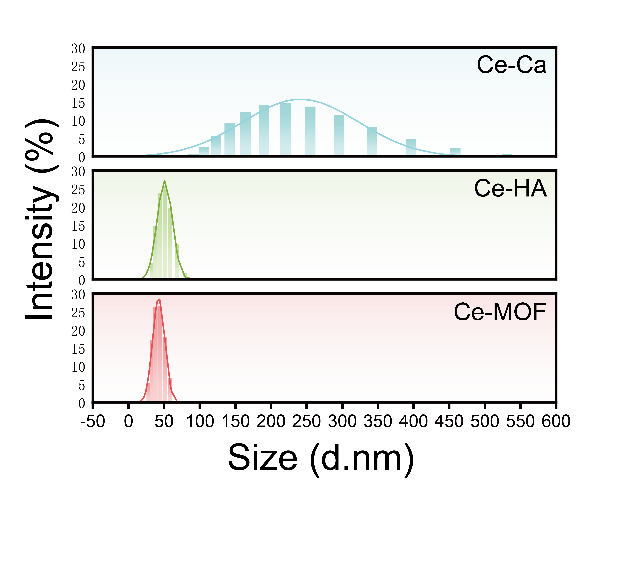


Figure S2 Particle size distributions of Ce-MOF, Ce-HA and Ce-Ca.


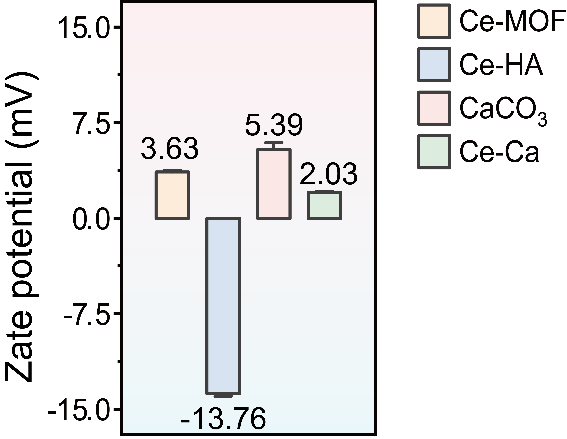


Figure S3. Zate potential of Ce-MOF, Ce-HA, CaCO3 and Ce-Ca. Data are presented as mean ± SD, n = 3. Differences were assessed by one-way analysis of variance (ANOVA) followed by Tukey’s multiple comparison test. ****P < 0.0001, ***P < 0.001, **P < 0.01 and *P < 0.05.


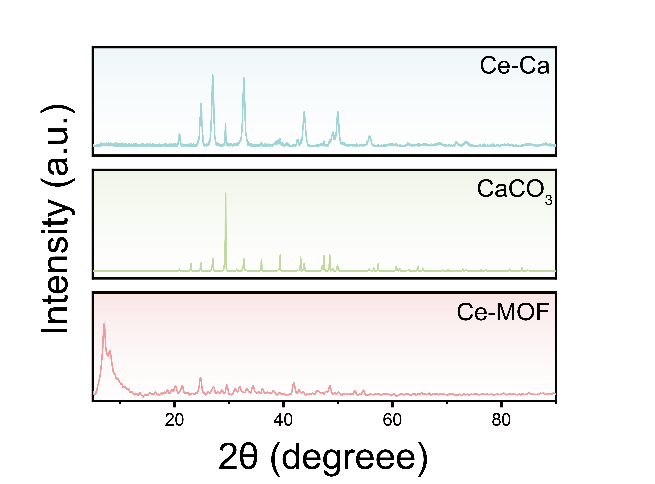


Figure S4 XRD characterization of Ce-MOF, Ce-HA and Ce-Ca.


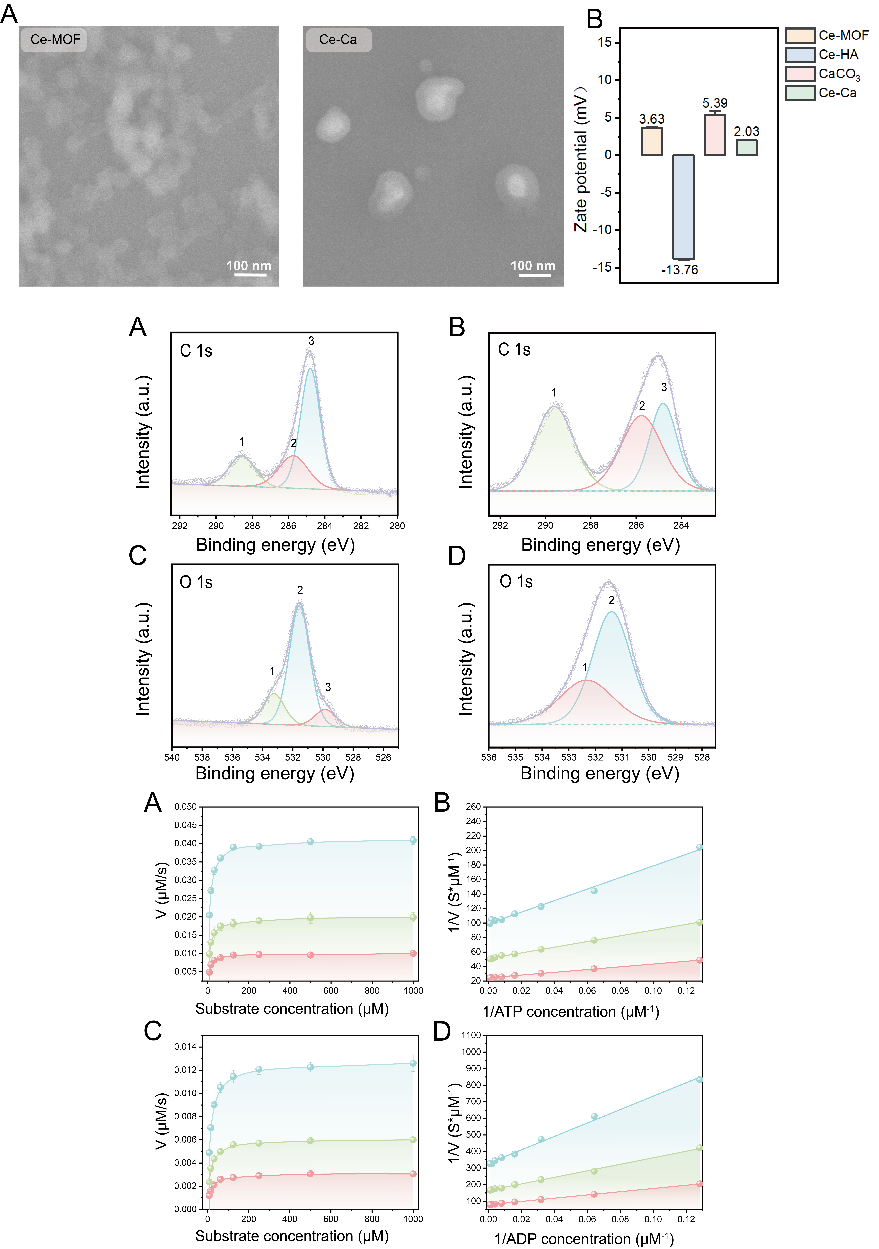


Figure S5 A) XPS analysis of the C 1s spectra of Ce-MOF, B) C 1s spectra of and Ce-Ca,C) O 1s spectra of Ce-MOF, D) O 1s spectra of Ce-Ce-Ca.


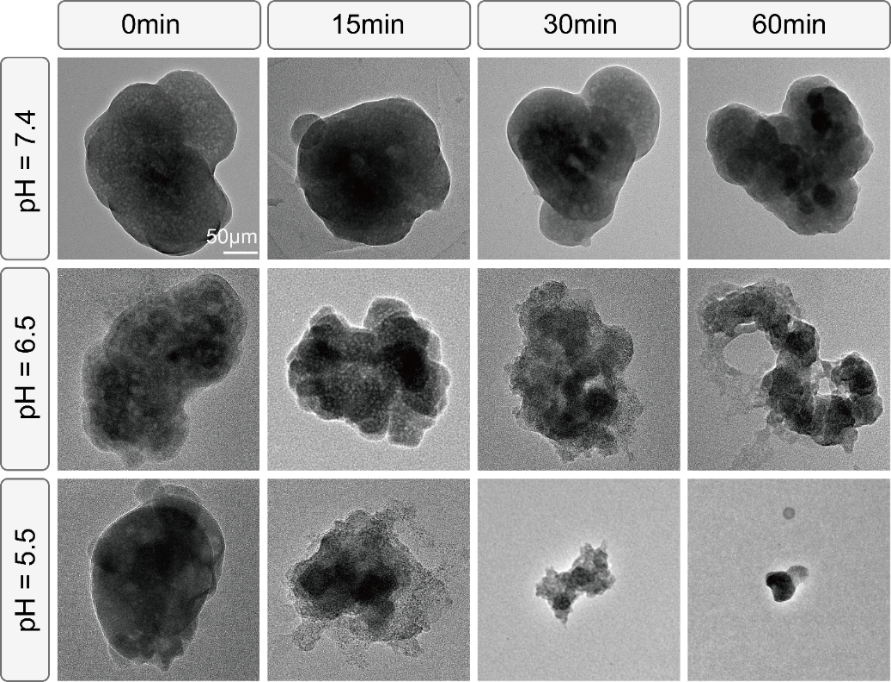


Figure S6 TEM images of Ce- Ca in PBS solutions at pH 5.5, 6.5, and 7.4 at different time points.


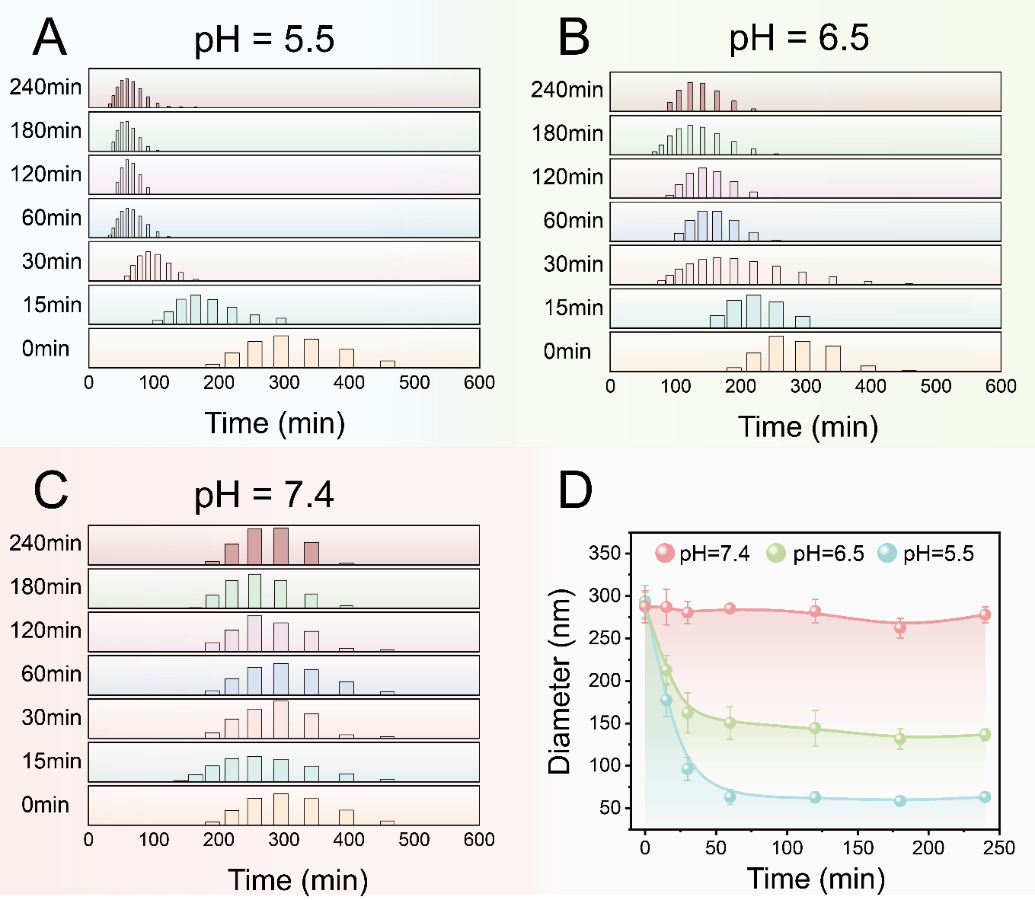


Figure S7 DLS particle size distribution histograms of Ce-Ca after incubation in PBS at A) pH 5.5, B) pH 6.5and C) pH 7.4 at different time points, D) Summary curves of the temporal evolution of the average particle diameter of Ce- Ca under the three pH conditions. Data are presented as mean ± SD, n = 3. Differences were assessed by one-way analysis of variance (ANOVA) followed by Tukey’s multiple comparison test. ****P < 0.0001, ***P < 0.001, **P < 0.01 and *P < 0.05.


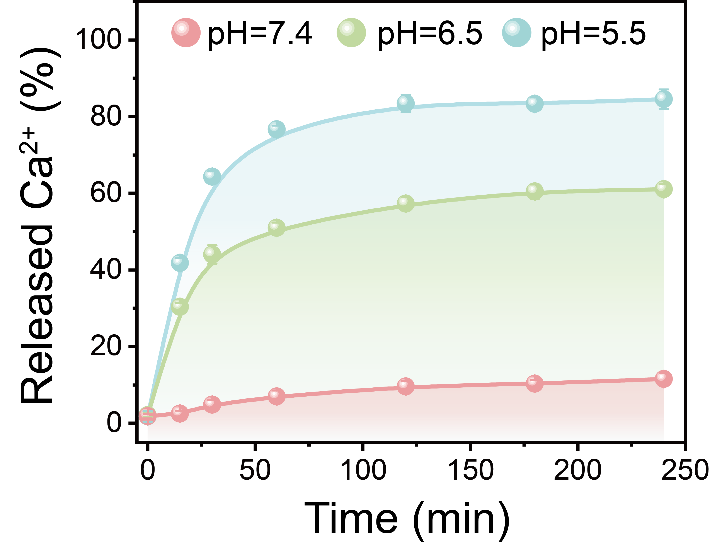


Figure S8 Time-dependent Ca^2+^ release profiles of Ce-Ca in PBS at pH 5.5, 6.5, and 7.4. Data are presented as mean ± SD, n = 3. Differences were assessed by one-way analysis of variance (ANOVA) followed by Tukey’s multiple comparison test. ****P < 0.0001, ***P < 0.001, **P < 0.01 and *P < 0.05.


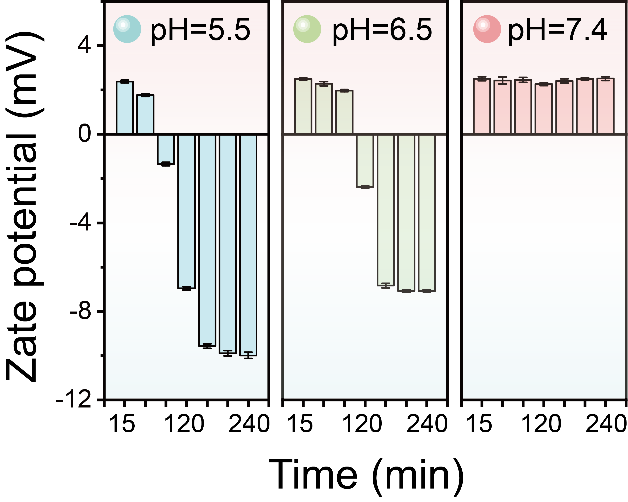


Figure S9 Time-dependent Zeta potential of Ce-Ca in PBS at pH 5.5, 6.5, and 7.4 for different time periods. Data are presented as mean ± SD, n = 3. Differences were assessed by one-way analysis of variance (ANOVA) followed by Tukey’s multiple comparison test. ****P < 0.0001, ***P < 0.001, **P < 0.01 and *P < 0.05.


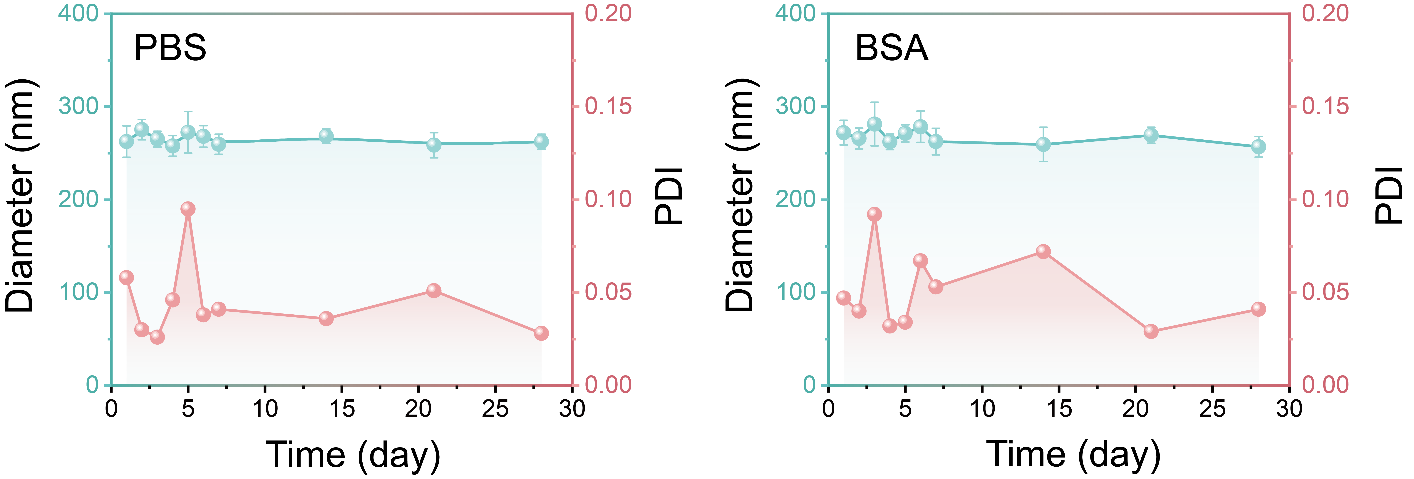


Figure S10 Time-dependent changes in particle diameter (blue) and polydispersity index (PDI, red) of Ce-Ca in PBS (left) and BSA (right) solutions over 30 days. Data are presented as mean ± SD, n = 3. Differences were assessed by one-way analysis of variance (ANOVA) followed by Tukey’s multiple comparison test. ****P < 0.0001, ***P < 0.001, **P < 0.01 and *P < 0.05.


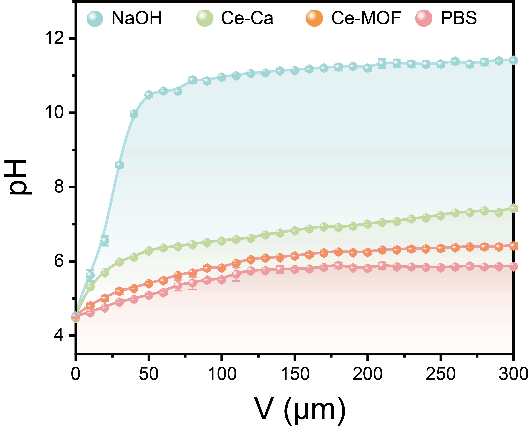


Figure S11 pH value variation curves during titrations of NaOH (0.01 M), PBS, Ce-MOF or Ce-Ca into HCl (pH = 4.5). Data are presented as mean ± SD, n = 3. Differences were assessed by one-way analysis of variance (ANOVA) followed by Tukey’s multiple comparison test. ****P < 0.0001, ***P < 0.001, **P < 0.01 and *P < 0.05.


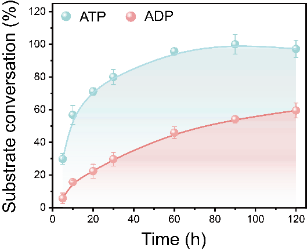


Figure S12 Substrate conversion versus time catalyzed by the Ce-MOF. Data are presented as mean ± SD, n = 3. Differences were assessed by one-way analysis of variance (ANOVA) followed by Tukey’s multiple comparison test. ****P < 0.0001, ***P < 0.001, **P < 0.01 and *P < 0.05.


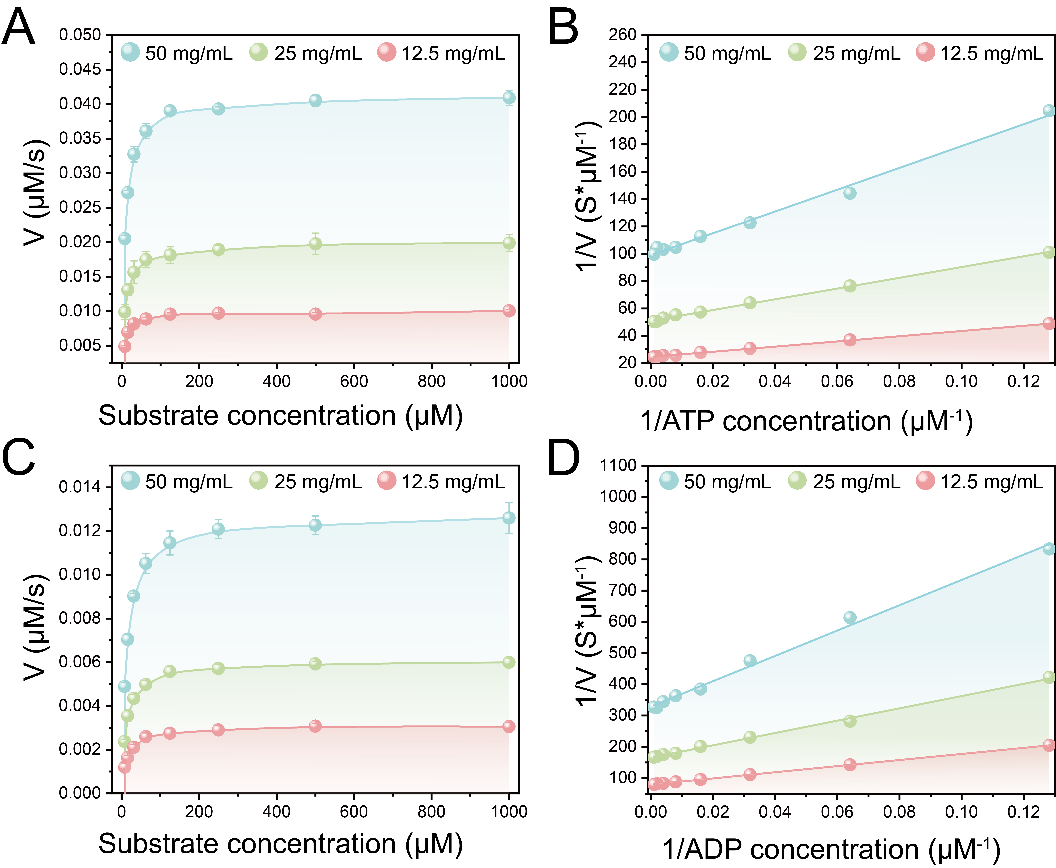


Figure S13 A) The Michaelis-Menten plots and B) the corresponding double-reciprocal fitting plots for Ce-MOF (12.5, 25 and 50 mg L^-1^) with various concentration of ATP, C) The Michaelis-Menten plots and D) the corresponding double-reciprocal fitting plots for Ce-MOF (12.5, 25 and 50 mg L^-1^) with various concentration of ADP. Data are presented as mean ± SD, n = 3. Differences were assessed by one-way analysis of variance (ANOVA) followed by Tukey’s multiple comparison test. ****P < 0.0001, ***P < 0.001, **P < 0.01 and *P < 0.05.


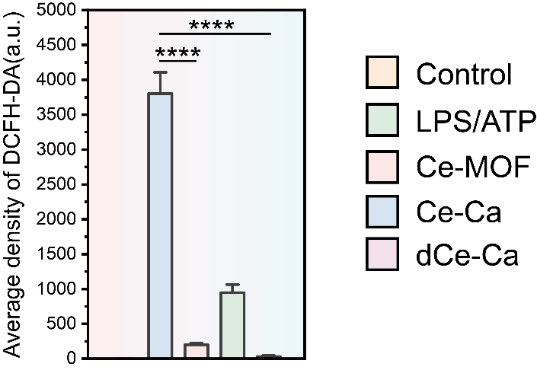


Figure S14 Semi-quantitative results of DCFH-DA staining across treatment groups. Data are presented as mean ± SD. Differences were assessed by one-way analysis of variance (ANOVA) followed by Tukey’s multiple comparison test. ****P < 0.0001, ***P < 0.001, **P < 0.01 and *P < 0.05.


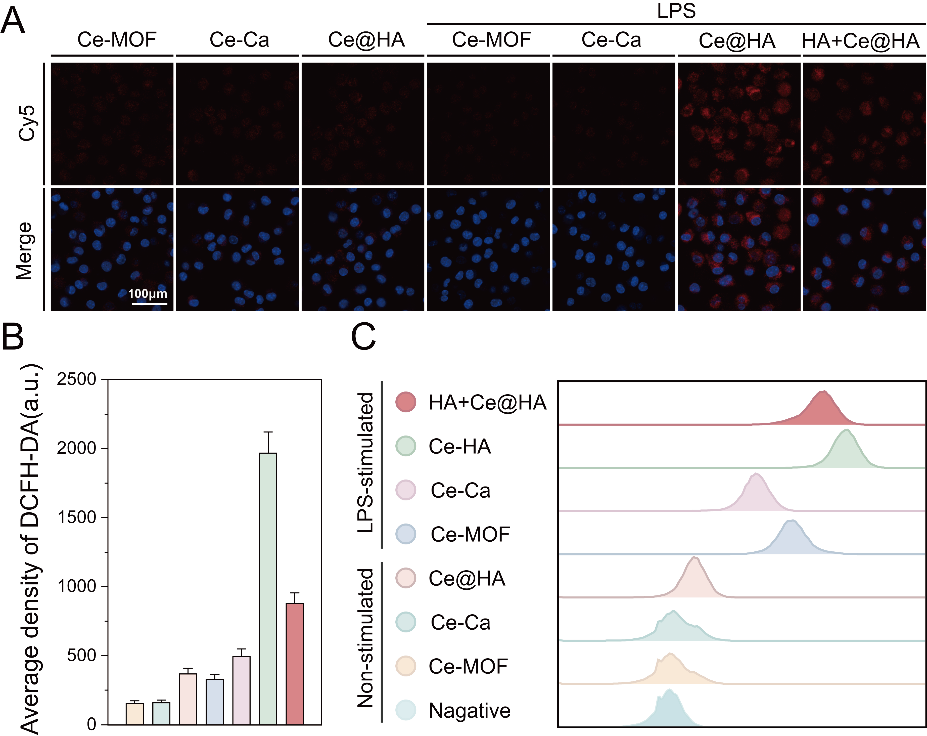


Figure S15 Evaluation of cellular uptake for different material formulations. (A) Confocal laser scanning microscopy (CLSM) images visualizing the internalization of materials (scale bar: 100 μm). (B) Semi-quantification of intracellular fluorescence based on the analysis of confocal images. (C) Quantitative assessment of uptake efficiency by flow cytometry. Data are presented as mean ± SD, n = 3. Differences were assessed by one-way analysis of variance (ANOVA) followed by Tukey’s multiple comparison test. ****P < 0.0001, ***P < 0.001, **P < 0.01 and *P < 0.05.


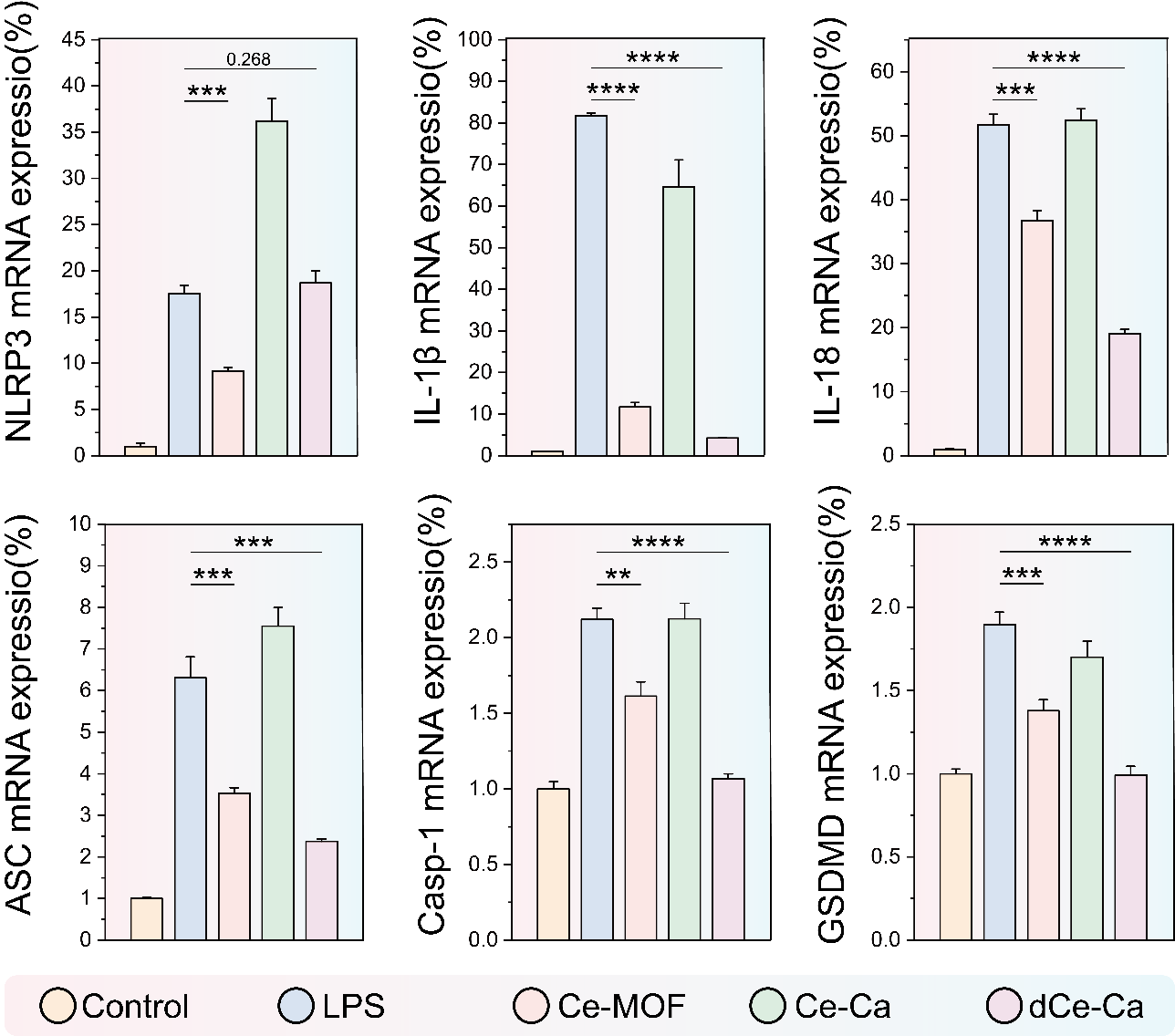


Figure S16 Quantitative real-time PCR analysis of NLRP3, IL-1β, IL-18, ASC, Caspase1, GSDMD gene expression in different groups. Data are presented as mean ± SD, n = 3. Differences were assessed by one-way analysis of variance (ANOVA) followed by Tukey’s multiple comparison test. ****P < 0.0001, ***P < 0.001, **P < 0.01 and *P < 0.05.


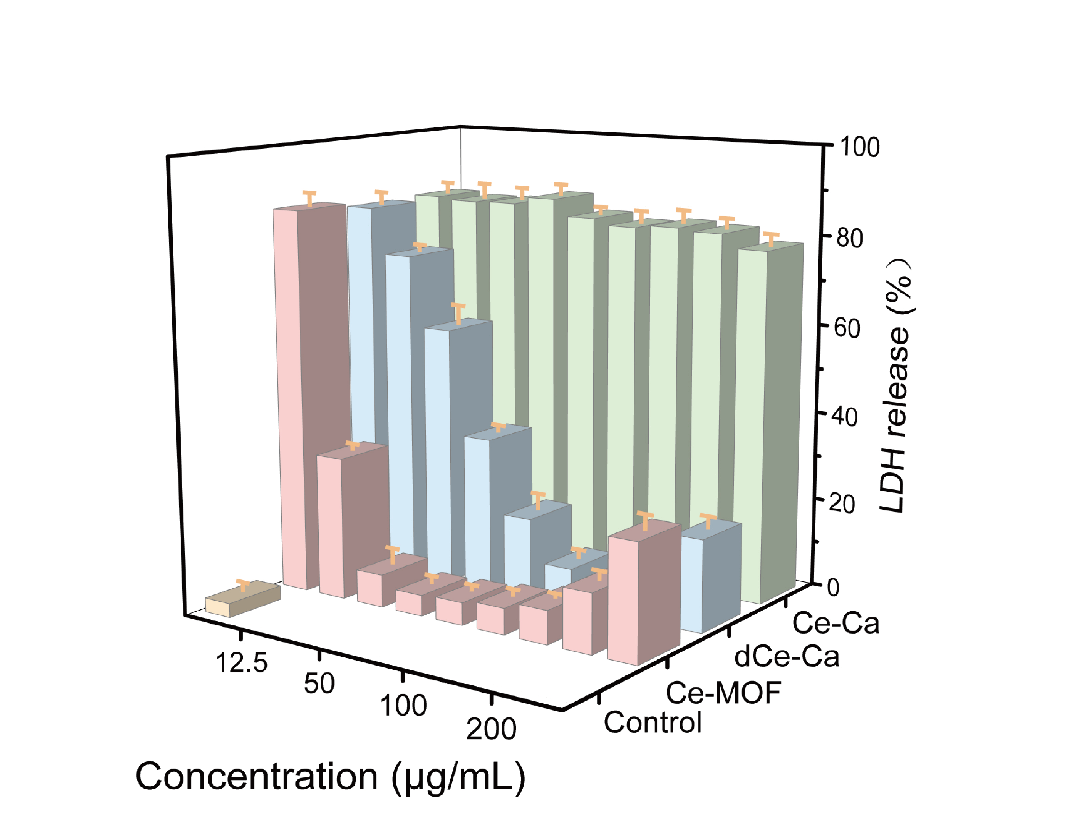


Figure S17 Lactate dehydrogenase (LDH) release from cells after treatment with different concentrations (0, 12.5, 25, 50, 75, 100, 150, 200, 400 μg/mL) of Ce-MOF, Ce-Ca, dCe-Ca. Data are presented as mean ± SD, n = 3. Data are presented as mean ± SD, n = 3. Differences were assessed by one-way analysis of variance (ANOVA) followed by Tukey’s multiple comparison test. ****P < 0.0001, ***P < 0.001, **P < 0.01 and *P < 0.05.


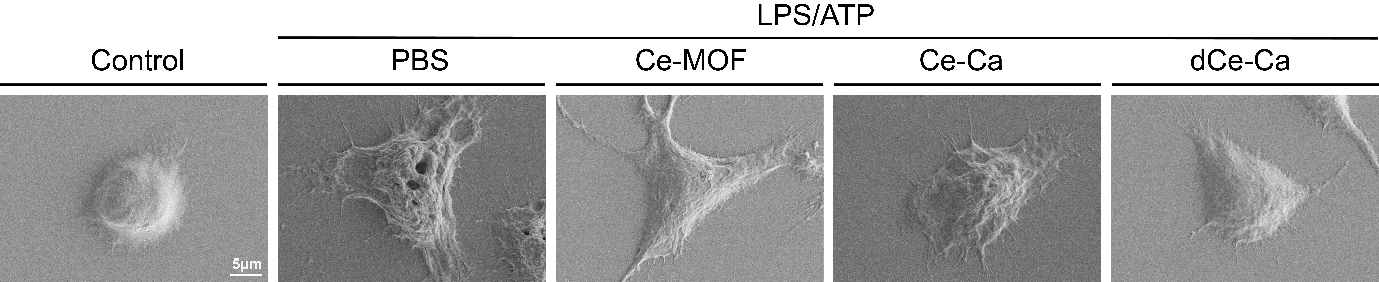


Figure S18 SEM images of J774A.1 cell in different groups.


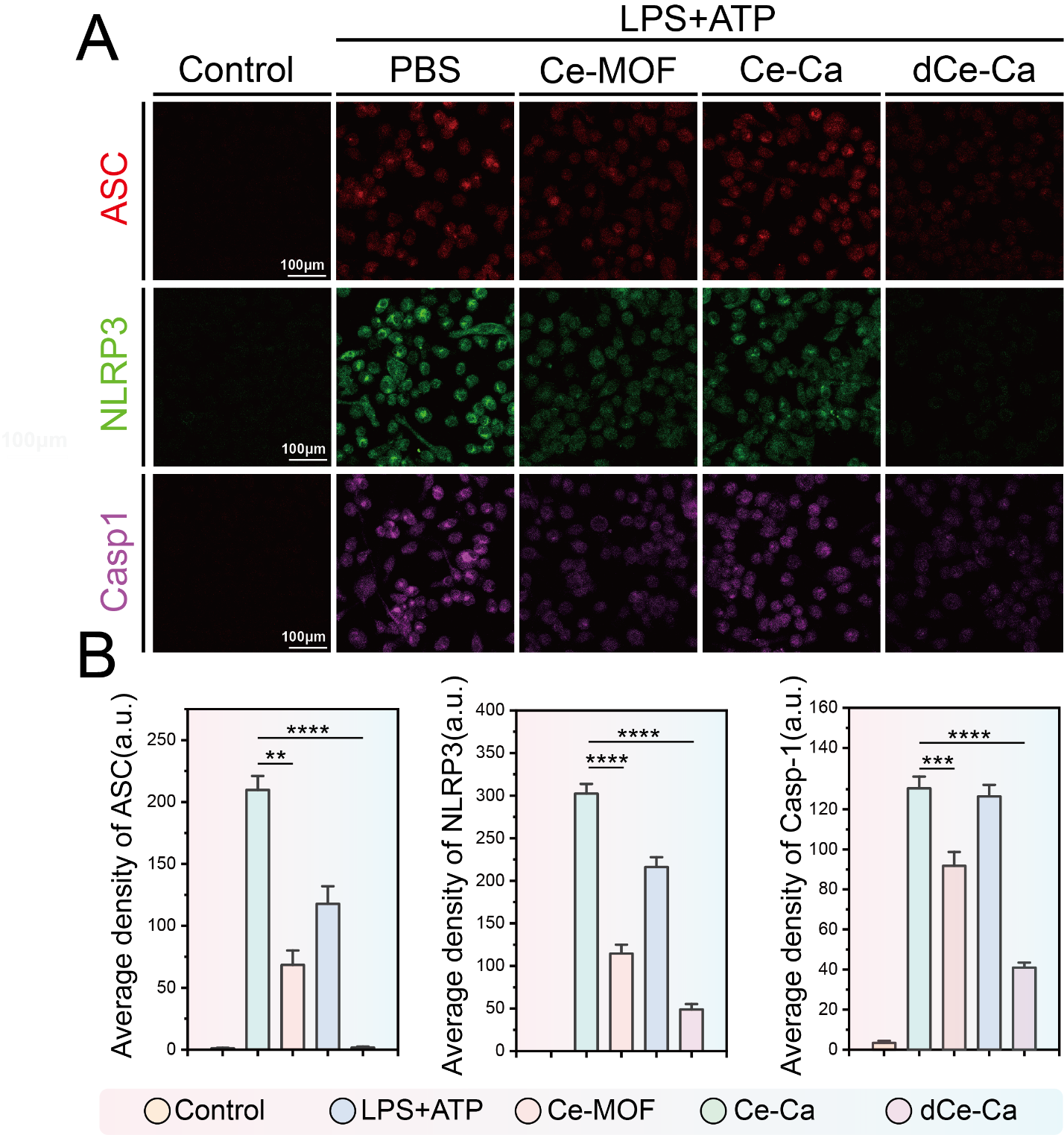


Figure S19 Immunofluorescence staining of NLRP3, ASC, GSDMD in different groups and I) Semi-quantitative analysis of fluorescence intensity. Data are presented as mean ± SD, n = 3. Differences were assessed by one-way analysis of variance (ANOVA) followed by Tukey’s multiple comparison test. ****P < 0.0001, ***P < 0.001, **P < 0.01 and *P < 0.05.


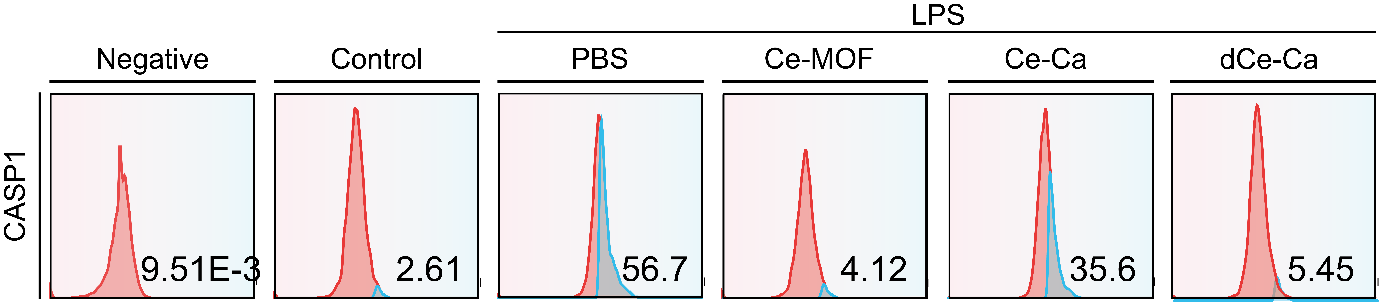


Figure S20 Flow cytometric analysis of Cleaved-Caspase1 expression in different groups.


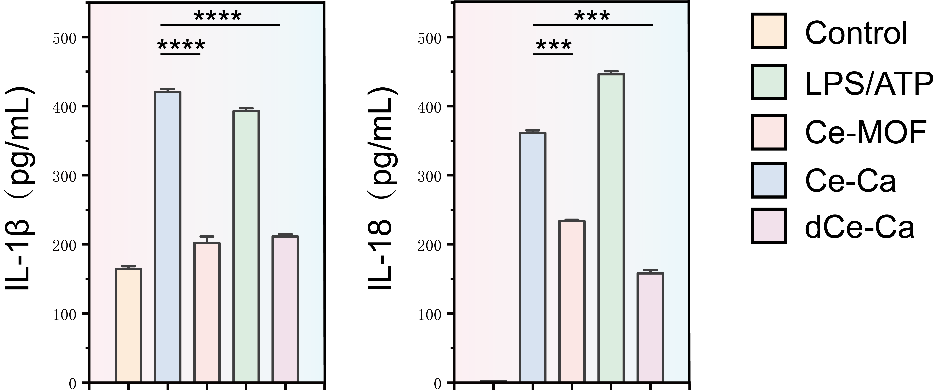


Figure S21 ELISA measurement of IL-1β, IL-18 levels in the supernatant. Data are presented as mean ± SD, n = 3. Differences were assessed by one-way analysis of variance (ANOVA) followed by Tukey’s multiple comparison test. ****P < 0.0001, ***P < 0.001, **P < 0.01 and *P < 0.05.


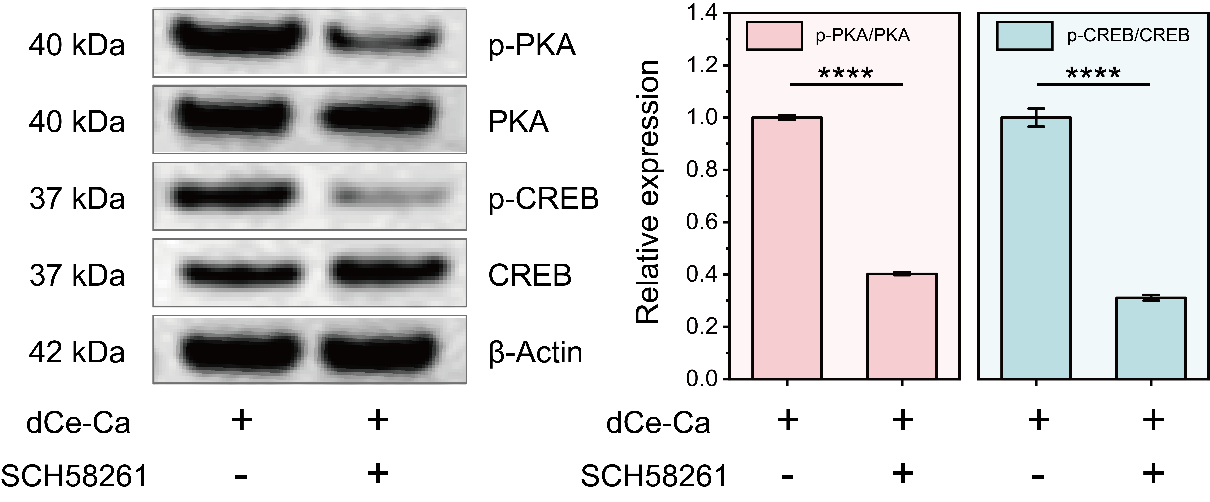


Figure S22 Western blot (WB) results and semi-quantitative analysis of PKA, CREB, p-PKA and p-CREB expression from J774A.1 cells after treatment with dCe-Ca and dCe-Ca+SCH58261. Data are presented as mean ± SD, n = 3. Differences were assessed by one-way analysis of variance (ANOVA) followed by Tukey’s multiple comparison test. ****P < 0.0001, ***P < 0.001, **P < 0.01 and *P < 0.05.


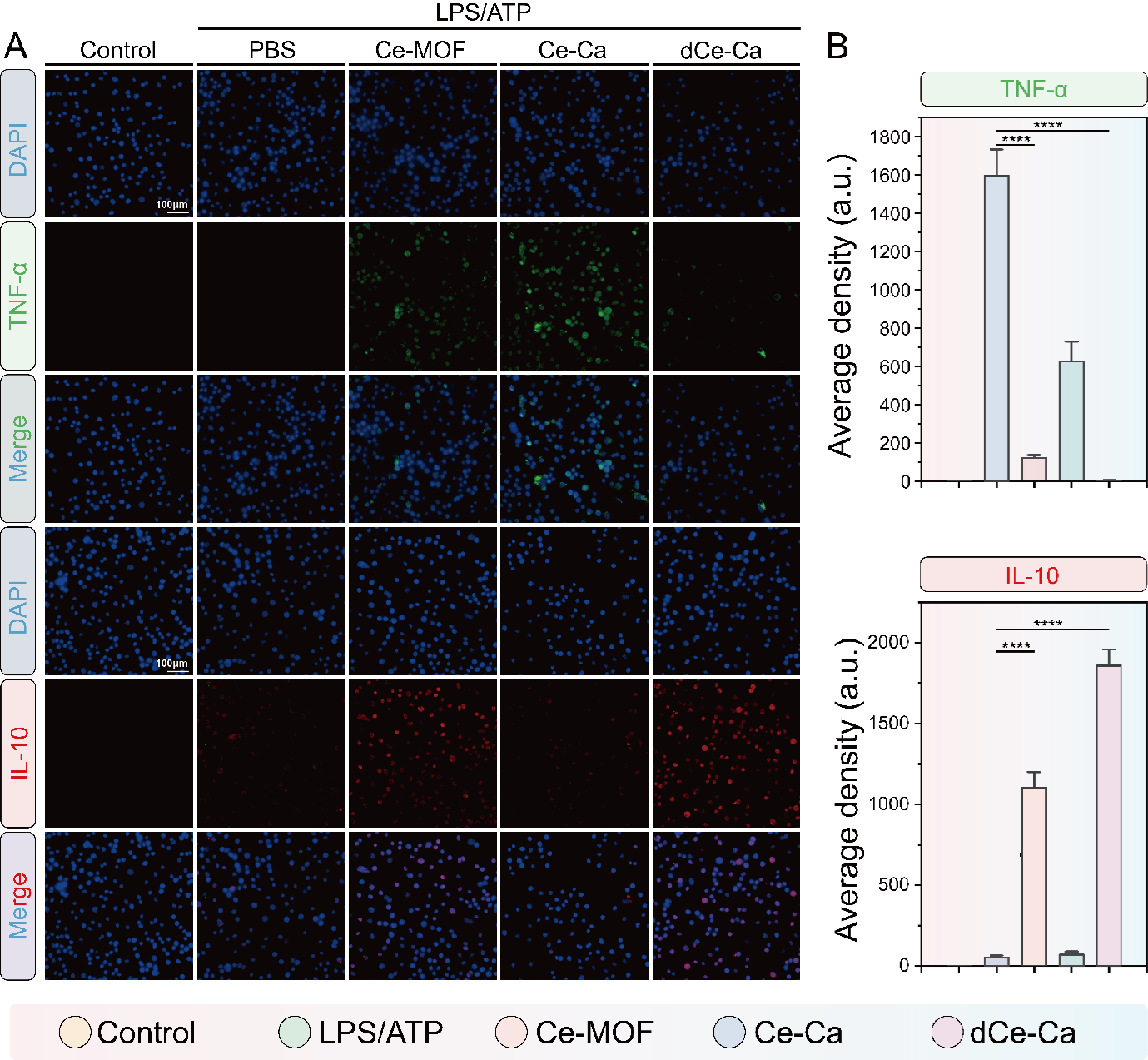


Figure S23 Immunofluorescence staining of IL-10 and TNF-α in different groups and I) Semi-quantitative analysis of fluorescence intensity. Data are presented as mean ± SD, n = 3. Differences were assessed by one-way analysis of variance (ANOVA) followed by Tukey’s multiple comparison test. ****P < 0.0001, ***P < 0.001, **P < 0.01 and *P < 0.05.


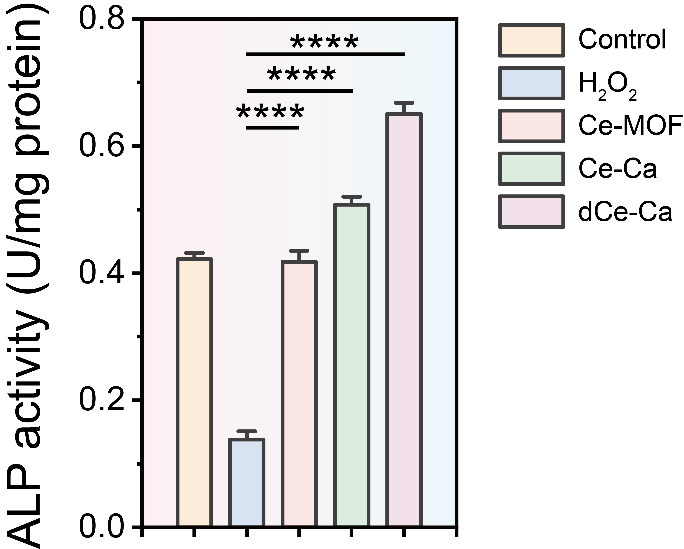


Figure S24 ALP activity in different treatment groups on 7 days. Data are presented as mean ± SD, n = 3. Differences were assessed by one-way analysis of variance (ANOVA) followed by Tukey’s multiple comparison test. ****P < 0.0001, ***P < 0.001, **P < 0.01 and *P < 0.05.


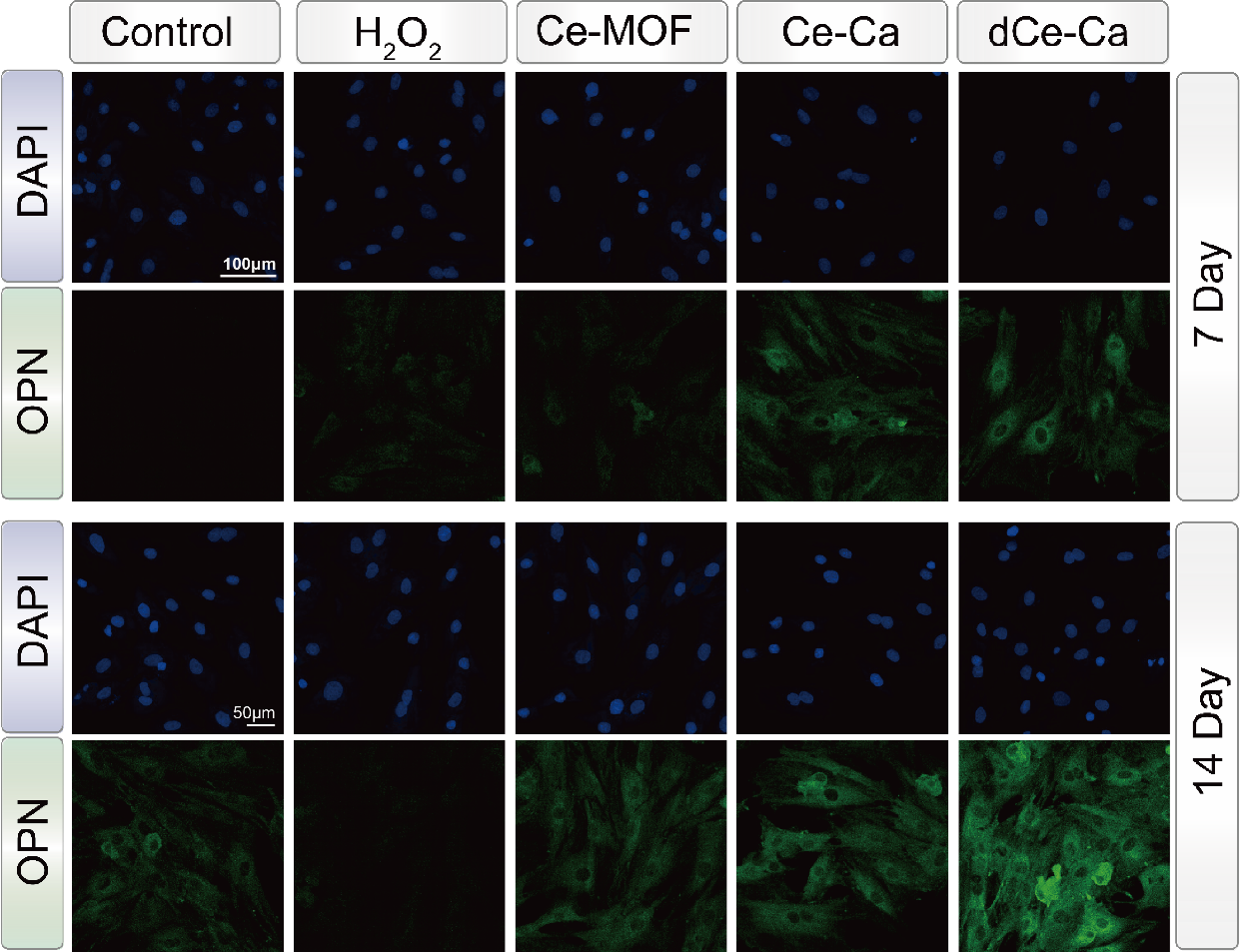


Figure S25 Immunofluorescence staining images of OPN in different groups on 7 days and 14 days.


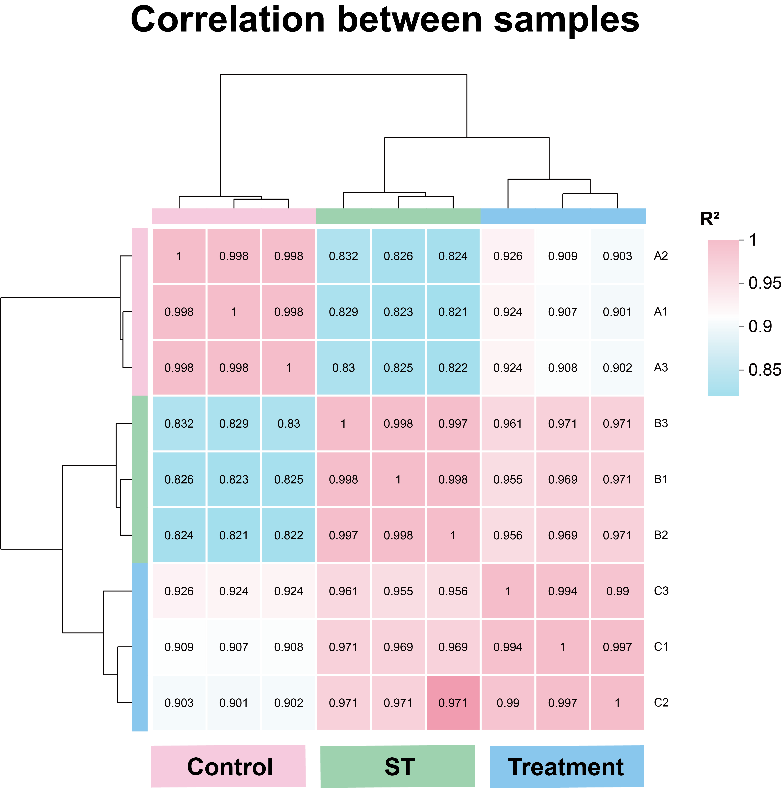


Figure S26 Sample correlation analysis of Pearson correlation coefficients.


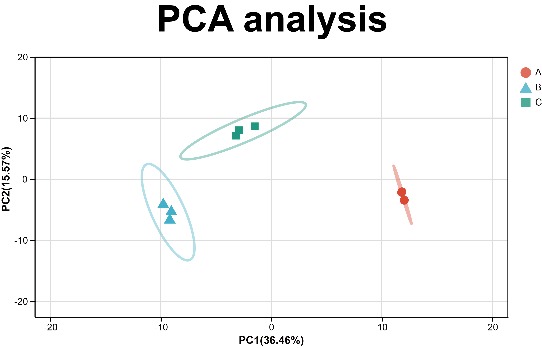


Figure S27 principal component analysis (PCA) of the Transcriptomic Profiles from Groups Control, ST, and Treatment.


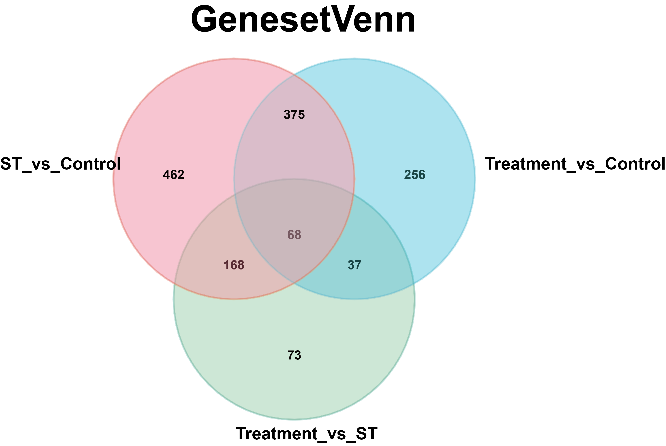


Figure S28 Venn Diagram Among 3 Groups.


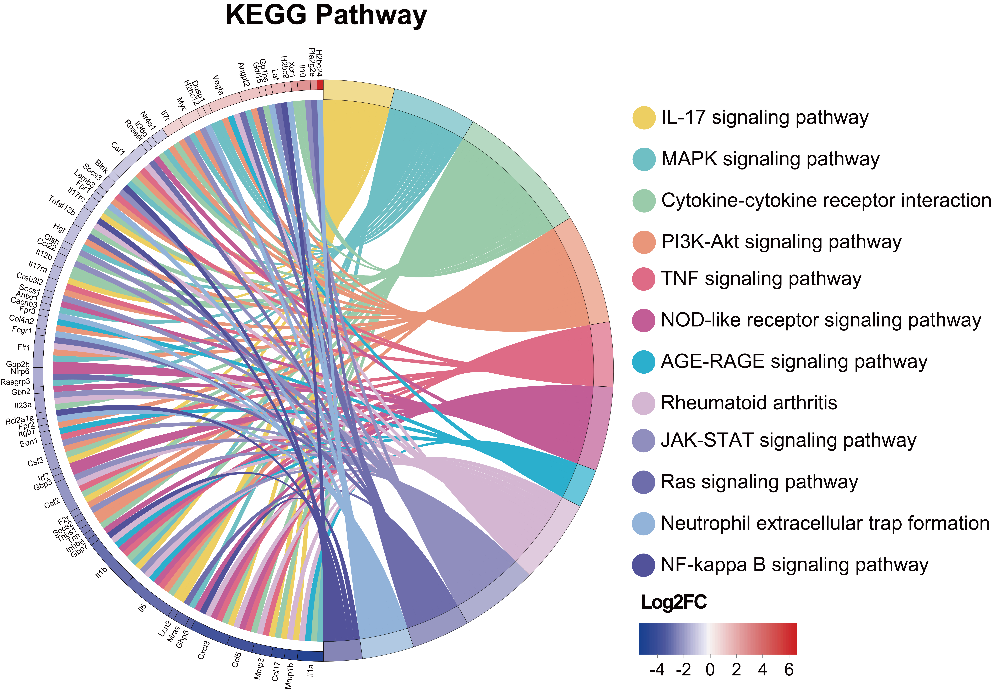


Figure S29 chord plot of genes enriched in the KEGG pathway analysis for the Treatment vs. ST group comparison.


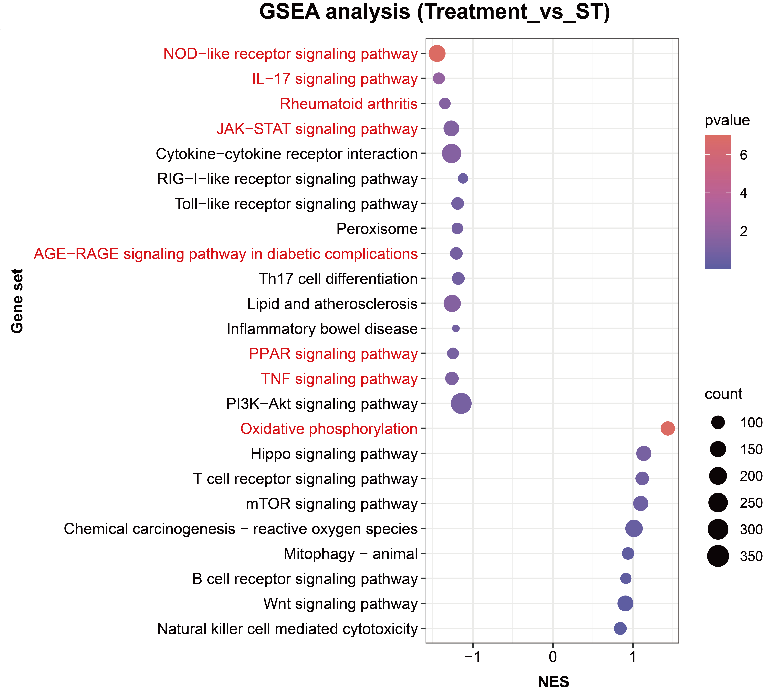


Figure S30 enrichment bubble plot of Gene Set Enrichment Analysis (GSEA) (Treatment vs. ST comparison).


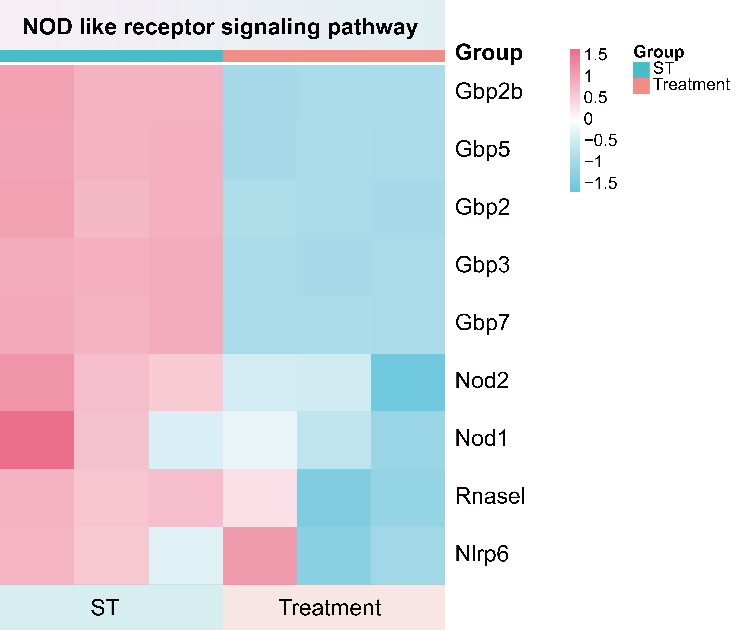


Figure S31 Heatmaps of related genes in NOD-like receptor signaling pathway.


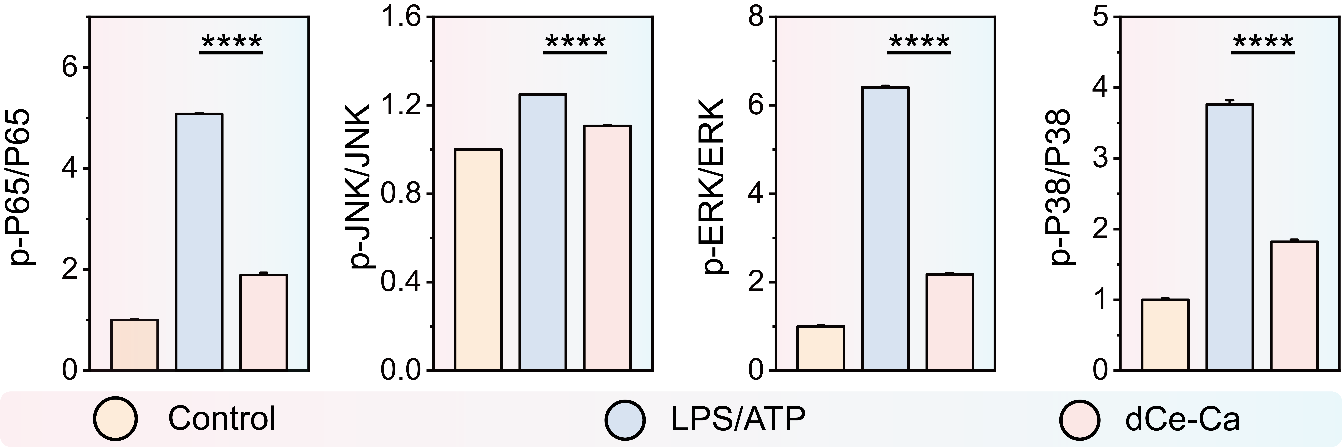


Figure S32 Semi-Quantitative analysis of p-P65, P65, p-JNK, JNK, p-P38, P38, p-ERK, and ERK expression from J774A.1 cells under different treatments. Data are presented as mean ± SD, n = 3. Differences were assessed by one-way analysis of variance (ANOVA) followed by Tukey’s multiple comparison test. ****P < 0.0001, ***P < 0.001, **P < 0.01 and *P < 0.05.


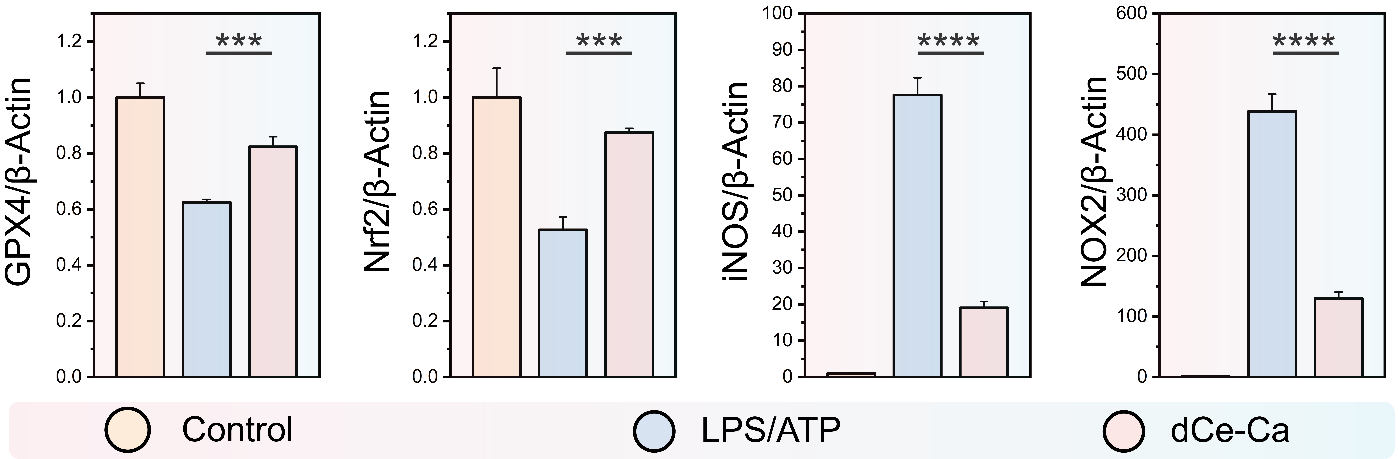


Figure S33 Semi-Quantitative analysis of iNOS, NOX2, and GPX4 expression in whole-cell lysates, and Nrf2 expression in nuclear extracts from J774A.1 cells under different treatments. Data are presented as mean ± SD, n = 3. Differences were assessed by one-way analysis of variance (ANOVA) followed by Tukey’s multiple comparison test. ****P < 0.0001, ***P < 0.001, **P < 0.01 and *P < 0.05.


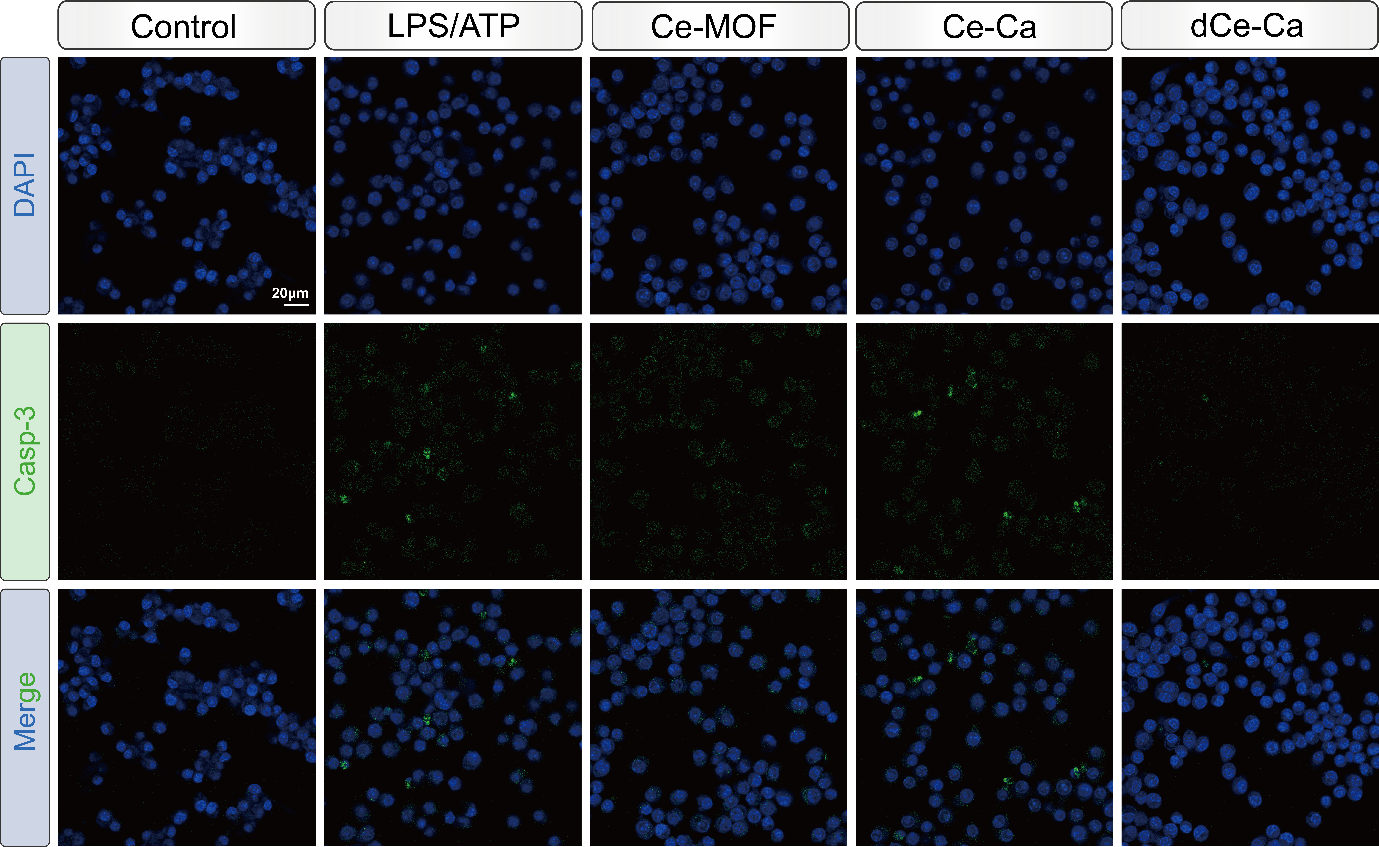


Figure S34 Immunofluorescence staining of cleaved-Caspase-3 in J774A.1 cells after different treatments.


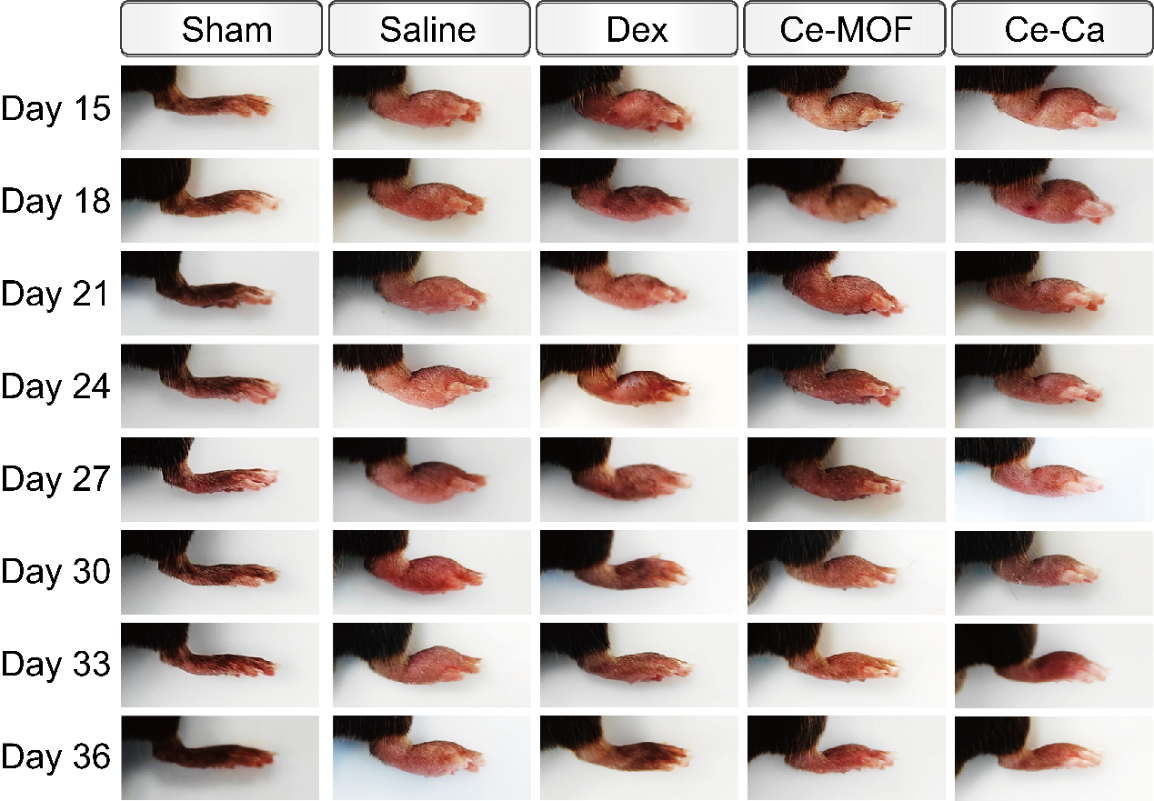


Figure S35 Representative photographs of hind paws from the different treatment groups during the therapeutic period.


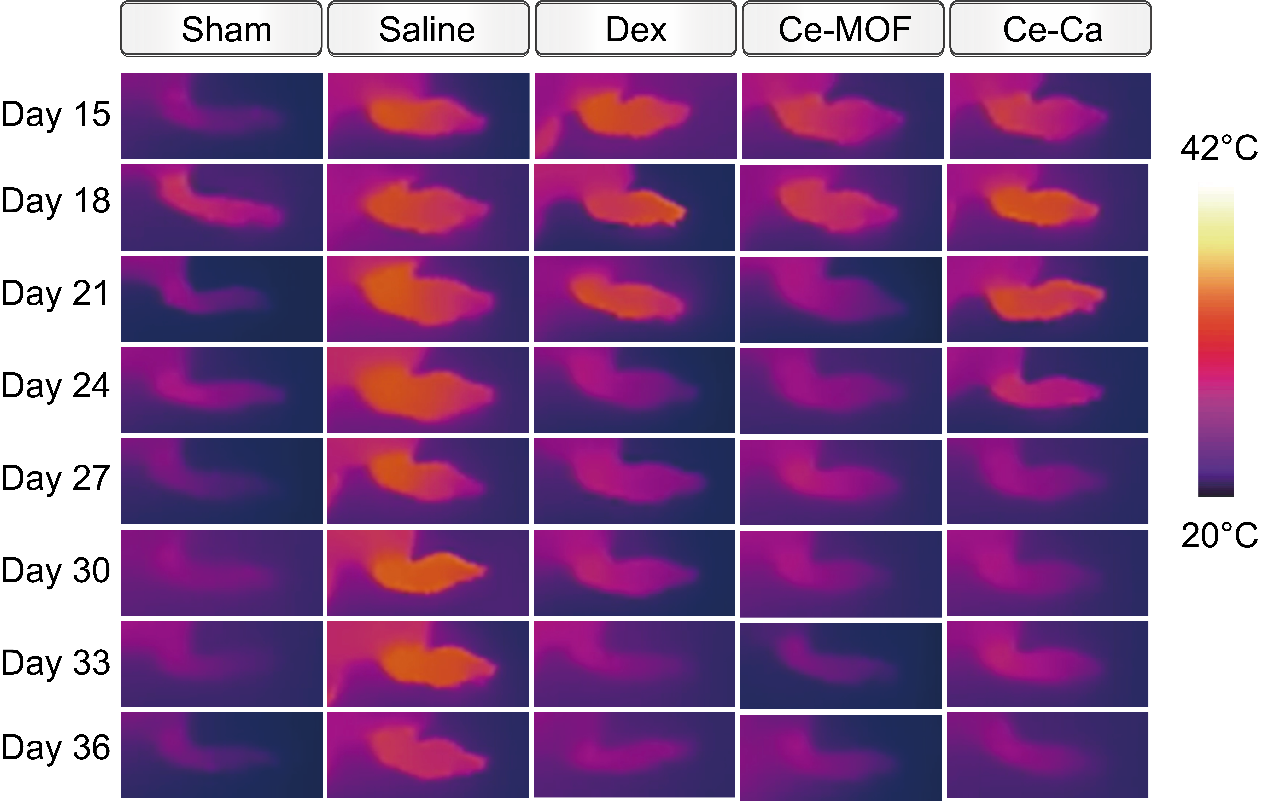


Figure S36 Infrared thermographic images of mouse hind paws in different mouse groups.


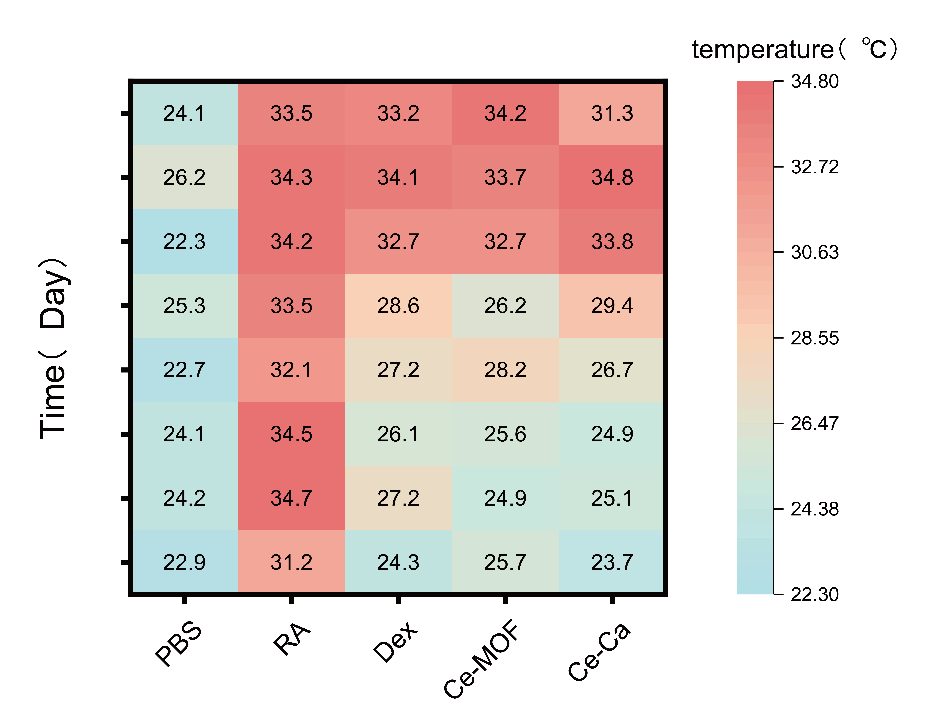


Figure S37 Infrared thermographic assessment of ankle temperature in different mouse groups.


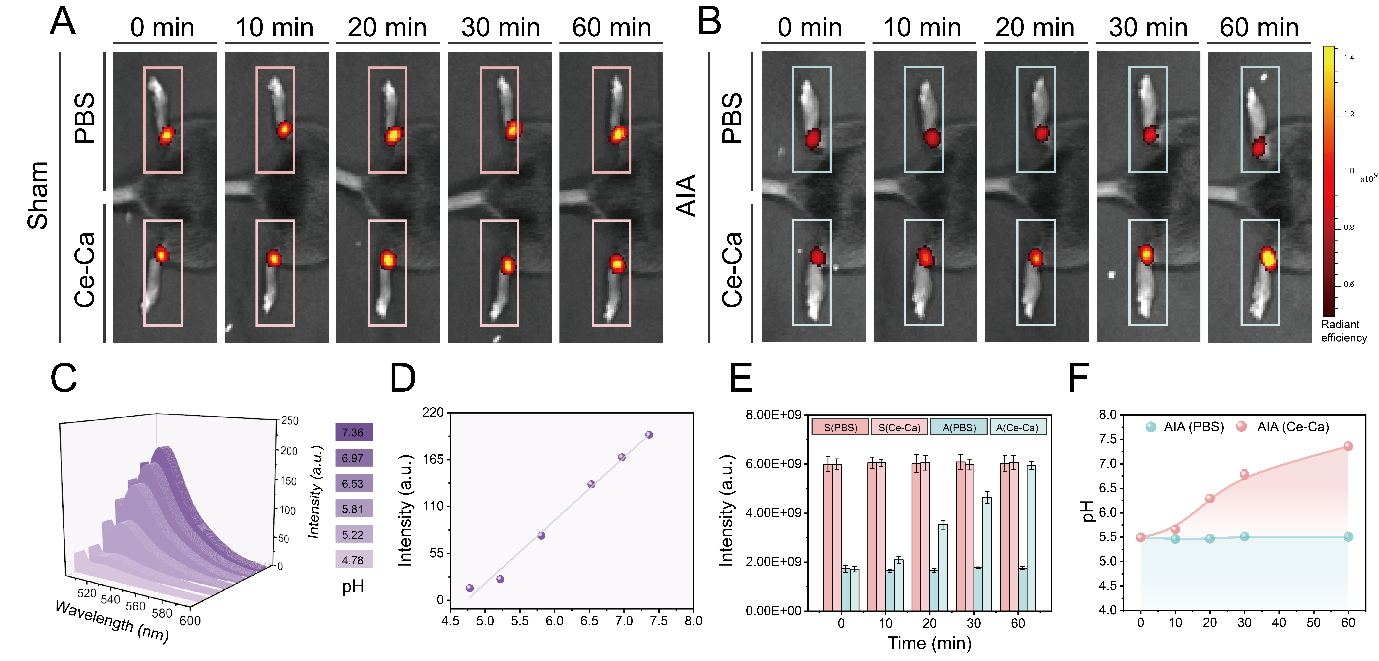


Figure S38 *In vivo* pH imaging of joint cavities in A) sham-operated mice and B) AIA mice after intra-articular injection of PBS and Ce-Ca, respectively, using a pH-responsive fluorescent probe. C) Fluorescence emission spectra of the probe in PBS at different pH values. D) Calibration curve of fluorescence intensity at 495 nm versus pH. E) Semi-quantitative analysis of fluorescence signals from panels A and B. F) Temporal changes in joint cavity pH derived from the semi-quantitative data. Data are presented as mean ± SD, n = 3.


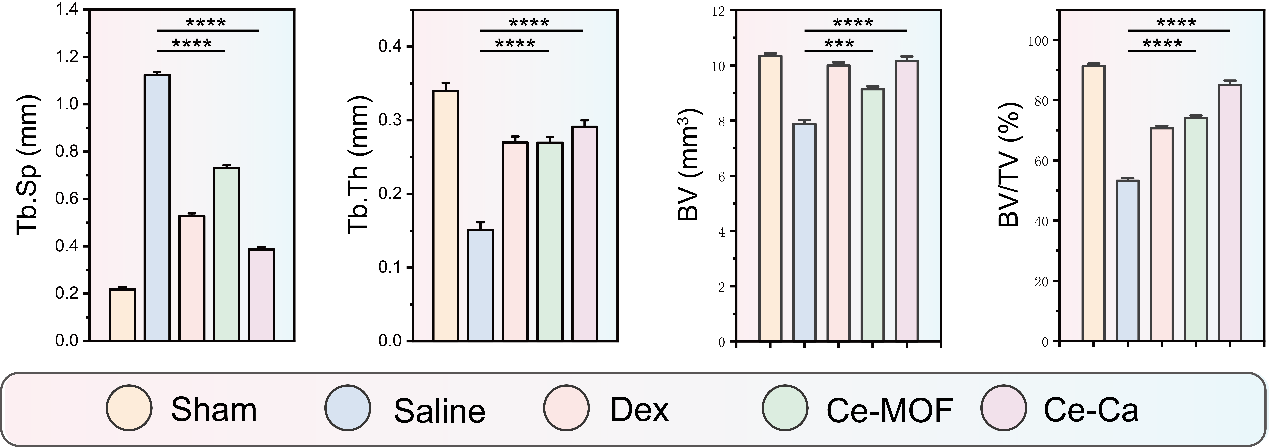


Figure S39 quantitative analysis of the micro-CT data (BV, bone volume; TV, tissue volume; [Tb. Th](https://tb.th/), trabecular thickness; Tb. Sp, trabecular separation. Data are presented as mean ± SD, n = 3. Differences were assessed by one-way analysis of variance (ANOVA) followed by Tukey’s multiple comparison test. ****P < 0.0001, ***P < 0.001, **P < 0.01 and *P < 0.05.


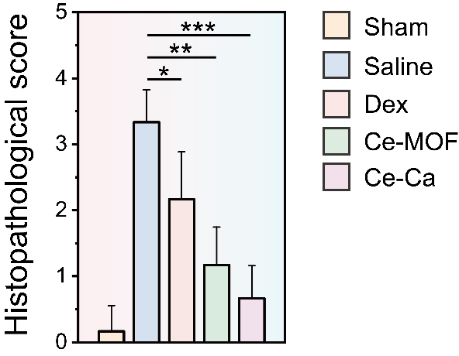


Figure S40 Histopathological scores of different treatment groups based on the severity of synovial hyperplasia, inflammatory infiltration, cartilage damage, and bone destruction. Data are presented as mean ± SD, n = 4. Differences were assessed by one-way analysis of variance (ANOVA) followed by Tukey’s multiple comparison test. ****P < 0.0001, ***P < 0.001, **P < 0.01 and *P < 0.05.


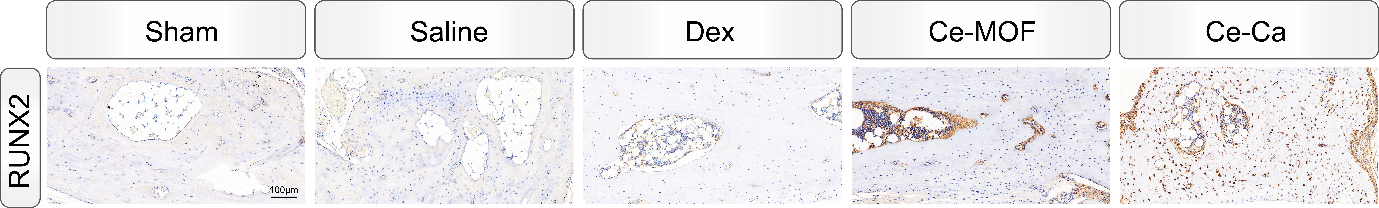


Figure S41 Immunohistochemical staining of RUNX2 in joint sections across groups.


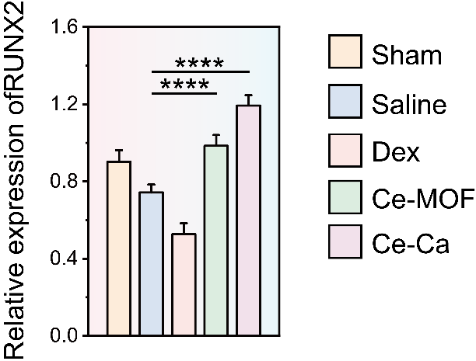


Figure S42 Semi-quantitative analysis of RUNX2. Data are presented as mean ± SD, n = 3. Differences were assessed by one-way analysis of variance (ANOVA) followed by Tukey’s multiple comparison test. ****P < 0.0001, ***P < 0.001, **P < 0.01 and *P < 0.05.


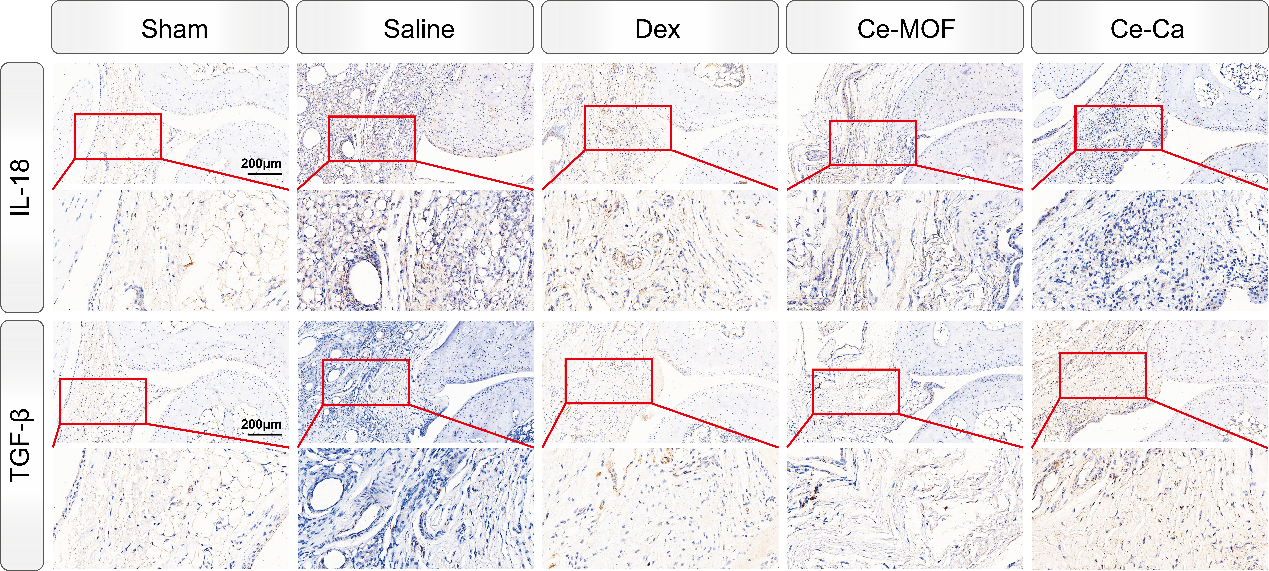


Figure S43 Immunohistochemical staining of IL-18 and TGF-β in joint sections across groups. Magnified views of the synovial regions outlined in red are displayed directly below their respective panels.


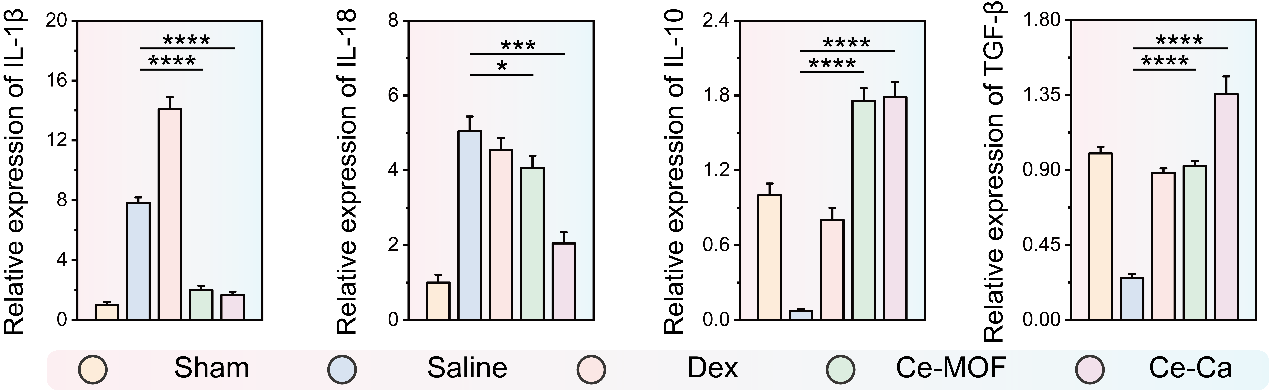


Figure S44 Semi-quantitative analysis of IL-1β, IL-18, IL-10 and TGF-β. Data are presented as mean ± SD, n = 3. Differences were assessed by one-way analysis of variance (ANOVA) followed by Tukey’s multiple comparison test. ****P < 0.0001, ***P < 0.001, **P < 0.01 and *P < 0.05.


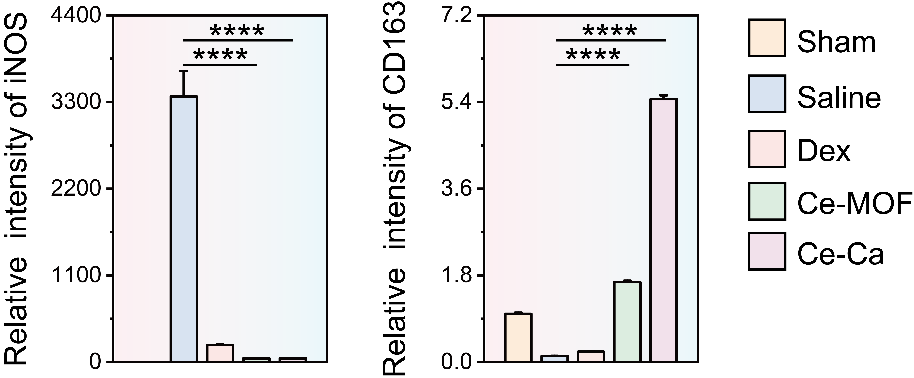


Figure S45 Semi-quantitative analysis of fluorescence intensity (iNOS, CD86). Data are presented as mean ± SD, n = 3. Differences were assessed by one-way analysis of variance (ANOVA) followed by Tukey’s multiple comparison test. ****P < 0.0001, ***P < 0.001, **P < 0.01 and *P < 0.05.


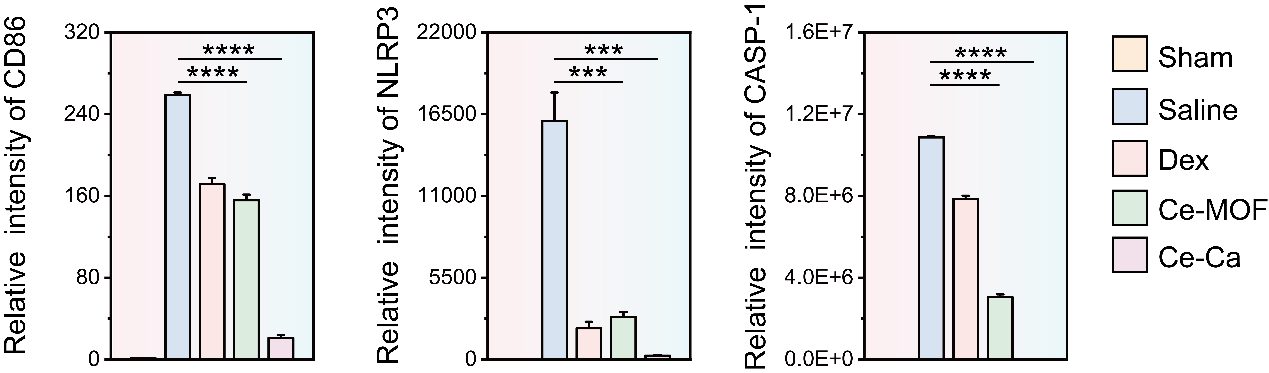


Figure S46 Semi-quantitative analysis of fluorescence intensity (CD86, NLRP3 and Caspase-1). Data are presented as mean ± SD, n = 3. Differences were assessed by one-way analysis of variance (ANOVA) followed by Tukey’s multiple comparison test. ****P < 0.0001, ***P < 0.001, **P < 0.01 and *P < 0.05.


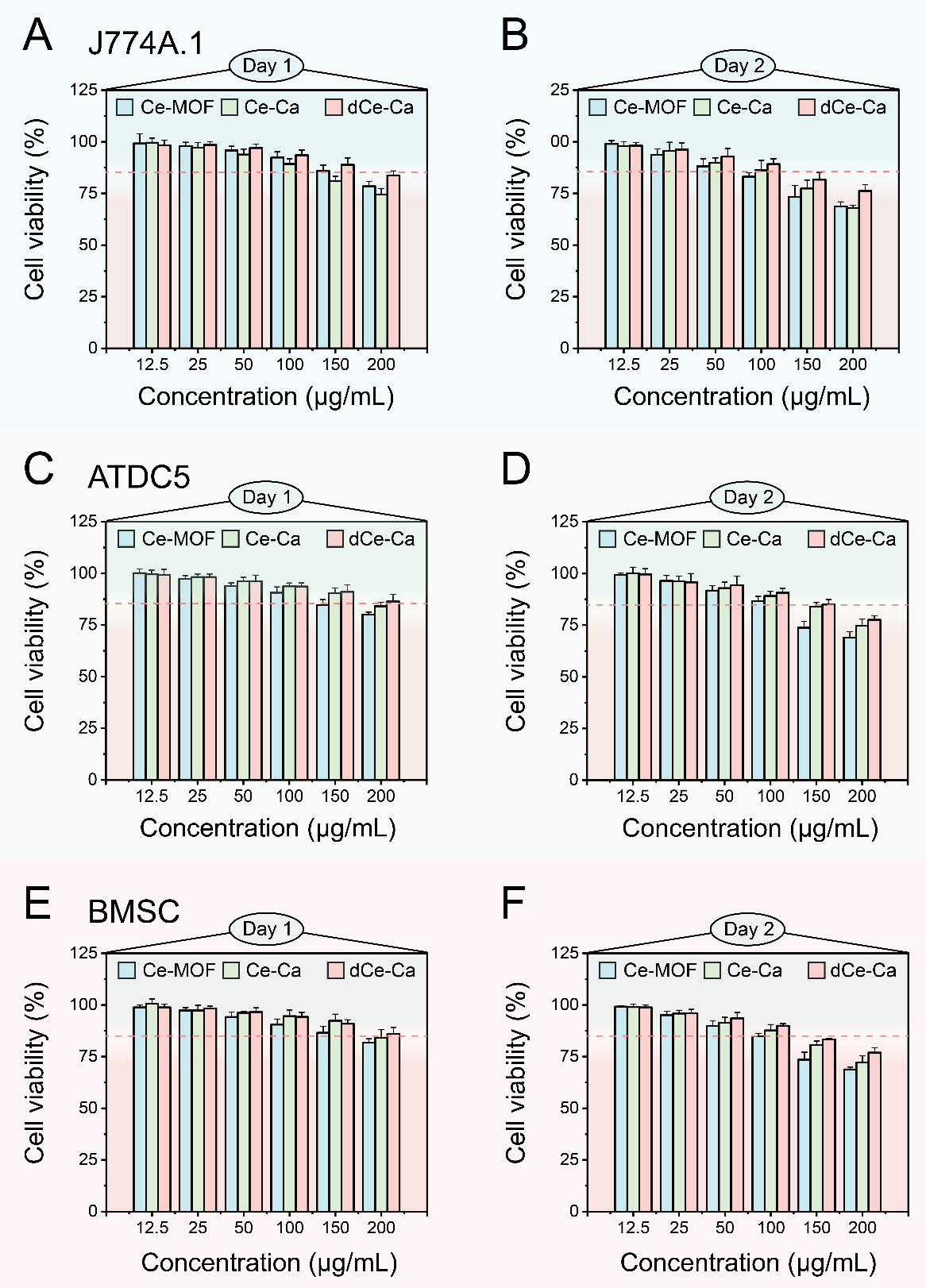


Figure S47 Viability of different cells (J774A.1, ATDC5 and rBMSCs) with different treatment materials as determined by cck-8 assay. Data are presented as mean ± SD, n = 3. Differences were assessed by one-way analysis of variance (ANOVA) followed by Tukey’s multiple comparison test. ****P < 0.0001, ***P < 0.001, **P < 0.01 and *P < 0.05.


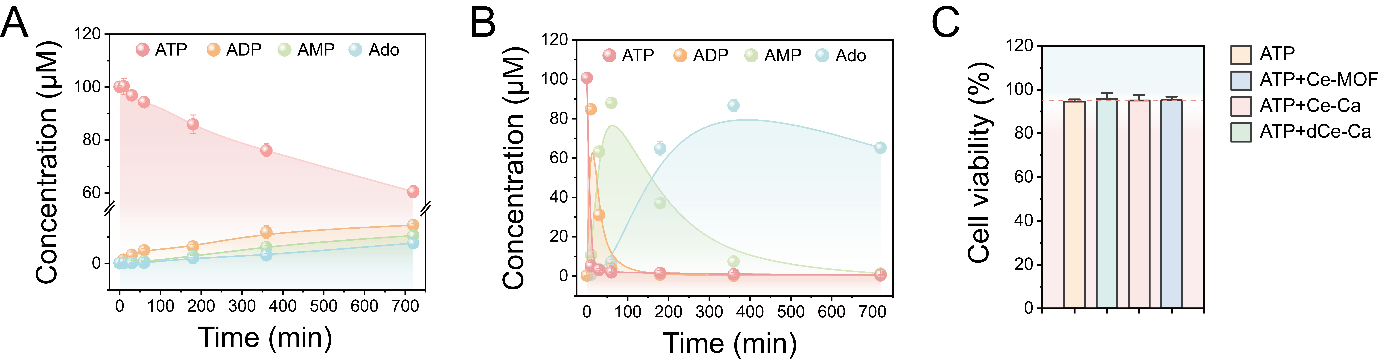


Figure S48 Time-dependent changes in the concentrations of ATP, ADP, AMP, and adenosine (Ado) in A) control and B) dCe-Ca treated groups; C) Cell viability of J774A.1 cells after incubation with 100 μM ATP and different materials (ATP + Ce-MOF, ATP + Ce-Ca, ATP + dCe-Ca) as measured by CCK-8 assay. Data are presented as mean ± SD, n = 3. Differences were assessed by one-way analysis of variance (ANOVA) followed by Tukey’s multiple comparison test. ****P < 0.0001, ***P < 0.001, **P < 0.01 and *P < 0.05.


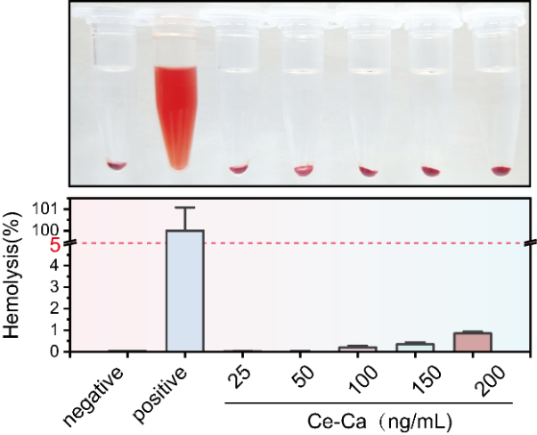


Figure S49 *In vitro* hemocompatibility evaluation of the materials by hemolysis assay. Data are presented as mean ± SD, n = 3. Differences were assessed by one-way analysis of variance (ANOVA) followed by Tukey’s multiple comparison test. ****P < 0.0001, ***P < 0.001, **P < 0.01 and *P < 0.05.


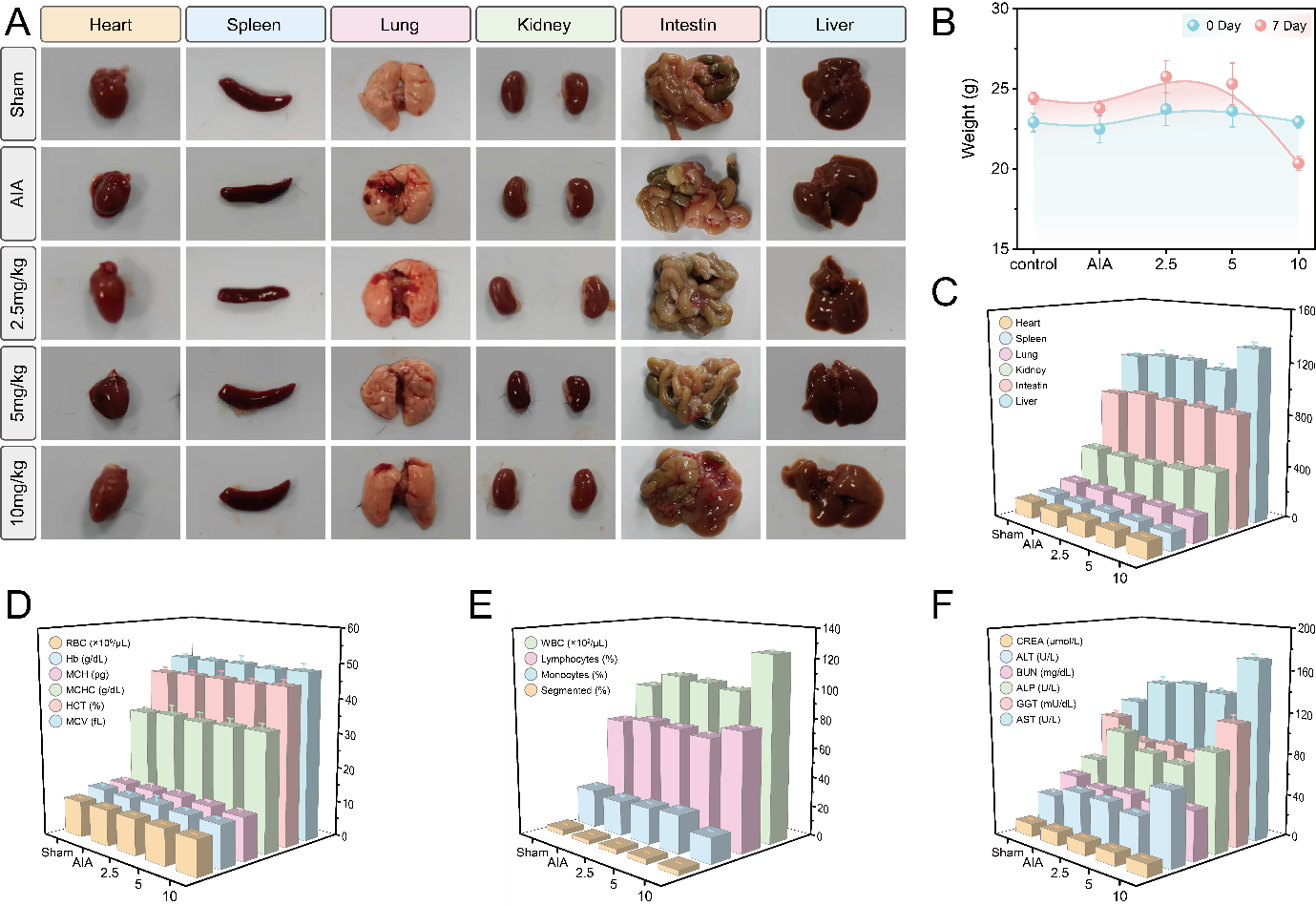


Figure S50 Acute toxicity evaluation of Ce-Ca nanomaterials in mice over 7 days. A) Gross morphology of major organs (heart, liver, spleen, lung, kidney, intestine) from mice in each group. B) Body weight changes of mice in each group over 7 days. C) Wet weight of major organs from mice in each group. D) Hematological parameters: red blood cells (RBC), hemoglobin (Hb), hematocrit (HCT), mean corpuscular volume (MCV), mean corpuscular hemoglobin (MCH), and mean corpuscular hemoglobin concentration (MCHC). E) Hematological parameters: white blood cells (WBC), lymphocytes, monocytes, and segmented neutrophils. F) Liver and kidney function parameters: alanine aminotransferase (ALT), aspartate aminotransferase (AST), alkaline phosphatase (ALP), creatinine (CREA), blood urea nitrogen (BUN), and gamma-glutamyl transferase (GGT). Data are presented as mean ± SD, n = 3. Differences were assessed by one-way analysis of variance (ANOVA) followed by Tukey’s multiple comparison test. ****P < 0.0001, ***P < 0.001, **P < 0.01 and *P < 0.05.


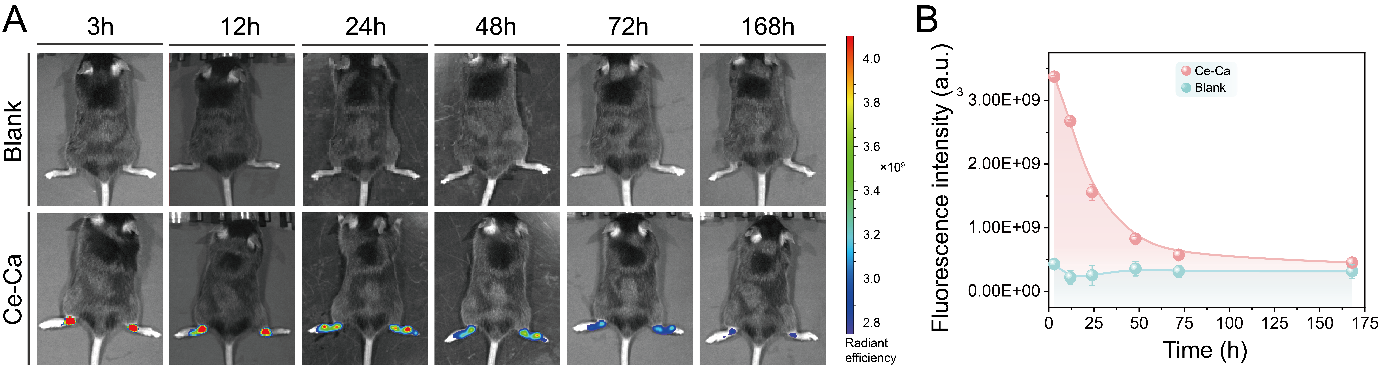


Figure S51 *In vivo* A) fluorescence imaging and B) semi-quantitative analysis of ICG-loaded Ce-Ca nanomaterials after intra-articular injection in mice. Data are presented as mean ± SD, n = 3. Differences were assessed by one-way analysis of variance (ANOVA) followed by Tukey’s multiple comparison test. ****P < 0.0001, ***P < 0.001, **P < 0.01 and *P < 0.05.


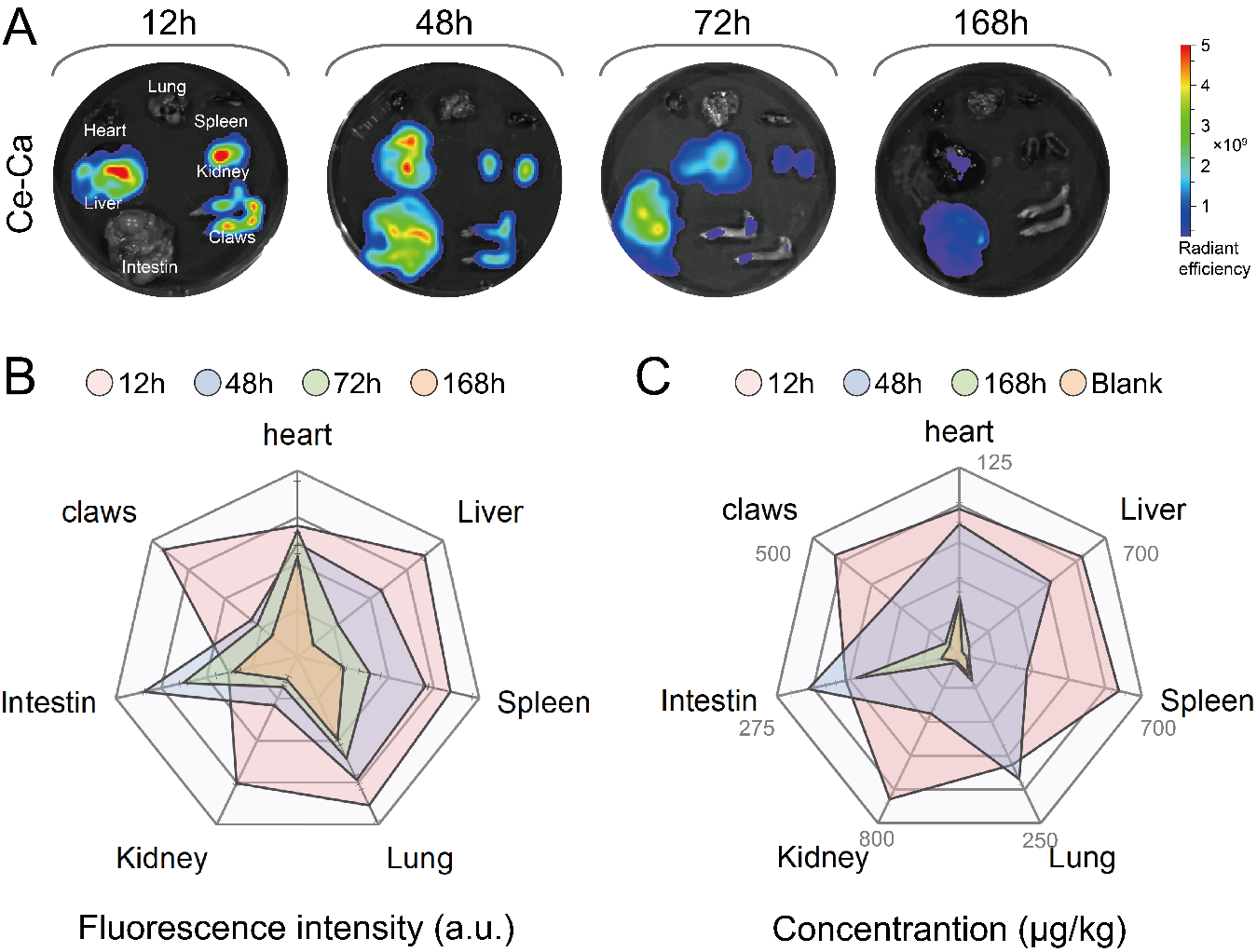


Figure S52 A) *Ex vivo* fluorescence imaging of major organs (heart, liver, spleen, lung, kidney, intestine) and paws at 12 h, 48 h, 72 h, and 168 h after intra-articular injection of ICG-loaded Ce-Ca. B) Semi-quantitative radar chart of Ce-Ca distribution in organs and paws based on fluorescence intensity. C) Radar chart of Ce element content in organs and paws based on ICP-MS detection. Data are presented as mean ± SD, n = 3. Differences were assessed by one-way analysis of variance (ANOVA) followed by Tukey’s multiple comparison test. ****P < 0.0001, ***P < 0.001, **P < 0.01 and *P < 0.05.


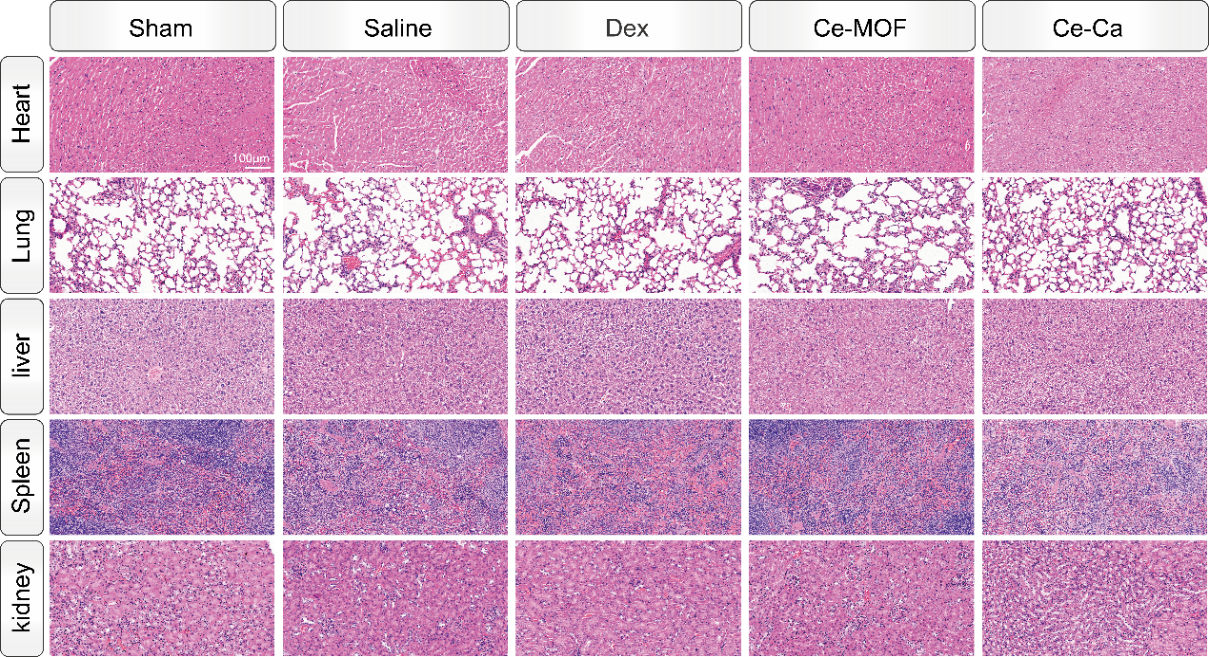


Figure S53 Representative H&E-stained sections of heart, liver, spleen, lung, and kidney from different groups after 21 days.


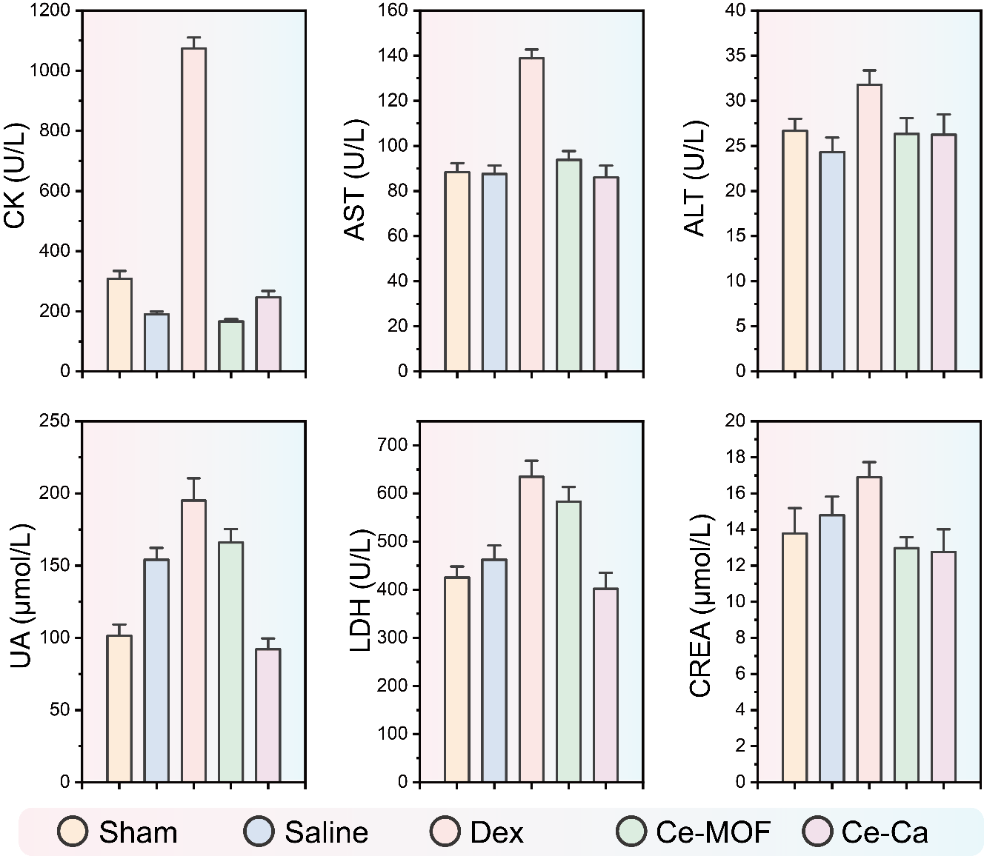


Figure S54 analysis of hematological parameters in mice from different treatment groups after 21 days. Data are presented as mean ± SD, n = 3. Differences were assessed by one-way analysis of variance (ANOVA) followed by Tukey’s multiple comparison test. ****P < 0.0001, ***P < 0.001, **P < 0.01 and *P < 0.05.
